# STEMIN and YAP5SA synthetic modified mRNAs regenerate and repair infarcted mouse hearts

**DOI:** 10.20517/jca.2022.20

**Published:** 2022-06-15

**Authors:** Siyu Xiao, Rui Liang, Emilio Lucero, Bradley K. McConnell, Zhishi Chen, Jiang Chang, Stephen Navran, Robert J. Schwartz, Dinakar Iyer

**Affiliations:** 1 Department of Biology and Biochemistry, University of Houston, Houston, TX 77204, USA; 2 Department of Pharmacological and Pharmaceutical Sciences, College of Pharmacy, University of Houston, Houston, TX 77204, USA; 3 Center for Genomic and Precision Medicine, Department of Translational Medical Sciences, Institute of Biosciences and Technology, Texas A&M University, Houston, TX 77030, USA; 4 Animatus Biosciences LLC, Houston, TX 77054, USA

**Keywords:** Cardiac regeneration, synthetic mRNA, heart delivery, serum response factor, STEMIN, hippo pathway, YAP5SA

## Abstract

**Introduction:**

The adult heart lacks the regenerative capacity to self-repair. Serum response factor (SRF) is essential for heart organogenesis, sarcomerogenesis, and contractility. SRF interacts with co-factors, such as NKX2.5 and GATA4, required for cardiac specified gene activity. ETS factors such as ELK1 interact with SRF and drive cell replication. To weaken SRF interactions with NKX2.5 and GATA4, one mutant, SRF153(A3) named STEMIN, did not bind CArG boxes, yet induced stem cell factors such as NANOG and OCT4, cardiomyocyte dedifferentiation, and cell cycle reentry. The mutant YAP5SA of the Hippo pathway also promotes cardiomyocyte proliferation and growth.

**Aim:**

Infarcted adult mouse hearts were injected with translatable STEMIN and YAP5SA mmRNA to evaluate their clinical potential,

**Methods and Results:**

Mice were pulsed one day later with alpha-EDU and then heart sections were DAPI stained. Replicating cells were identified by immuno-staining against members of the DNA replisome pathway that mark entry to S phase of the cell cycle. Echocardiography was used to determine cardiac function following infarcts and mRNA treatment. To monitor cardiac wall repair, microscopic analysis was performed, and the extent of myocardial fibrosis was analyzed for immune cell infiltration. Injections of STEMIN and YAP5SA mmRNA into the left ventricles of infarcted adult mice promoted a greater than 17-fold increase in the DAPI stained and alpha-EDU marked cardiomyocyte nuclei, within a day. We observed de novo expression of phospho-histone H3, ORC2, MCM2, and CLASPIN. Cardiac function was significantly improved by four weeks post-infarct, and fibrosis and immune cell infiltration were diminished in hearts treated with STEMIN and YAP5SA mmRNA than each alone.

**Conclusion:**

STEMIN and YAP5SA mmRNA improved cardiac function and myocardial fibrosis in left ventricles of infarcted adult mice. The combinatorial use of mmRNA encoding STEMIN and YAP5SA has the potential to become a powerful clinical strategy to treat human heart disease.

## INTRODUCTION

Decades of research and thousands of publications have been dedicated to the singular goal of promoting adult mammalian cardiomyocytes (CM) to re-enter the cell cycle and complete mitosis. The loss of CM underlies most causes of heart failure. Normal repair processes are inadequate to deal with extensive myocardial damage. While heart transplantation is the standard for treatment, the limited availability of donor hearts and the risk of rejection restrict its widespread use. Recently, the prospect of repairing damaged myocardium using stem cells has emerged. Various stem cell types from which CM could be derived have been proposed including embryonic stem cells (ESC)^[1]^, induced pluripotent stem cells (iPSC)^[2]^, and bone marrow-derived mesenchymal stem cells (MSCs)^[3]^. However, developing therapies with these cell types presents problems. For ESCs, ethical issues, potential for teratoma formation, and the need for immunosuppression are also obstacles. iPSCs, although they can be autologous, have similar risk of teratoma formation. Cell therapies such as bone marrow MSCs have been reported to improve ventricular contractile function, but the benefits have been modest and not due to the generation of new CM^[4,5]^.

The heart is the first functional organ that develops during the embryogenesis of vertebrates and is absolutely dependent on SRF to generate sarcomeres and the first heart beat^[6]^. We were among the first to show that SRF activity controls sarcomerogenesis in mouse embryos^[6]^. The ability for SRF to be the universal “myogenic driver” was totally abrogated in conditional *Nkx2.5* *Cre*-induced *Srf*-null embryos and supported the concept that SRF resides at the highest point in the regulatory hierarchy governing sarcomerogenesis^[6]^ and the beginning blockade for cell replication. During mouse heart development, many of the sarcomeric proteins transcriptionally regulated through SRF are sequentially assembled into a complex contractile apparatus, with the sarcomere being its most basic unit, to generate the force needed for contraction^[7,8]^. Fetal CM proliferate by two consecutive steps. First, sarcomeres must be disassembled to enable chromosome segregation to complete the cell division cycle^[9]^. Next, sarcomeres reassemble after cell division and contraction resumes^[10]^. Postnatal cardiomyocyte cell division further slows down in newborn mice after a week and responds to physiological or pathological challenges after birth through cardiac hypertrophy^[10]^ and not through cell division.

In comparison to early postnatal mice or zebrafish, adult mammalian CM are refractory to mitotic activity^[11,12]^. Recently, short-term *in vivo* transgene induction of reprogramming factors OCT3/4, SOX2, KLF4, and C-MYC (OKSM) rejuvenated senescent organs and extended mouse lifespans^[13]^. In addition, *in vivo* expression of OKSM transgenes caused murine heart cell regeneration^[14]^. Short-term expression of OKSM was sufficient to induce cell replication and rejuvenation^[14]^. Thus, to rejuvenate senescent myocytes as well as expand their number after a cardiac infarct, adult myocytes may need to be taken backwards to a primitive replicative state driven by stem cell factors, as we recently showed^[15]^.

We generated a series of mutated SRF proteins that allows the re-entry of adult myocytes into the cell cycle, the most potent of which we called SRF153(A3) or STEMIN. We tested STEMIN synthetic mmRNA in cultures of rat myocytes, which promoted partial reprogramming to a stem-like state, activated NANOG and OCT4, and induced DNA synthesis^[15]^. Because the translation product STEMIN does not recognize its cognate DNA binding target, the CArG box, or serum response element or interactions with co-factors NKX2.5 and GATA4, it blocked the synthesis of many sarcomeric contractile proteins. Recently, transgene expression of Yes-associated protein isoform 1 (Yap1) promoted cardiomyocyte growth *in vivo*^[16]^. In addition, we showed that STEMIN is powerfully assisted at each step by the Hippo pathway factor YAP5SA mutant in our myocyte test system^[15]^. Therefore, STEMIN drives stem cell factors and causes cardiac myocytes to dedifferentiate, which complements mutant YAP5SA that drives a myriad of growth factors to proliferate dedifferentiated myocytes. Furthermore, the combination of STEMIN and YAP5SA synthetic mmRNA reprogramed CM *in vitro* to a replicative phenotype, associated with chromatin remodeling of cell cycle, spindle proteins, and DNA packaging genes^[15]^. We tested whether injecting STEMIN and YAP5SA mmRNA treatments individually and in combination directly into the left ventricles of adult mice after Myocardial infarction (MI) would reprogram myocytes to enter the cell cycle by driving the DNA replisome pathway and repair infarcted hearts?

## METHODS

### Synthesis of mRNA

SRF, YAP, STEMIN, and YAP5SA mRNAs were transcribed *in vitro* from linearized T7 plasmid templates using mMESSAGE mMACHINE T7 Transcription Kit (ThermoFisher)^[15]^. *In vitro* transcription reaction was conducted at 37 °C overnight. Lithium chloride (LiCl) precipitation was conducted at −20 °C for 72 h to remove unincorporated nucleotides and most proteins. Purified mRNAs were quantified by NanoDrop Microvolume Spectrophotometers (ThermoFisher) and analyzed by agarose gel electrophoresis. Then, 1 μg mRNA was loaded to the gel and clear bands were observed before mRNAs were tested in cells^[15]^.

### Animals

C57BL/6J mice of both genders were housed and studied in strict accordance with the recommendations in *the Guide for the Care and Use of Laboratory Animals* of the National Institutes of Health (eighth edition)^[17]^. Animals were handled according to Institutional Animal Care and Use Committee (IACUC) protocols 15-055 and 16-015 (University of Houston).

### Myocardial infarction model

MI was induced by left anterior descending (LAD) ligation^[18]^ on Day +0, the starting day of the experiments (i.e., −0.5 h). Mice were put under 2.5% isoflurane inhalation anesthesia and their body temperature kept at 37 °C while the surgery was conducted. Initially, 3% isoflurane was used to anesthetize the animal for intubation. A 20 G intravenous catheter connected to a ventilator was inserted into the mouse trachea through the oral cavity, conducting artificial ventilation at 120 strokes/min at 20 mL/kg/stroke using room air. The mouse heart was exposed through the thoracic cavity opened through the left fourth intercostal space. An 8-0 polypropylene ligature was used to tie the LAD by fine, smooth tipped forceps. Alteration of heart color was observed after the ligation of LAD. A 6-0 polypropylene ligature was used to suture the animal thoracic cavity by layered stitches. The lungs were inflated to displace air. Animals were removed from artificial ventilation and remained in a supervised setting until fully conscious. Animals in both experimental groups and control groups were housed in separate warm cages until recovery.

### *In vivo* mRNA delivery

Synthetic mRNAs were injected into the left ventricular (LV) myocardium 5 min after the LAD ligation of the mouse heart in an open-chest surgery on the starting day of the experiments. Then, 100 μg mRNA together with Lipofectamine MessengerMAX to a total volume of 60 μL was injected into the left ventricle myocardium of the mouse heart.

### Myocardial echocardiography

Myocardial echocardiography (echo) was conducted under anesthesia with 1%-1.5% isoflurane^[19]^. Mice were placed on a warm pad device to keep their body temperature around 37 °C. Warmed echo gel was placed on the chest of the mouse, and the heart was imaged with a linear transducer. Heart rate was controlled at a similar level within each strain of measurement. LV ejection fraction (EF) was measured. Myocardial echocardiography was measured on Day −2 (i.e., −48 h; baseline), Week +1 (i.e., +7 days; midpoint), Week +2 (i.e., +14 days; midpoint), Week +3 (i.e., +21 days; midpoint), and Week +4 (i.e., +28 days; final), relative to the mRNA injection.

### IVIS bioimaging

Bioimaging was used for *in vivo* live tracking of mRNA-luciferase. For experiments using mRNA-luciferase, D-luciferin of 150 mg/kg mouse body weight was injected via subcutaneous injection at 10 min before imaging for the luciferase signal using an IVIS BioImager. D-luciferin was prepared by dissolving 25 mg/mL. Before imaging, the animals were shaved and followed with their complete hair removal in a circular belt area around the animal in the region of the heart using Nair, to prevent unintended absorbance of the luminescence signal. The mice were then placed on their backs for the IVIS Bioimaging.

### Tissue collections

After the *in vivo* assessments, animals were sacrificed and tissues were harvested. After the mouse was euthanized by CO_2_, the intact heart was immediately removed, soaked in PBS, and perfused by PBS through the aorta to remove the blood in the heart. Hearts after MI were collected for Picrosirius red and immunofluorescence staining. The adult mouse hearts were retrograde-perfused through the aorta with Z-FIX (Anatech LTD; Cat# 170) for 15 min and then immersion-fixed overnight with the same solution. Following fixation, tissues were dehydrated through an ethanol gradient and embedded in liquid Paraplast (Sigma-Aldrich). The heart was embedded in the sagittal direction and frozen at −20 °C before sectioning. The microtome was set at 10 μm to section the tissue.

### Histology and immunofluorescence

Picrosirius red (Abcam; Cat# ab245887) and immunofluorescence staining were performed by standard methods using 8 μm paraffin sections. Antibodies used for immunofluorescence staining were F4/80 (Abcam; Cat# ab6640; 1:100), Ly6g (Abcam; Cat# ab25377; 1:100), phospho-histone H3 Ser10 (Millipore; Cat# 06-570; 1:200), anti-Claspin (Thermofisher; Cat# PA5-102840; 1:100), anti-MCM2 (Abcam; Cat# ab4461; 1:100), anti-Orc2 (Thermofisher; (Cat# PA5-70227; 1:100), and anti-ATR (Proteintech; Cat# 19787-1-AP; 1:100).

Sections were blocked in 3% BSA-PBST, and antibodies were diluted using 1% BSA-PBST. Primary antibodies were incubated overnight at 4 °C. Secondary antibodies were incubated for 1 h at room temperature. Slides were stained with DAPI during washing and coverslips mounted with ProLong Diamond Antifade Mountant (Invitrogen; Cat# P36961).

### Microscopy

Brightfield and immunofluorescence images were obtained using a Nikon Ti-E inverted microscope equipped with a DS-Fi1 5 MP color camera (Nikon Instruments). Confocal images were obtained using a Leica SP8 Upright Confocal DM6000 CFS Microscope (Leica Microsystems).

### *In vivo* alpha-EdU assay

Alpha-EdU (10 μg/g of mouse body weight) was injected via subcutaneous injection at 8 h before sacrifice. The intact heart was procured by fixation in 4% paraformaldehyde, stored in 70% ethanol, and then embedment into paraffin for histological assessment. The hearts were cross-sectioned and stained for alpha-EdU by Click-iT EdU Cell Proliferation Kit (ThermoFisher). Slides were imaged with a Leica SP8 confocal microscope.

## RESULTS

### The combination of STEMIN and YAP5SA mmRNA injections increased the replication of cardiomyocyte nuclei in the left ventricle of adult Infarcted mouse hearts.

**Figure 1.**
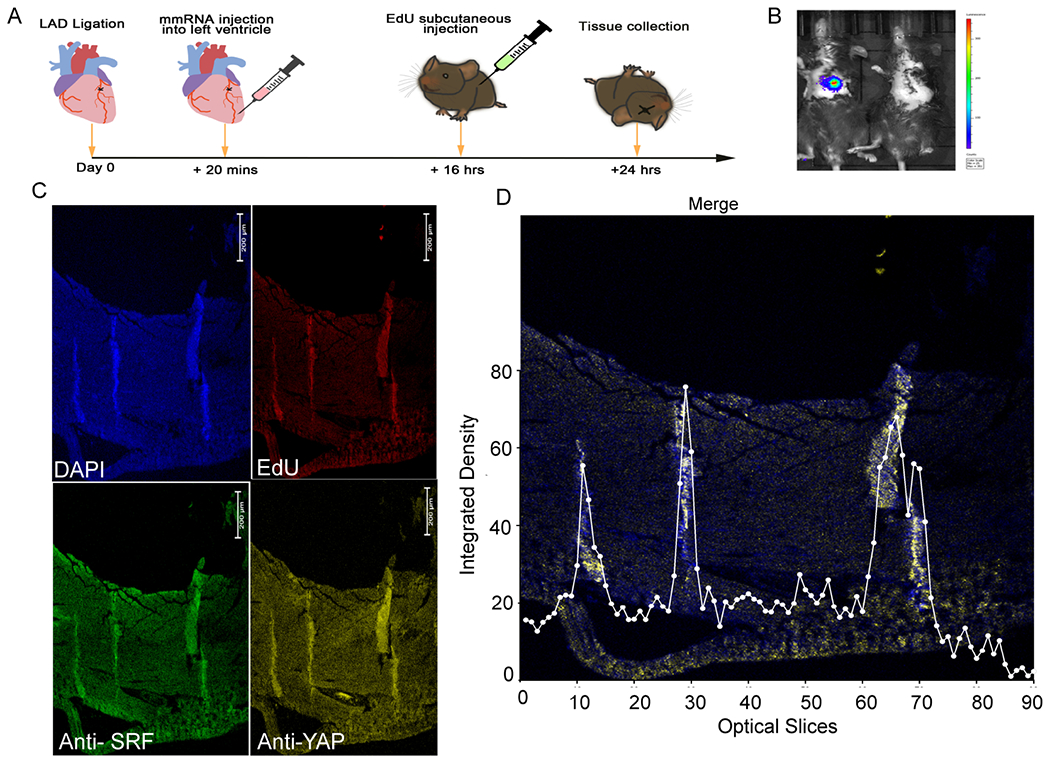
Injected STEMIN and YAP5SA mmRNA induced nuclear replication in infarcted adult mouse hearts. (A) Schematic diagram of short-term (24 h) *in vivo* experiment. A combination of STEMIN and YAP5SA mmRNA was injected in five locations around the infarct in the left ventricle of inbred litter mates (100 days old). (B) Mouse IVIS bioluminescence images 24 h post-injection: (Left) mouse heart injected with 70 μg (50 μL) Luc mmRNA + 32 μL Lipofectamine MessengerMAX; and (Right) non-Luc control mouse heart injected with PBS (50 μL) + 32 μL Lipofectamine MessengerMAX. D-luciferin (150 mg/kg mouse body weight) injected via subcutaneous injection 4 h before light emission analysis to detect luciferase signal using an IVIS BioImager. (C) Images of DAPI and EdU staining of paraformaldehyde fixed left ventricle cardiac sections taken 24 h after five equal injections of STEMIN (50 μg in 38 μL) and YAP5SA (50 μg in 22 μL) with 33 μL Lipofectamine MessengerMAX around the infarct. The control group was injected with PBS (60 μL) and 33 μL Lipofectamine MessengerMAX. EdU (10 μg/g of mouse body weight) was injected via subcutaneous injection at 8 h prior to sacrifice. The intact heart was immediately washed and drained of blood cells, fixed in 4% paraformaldehyde, stored in 70% ethanol, and then embedded into paraffin for histological assessment. Each heart was cross-sectioned and EdU was detected by Click-iT EdU Cell Proliferation Kit (ThermoFisher). Note the overlapping images stained along three needle tracts for DAPI, EdU, Anti-SRF, and Anti-YAP. (D) Merged images of DAPI and EdU staining were first divided into 90 equal optical slices and then integrated density was measured, in which the same threshold was used for all the optical slices.

The protocol of a short-term (24 h) *in vivo* experiment to test synthetic mmRNA injected in five locations around the infarcted region in the left ventricle of inbred C57BL/6J litter mates (approximately 100 days old) is shown as a schematic diagram in Figure 1A. We used live bioimaging tracking of synthetic luciferase mmRNA to exam the mRNA heart delivery methodology in Figure 1B. Multiple injections were used to distribute 70 μg Luc mmRNA with Lipofectamine MessengerMAX. The non-Luc control was injected with PBS and transfection reagent. At 24 h, D-luciferin (150 mg/kg mouse body weight) was injected subcutaneously 4 h before imaging the luciferase signal using an IVIS BioImager. An example from two repeats is presented in Supplementary Figure 1, showing a strong bioluminescence signal in the heart area of the mouse injected with Luc mmRNA [Supplementary Figure 1A and C]. No bioluminescence signal was found in the other organs or tissues in the animal, which further exhibited a precise, leak-free mRNA delivery method.

Next, we conducted a short-term 24 h assay to test whether combined injections of both STEMIN and YAP5SA mmRNA together with Lipofectamine MessengerMAX adjuvant promoted cell proliferation. We followed alpha-EdU incorporation into nuclei of infarcted adult mouse heart *in vivo* by an 8 h alpha-EdU pulse injected before sacrifice [Figure 1C]. Alpha-EdU incorporation was detected in heart cross-sections that followed along needle tracts [Figure 1C and D]. Alpha-EdU detection by the Click-it assay and nuclear DAPI stain clearly overlapped. We used anti-SRF to immuno-detect STEMIN and Anti-YAP to immuno-detect YAP5SA that showed newly synthesized proteins, but not in control hearts injected with adjuvant. Merged images of DAPI and EdU staining were first divided into 90 equal optical slices and then the integrated density was measured, in which the same threshold was used for all the optical slices [Figure 1D] as well as condensed into 30 slices [Supplementary Figure 2]. Confocal microscopy and ImageJ software revealed three peaks of integrated density. Considering the highest integrated peak of 87 units with an adjoining background of 3-5 units, we observed a stunning 17-29-fold increase in the number of DAPI and EdU stained cardiac nuclei [Supplementary Figure 2].

**Figure 2.**
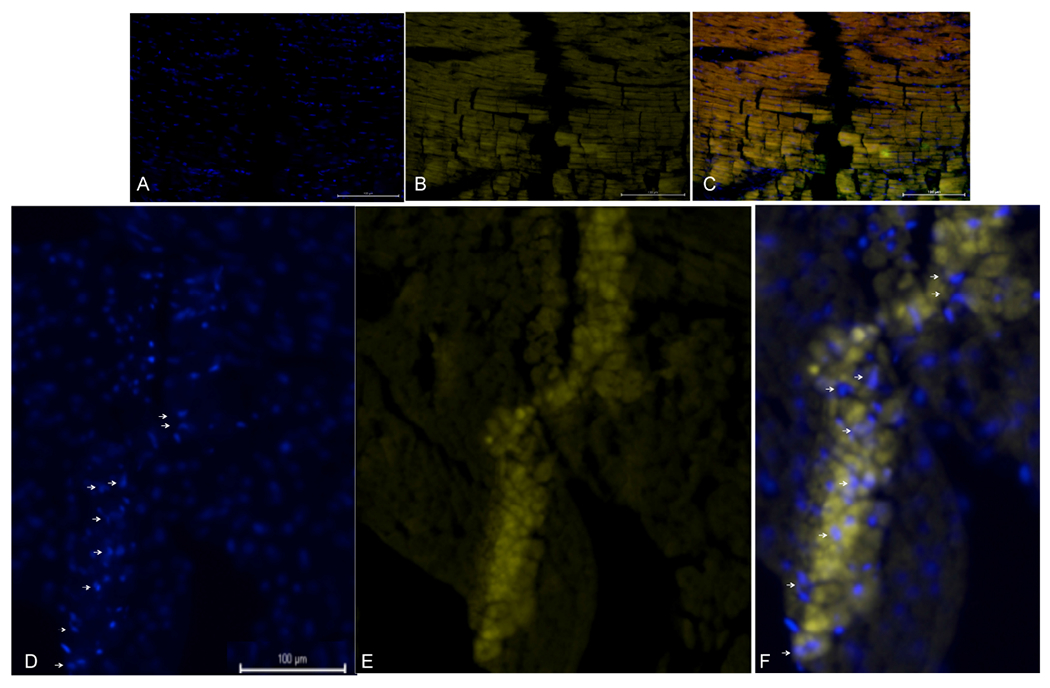
Injected STEMIN and YAP5SA mmRNA induced DAPI and Alpha EdU stained nuclei, but not in control infarcted adult mouse hearts. (A-C) An infarcted control heart injected with only Lipofectamine MessengerMAX and PBS. No DAPI and or alpha-EdU staining was observed along needle tract. (D-F) Another infarcted heart injected with STEMIN and YAP5SA mmRNA together with Lipofectamine MessengerMAX adjuvant. We observed overlapping DAPI and alpha-EdU staining, indicating strong nuclear myocyte proliferation. Arrows point to doublet nuclei.

Figure 2A-C shows an infarcted control heart injected with only Lipofectamine MessengerMAX and PBS. Along the needle tract, there was no discernable increase in DAPI and alpha-EdU staining. In contrast, another infarcted heart was injected with STEMIN and YAP5SA mmRNA together with Lipofectamine MessengerMAX adjuvant. Figure 2D–F reveals robust overlapping DAPI and alpha-EdU staining. Many of the DAPI stained nuclei are doublets indicating strong nuclear myocyte proliferation. White arrows point to the replicated nuclei in Figure 2.

**Figure 3.**
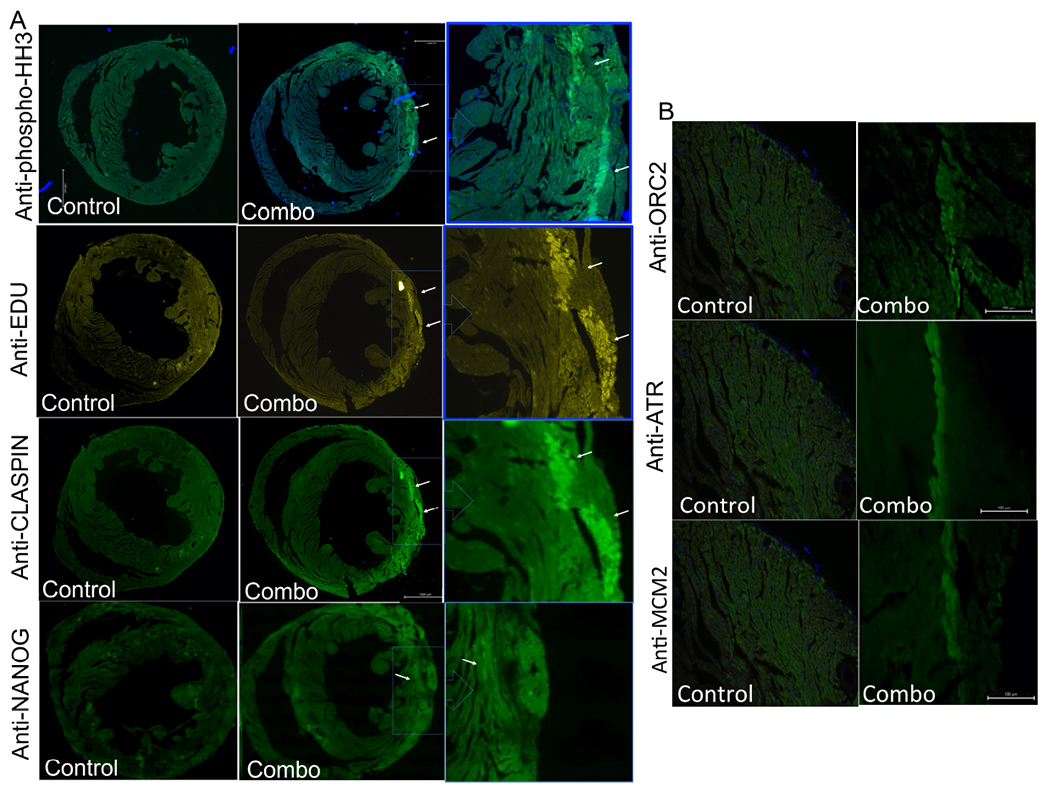
Injected STEMIN and YAP5SA mmRNA induced cell replication and DNA replisome factors. (A) In a second injected heart, anti-phospho-histone H3 stain of a serial section was observed along two needle tracts marked by white arrows. Robust EdU stain overlap anti-pHH3 stains exactly in the same orientation and location. A key component of the DNA replisome was stained by anti-CLASPIN overlapped anti-pHH3 and Edu stains. Anti-NANOG stained NANOG was observed, but peaked levels were probably about 12 h earlier^[15]^. (B) In a third injected heart, anti-ORC2 (Origin recognition complex subunit 2) and anti-MCM2 showed the stained markers of the pre-initiation phase in early G1 stage induced in the injected hearts. Anti-ATR stained Ataxia telangiectasia and Rad3-related, an essential kinase that is active in S phase which overlapped other DNA replisome factors. Thus, STEMIN and YAP5SA mmRNA treatment fostered cell cycle entry by promoting DNA replication in the G1 phase.

In our accompanying publication^[15]^, we showed that the combination treatment of STEMIN and YAP5SA induced stem cell factors, such as NANOG, and cell replication in isolated myocytes. As shown in Figure 3, induced NANOG was observed within 24 h following staining with anti-NANOG, but it may have actually peaked 12 h earlier^[15]^. The eukaryotic DNA replisome is required for rapid and accurate chromosome replication^[20]^. DNA replisome assembly begins in G1 phase of the cell cycle, in which MCM2 (minichromosome maintenance complex component 2-7) is first loaded onto DNA by ORC, Cdc6, and Cdt1. To further test the case for cardiac nuclear replication in injected hearts, we observed markers of cell replication, such as anti-phospho-histoneH3 Ser10 (pHH3), a well-considered marker of DNA replication in cardiac myocytes^[21]^, stained along two needle tracts shown by arrows in Figure 3A. Additional staining of pHH3 is shown in Supplementary Figure 3, where phase contrast images show the needle tract. Anti-pHH3 stained cardiac nuclei undergoing nuclear division and overlapped DAPI and TNNT staining, exactly in the same orientation and location, thus indicating newly replicated nuclei in the combo-treated hearts. Alpha-EdU staining was superimposed on anti-pHH3 stain exactly in the same orientation, indicating DNA synthesis of cardiac myocyte nuclei. Leading to nuclear division, CLASPIN, a key component of the DNA replisome, contains a domain for docking with CHK1 (Checkpoint kinase 1). CLASPIN was highly upregulated by the combination treatment, and anti-CLASPIN showed overlapping staining with anti-pHH3 and alpha-Edu stain [Figure 3A]. In a third injected heart, ORC2 (origin recognition complex subunit 2), which recruits NOC3 (nucleolar complex-associated protein) and MCM2 in the pre-initiation phase in early G1 stage, was induced in the injected hearts, as shown by overlapping needle injection tracts [Figure 3B]. Anti-ATR stained Ataxia telangiectasia and Rad3-related (ATR), an essential kinase in S phase that ensures completion of DNA replication. Thus, STEMIN and YAP5SA mmRNA treatment fostered cell cycle entry by promoting DNA replication in the G1 phase [Figure 3A and B].

### STEMIN and YAP5SA repaired infarcted adult mouse hearts *in vivo*

**Figure 4.**
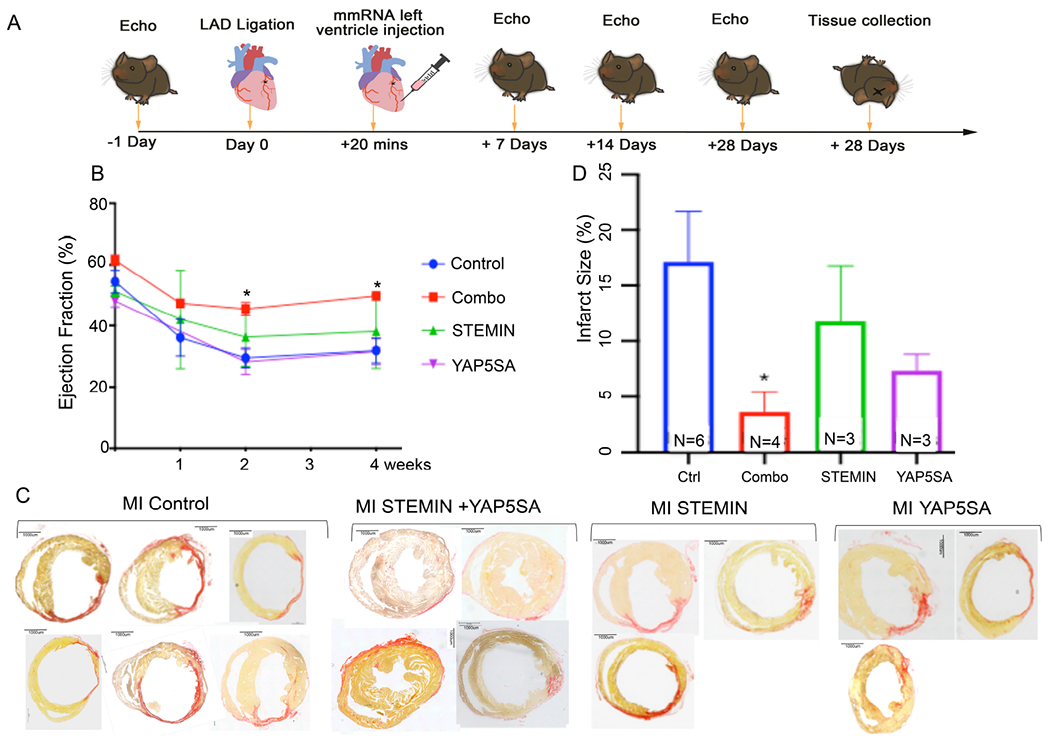
Injections of the combination of STEMIN and YAP5SA mmRNA repaired infarcted adult mouse hearts *in vivo*. (A) Schematic diagram of long-term *in vivo* experiments in adult mice (100 days old) infarcted by LAD ligation. Five equal injections of STEMIN (50 μg in 38 μL) and YAP5SA (50 μg in 22 μL) with 33 μL Lipofectamine MessengerMAX around the cardiac infarct. In addition, Individual synthetic mmRNA were tested for their ability to restore cardiac function following infarctions. The control group was injected with PBS (60 μL) with 33 μL Lipofectamine MessengerMAX. Echo Doppler measurements were taken prior to infarction and one, two, and four weeks after mmRNA injections. (B) Cardiac function was graphed for Echo Doppler measurements of the infarcted controls (six mice) and the combination of STEMIN and YAP5SA mmRNA experimental group (four mice) from the same inbred liter. The EF declined greater for the control than the combination experimental group by the second and fourth weeks, and both were significantly different (*P* < 0.05, two-tailed, shown by *). EF of STEMIN or YAP5SA mmRNA individual injections into infarcted hearts failed to significantly improve cardiac EF above the control levels. STEMIN showed a slight trend upwards and YAP5SA was not better than the infarcted controls. (C) At the end of four weeks, the hearts were removed, sectioned, and stained with Picrosirius red. The infarct zone was marked as pink/red with the Picrosirius staining. (D) Infarct scar area and total area of myocardium were traced and measured by ImageJ. Infarcted size% = Picroirius red + scar area/total ventricular area. Measurements of the infarct zone taken from the combination of STEMIN and YAP5SA mmRNA experimental group injected treatment group (four hearts) compared to all controls (six hearts) showed significant reduction (*P* < 0.05, two-tailed, shown by *). STEMIN injections alone tended to reduce the size of the cardiac infarcts stained by Picrosirius red but were statistically insignificant. YAP5SA mmRNA was not better than STEMIN in cardiac repair.

We next considered whether transient mmRNA injections would repair infarcted hearts. We tested the injection of STEMIN and YAP5SA mmRNA directly into the left ventricles of adult mice following MI. First, MI was induced by left anterior descending (LAD) ligation on Day 0 [Figure 4A]. Next, synthetic mRNAs were injected into the left ventricular (LV) myocardium 5 min after the LAD ligation of the mouse heart in an open-chest surgery on the starting day of the experiments. Synthetic mRNA (100 μg) together with Lipofectamine MessengerMAX in a total volume of 60 μL was split into five equal portions and injected into the LV myocardium. Echocardiography performed at one, two, and four weeks after mmRNA injections [Figure 4B] was graphed for EF of infarcted controls (six mice) and the combined mmRNA group (four mice), all from the same inbred liter. Infarcted controls showed a significant decline in their EF (*P* < 0.05, two-tailed) in comparison to the combination STEMIN and YAP5SA mmRNA treatment group in the second week post-MI [Figure 4B]. The combination treatment group improved significantly (*P* < 0.05) in comparison to the infarcted controls after four weeks, thus indicating significant improved heart function elicited by combined synthetic mmRNA.

At the end of four weeks, the hearts were removed, sectioned, and the infarct zones were stained with Picrosirius red marked as a pink/red color. Measurements of the infarct zone taken from the controls (six hears) showed significant reduction (*P* < 0.05, two-tailed) in comparison to the combination mRNA injected treatment group (four hearts), as shown in Figure 4C. Therefore, STEMIN and YAP5SA mmRNA together repaired infarcted adult mouse hearts *in vivo* with little fibrosis [Figure 4D]. STEMIN mmRNA injections alone reduced the size of the infarcts stained by Picrosirius red, but it was again statistically insignificant. In addition, YAP5SA did not improve infarcted wall thickness in comparison to infarcted controls.

We then compared individual synthetic mmRNAs for their ability to restore cardiac function following infarctions in comparison to the combination of STEMIN and YAP5SA mRNA [Figure 4B and Supplementary Figure 4]. Because the initial EF measurements were variable between mice, weekly differences were compared by Delta EFs between controls and treated mice, as shown in Supplementary Figure 4. In comparison with infarcted controls, STEMIN mmRNA injections alone showed an upward trend, but they did not significantly improve cardiac EF. One-time injections of synthetic YAP5SA mmRNA were not better than STEMIN in cardiac repair. In fact, YAP5SA mmRNA injection did not improve EF over STEMIN mmRNA.

**Figure 5.**
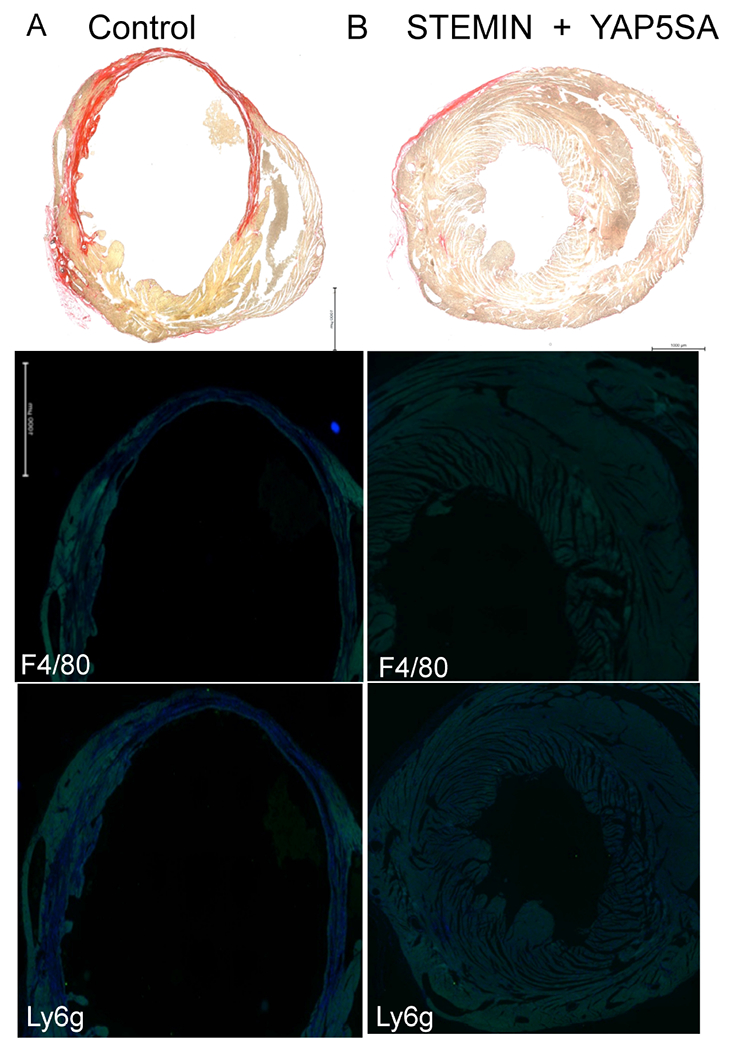
Hearts injected with a combination of STEMIN and YAP5SA revealed little if any staining for immune cells infiltrates. (A) Anti-F4/80 antibody recognizes the mouse F4/80 antigen, a 160 kD glycoprotein expressed by murine macrophages^[20]^. The secondary antibody (goat anti-rat Alexa Fluor 488) showed staining overlapped the infarcted ventricular wall of the control hearts. Anti-Ly6g stained a marker expressed predominantly on neutrophils^[21]^; in addition, a subset of eosinophils, differentiating pre-monocytes, and plasmacytoid dendritic cells showed staining in the infarcted heart. (B) Sectioned STEMIN and YAP5SA synthetic mmRNA treated infarcted hearts after four weeks did not reveal fibrotic scarring or staining with anti-F4/80 or anti-Ly6g.

We also considered whether immune cells infiltrated the infarcted ventricular walls [Figure 5A]. Anti-F4/80 antibody recognizes the mouse F4/80 antigen, a 160 kD glycoprotein expressed by murine macrophages^[22]^. The secondary antibody (goat anti-rat Alexa Fluor 488) showed staining overlapped the infarcted ventricular wall of the control hearts [Figure 5A]. Similarly, anti-Ly6g, which stains a marker expressed predominantly on neutrophils, showed staining in the infarcted heart^[23]^. We observed that hearts injected with a combination of STEMIN and YAP5SA mmRNA showed little, if any, staining for immune cells infiltrates [Figure 5B].

## DISCUSSION

Considerable effort has been invested to develop interventions aimed at gaining functional CM, as well as enhancing the function of surviving CM in diseased hearts. Herein, we describe a combination of factors that effectively induce cardiomyocyte proliferation both *in vitro* and *in vivo*. The serum response factor mutant, STEMIN, blocked cardiac differentiation in culture, yet propelled CM to express stem and cell cycle gene activity. STEMIN shares similar attributes to the YAP mutant, YAP5SA, a HIPPO signaling factor that promotes cell growth. Our strategy was to generate synthetic modified STEMIN and YAP5SA mmRNA for highly efficient transfection into CM. We observed robust expression of nuclear DNA replication fork and replisome pathway genes, the center point of initiating DNA replication. Bioinformatics analysis^[15]^ revealed the upregulation of multiple cell cycle gene clusters with co-expression of STEMIN and YAP5SA, while gene clusters associated with cardiomyocyte differentiation (GO: 0055007), sarcomeric assembly, and cardiac muscle contraction (GO: 0060048) were profoundly downregulated. Synthetic mmRNA encoded for STEMIN and YAP5SA, injected into the left ventricles of infarcted adult mice, promoted an over 17-fold increase in nuclear proliferation, within a day, and ameliorated cardiac dysfunction, 3-4 weeks later. STEMIN and YAP5SA promoted cardiomyocyte proliferation by inhibiting SRF-dependent cardiomyocyte differentiation, thus pushing CM into a more primitive stage to foster cell replication.

Cardiac fibrosis observed in the infarcted mice was virtually cured by STEMIN and YAP5SA mmRNA therapy. Circulating monocytes have an important role in penetrating damaged tissue and differentiate into macrophages with pro-inflammatory properties or the M1 phenotype^[22]^. Later, activated M2-type macrophages induce the resolution of inflammation and tissue regeneration. We observed a reduced infarct zone in the combination mmRNA injected group compared to control with little fibrosis [Figure 4C]. Immune cells infiltrated the infarcted ventricular walls, as shown by anti-F4/80 antibody [Figure 5]. Anti-Ly6g stained neutrophils, pre-monocytes, and plasmacytoid dendritic cells in the infarcted heart^[22,23]^. We observed that hearts injected with a combination of STEMIN and YAP5SA mmRNA revealed little if any staining for immune cell infiltrates [Figure 5]. ATACseq revealed enhanced chromatin activity of ANGPTL genes, including ANGPTL4^[15]^. Highly induced angiopoietin-like 4 (ANGPTL4) suppressed the activation of inflammatory macrophages. ANGPTL4, a secreted angiopoietin-like glycoprotein, was shown to be effective in mouse models of MI^[24]^.

Synthetic mRNA may be used as a safe and efficient gene delivery vehicle in adult hearts. Compared to viral vectors, the transient gene expression that mmRNA provides is far more controllable, which makes the mmRNA gene-delivery method a safer option to deliver therapeutic factors for cardiac regeneration. In fact, adenovirus delivery of stem cell factors is initially curative for regenerating cardiac function, but it causes cardiac rhadomyosarcomas in the long term^[25]^. Given the post-transcription nature of mRNA, mmRNA does not require transfer to the nucleus to get the expression of the target protein. Besides, mmRNA-based gene delivery can deliver gene combinations with different ratios specifically tailored to patients with a different course of the disease. Our data suggest that synthetic mRNA may be used to deliver STEMIN and YAP5SA into adult CM both *in vitro* and *in vivo* to achieve high transfection efficiency with little biosafety concern. Recently, an adeno-associated virus 9 (AAV9)-based gene therapy was shown to locally knock down the Hippo pathway gene Salvador (Sav) in border zone CM in a pig model of ischemia/reperfusioninduced MI^[26]^. Three months after injection, pig hearts treated with a high dose of AAV9-Sav-shRNA exhibited small improvements (4%-8% upon viral dosage) in EF. Thus, enabling YAP signaling is beneficial in the short term. However, the inability to shut off AAV expression over three months may keep the infected CM in a perpetual circular mode of downregulating cardiac differentiation genes. Inducing tissue regeneration by short-term treatments with STEMIN and YAP5SA mRNA may become a useful and safer strategy to treat debilitating human cardiac disease.

## Supplementary Material

Supplementary Material

## Data Availability

Echocardiography primary data is shown in Supplementary Materials.
