# Supplementary material for: STEMIN and YAP5SA synthetic modified mRNAs regenerate and repair infarcted mouse hearts
Source: J Cardiovasc Aging. Author manuscript; Available in PMC 2022 Jul 25. (PMC9311335; doi:10.20517/jca.2022.20)
Supplement: Supplementary Material [file NIHMS1816959-supplement-Supplementary_Material.pdf]

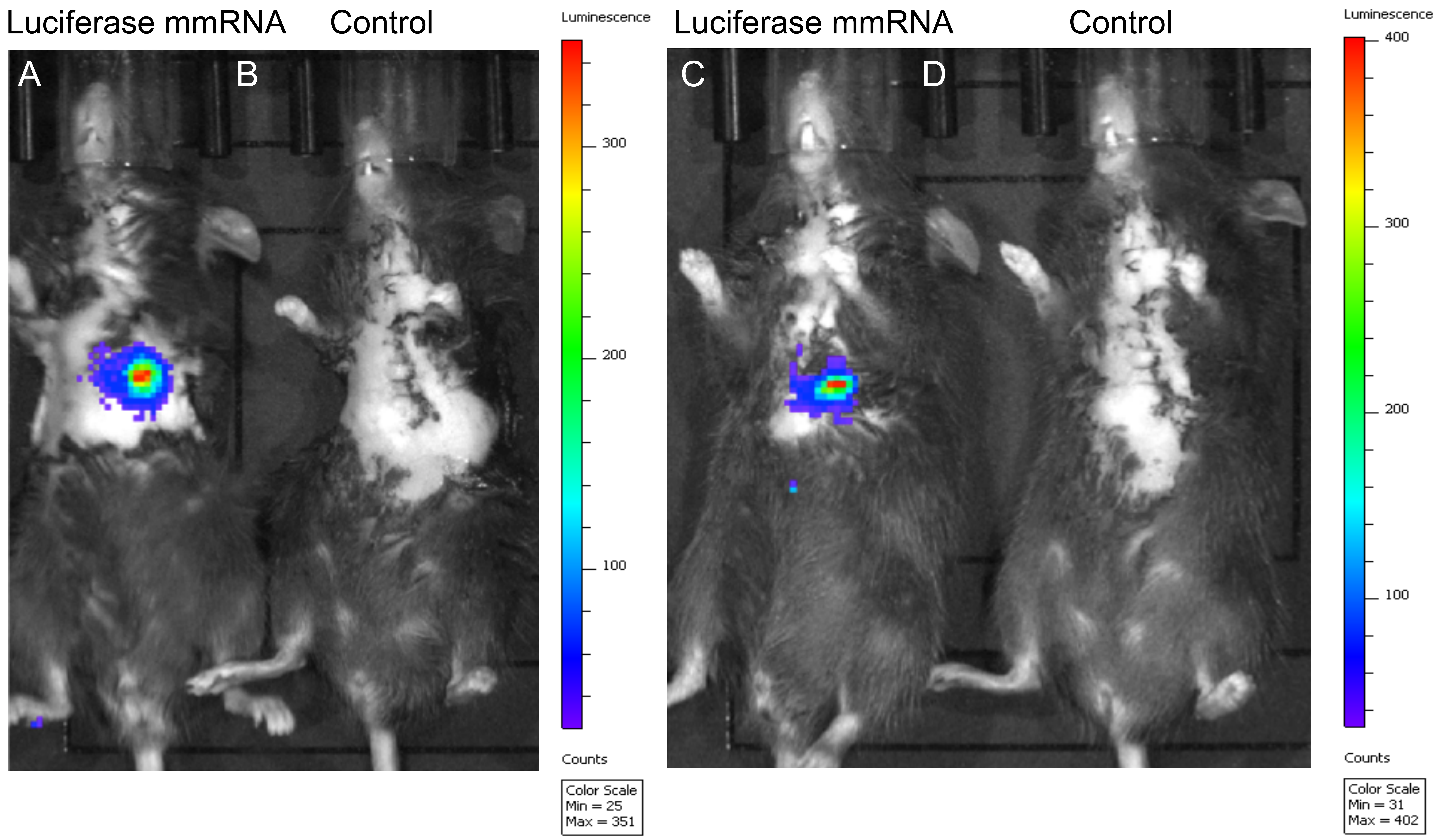

Xiao et al. Supplemental Figure 1

Slices	Intergrated density
1	26.682
2	22.298
3	29.197
4	65.424
5	39.222
6	28.816
7	28.702
8	30.836
9	35.791
10	87.439
11	42.424
12	32.549
13	29.54
14	29.845
15	27.787
16	24.471
17	23.099
18	20.24
19	17.888
20	14.141
21	32.399
22	62.854
23	56.756
24	42.538
25	8.081
26	7.052
27	5.922
28	5.908
29	2.325
30	3.202

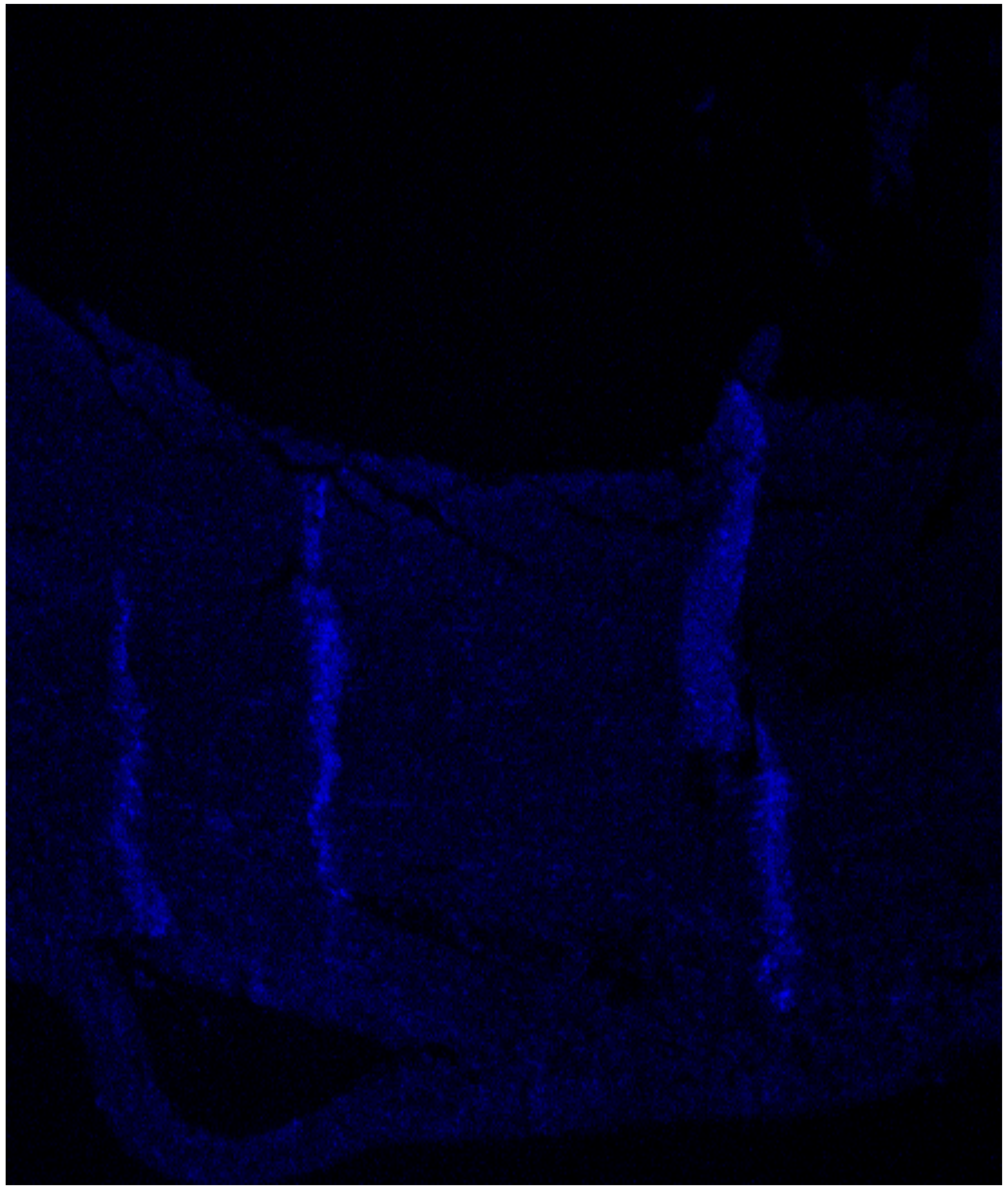

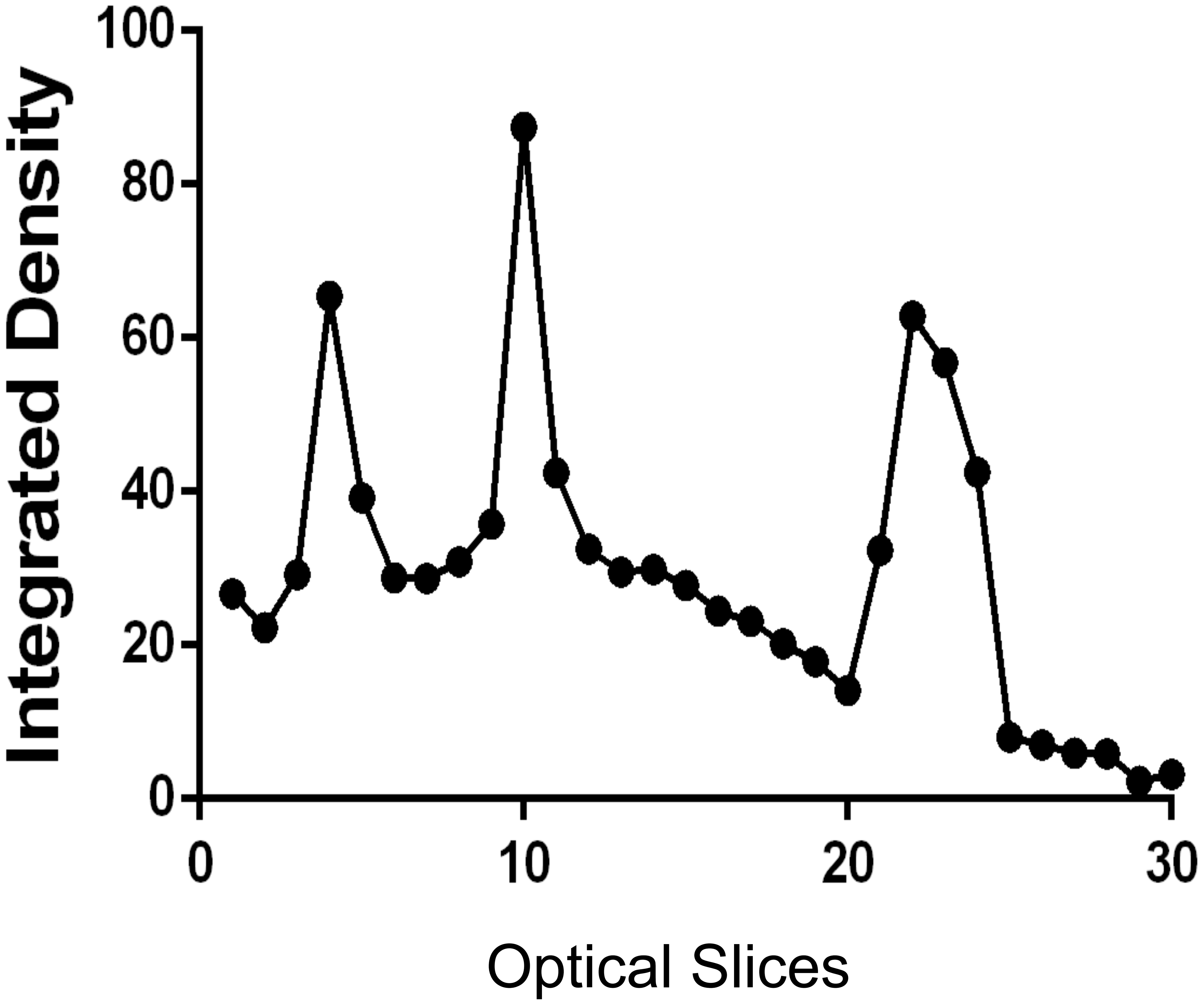

Xiao et al., Supplemental Figure 2

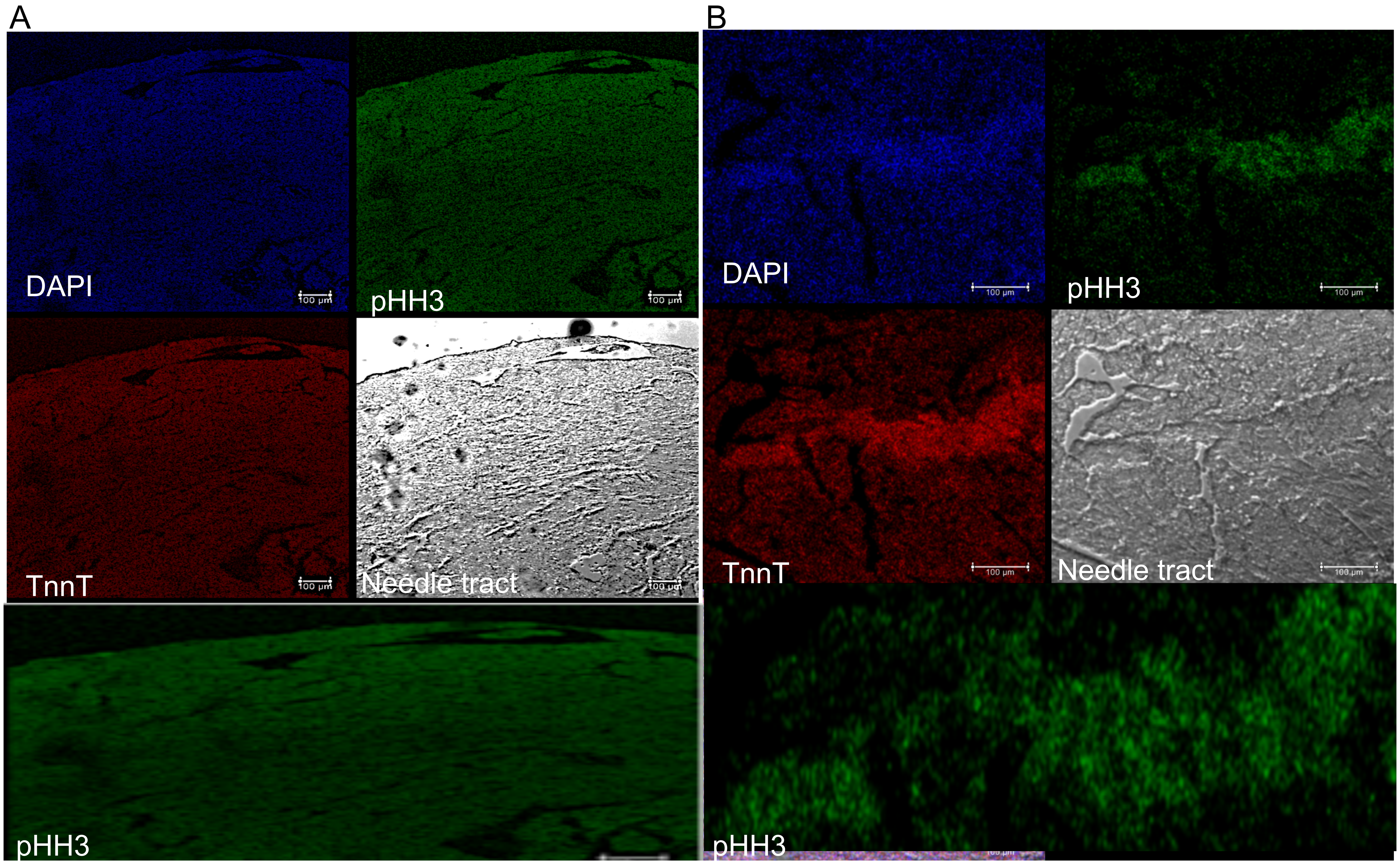

Xiao et al., Supplemental Figure 3

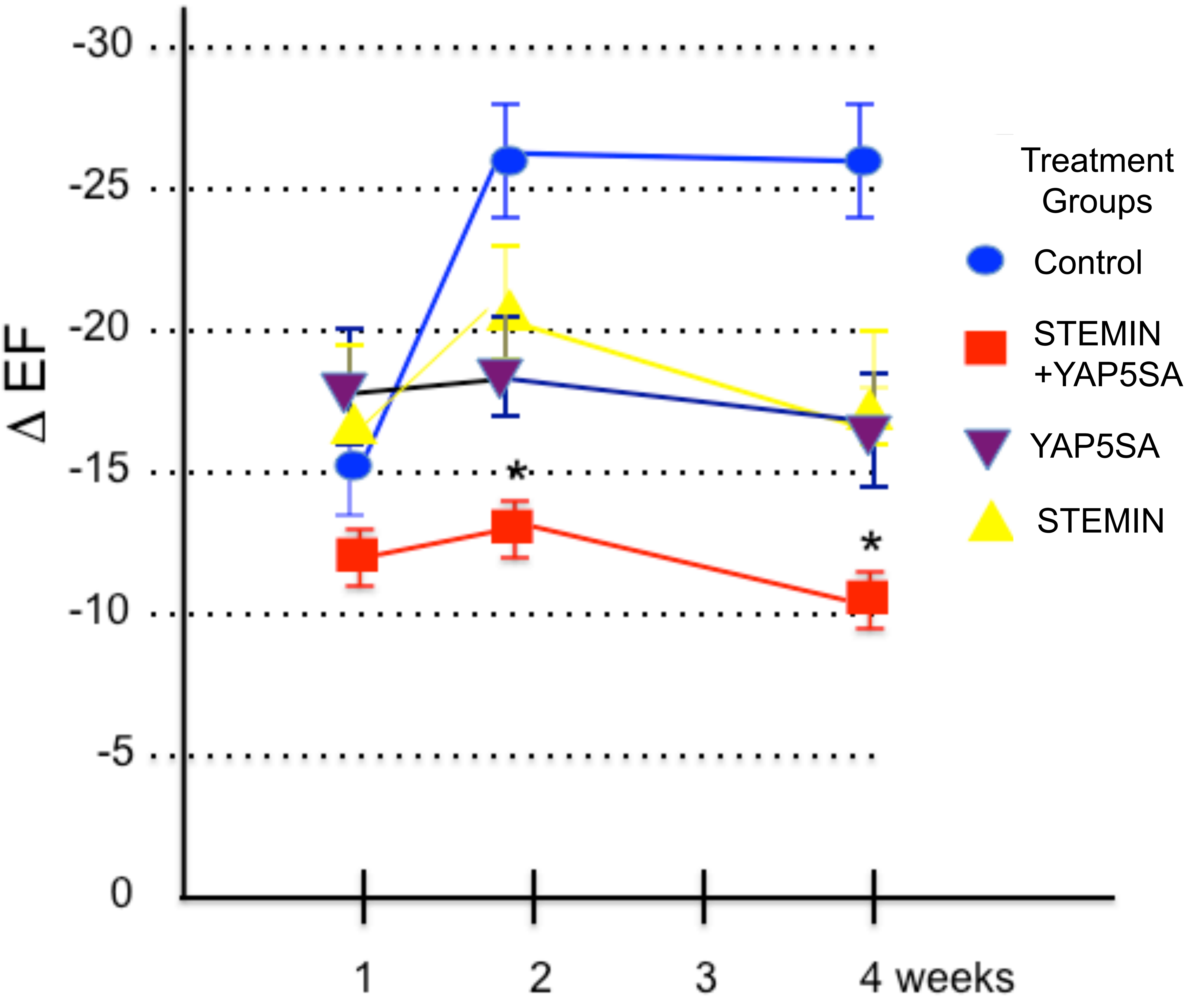

Supplemental Figure 4, Xiao et al

Before MI

P852 CONTROL

Ejection Fraction	%	47.408607
Fractional Shortening	%	16.055545
Cardiac Output	mL/min	15.091782

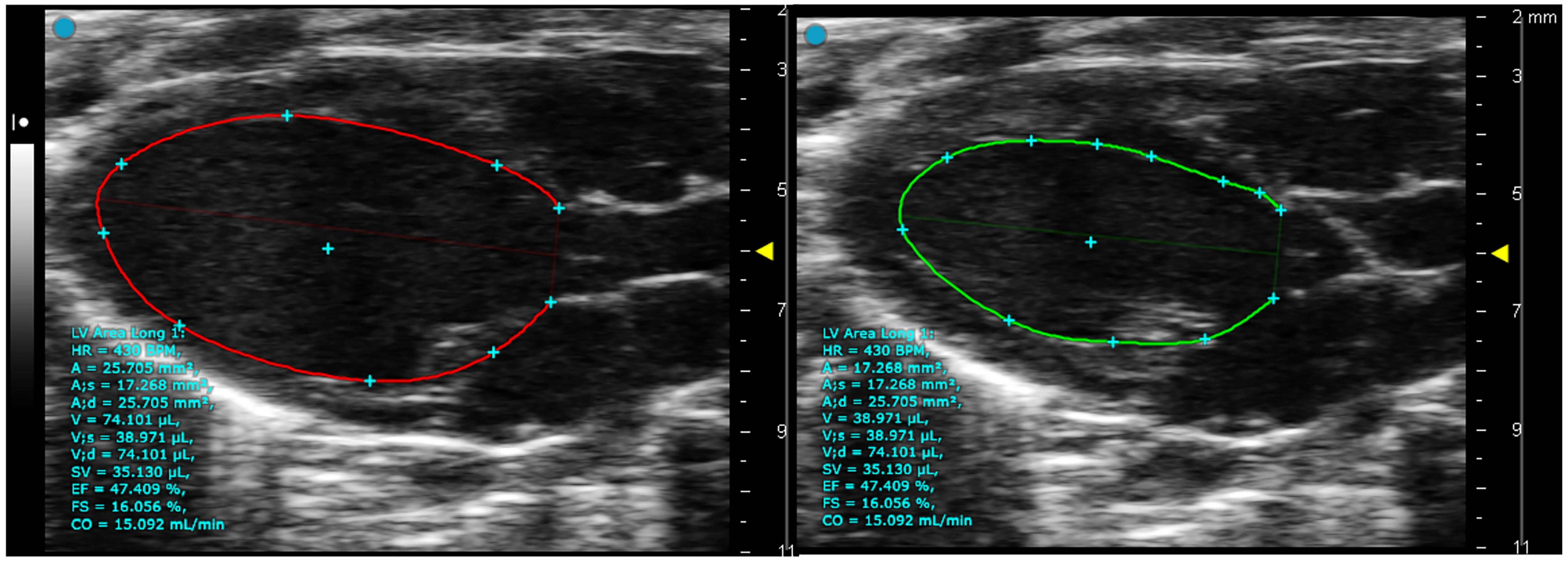

2w MI

P852 CONTROL

Ejection Fraction	%	22.46891
Fractional Shortening	%	11.003255
Cardiac Output	mL/min	16.362695

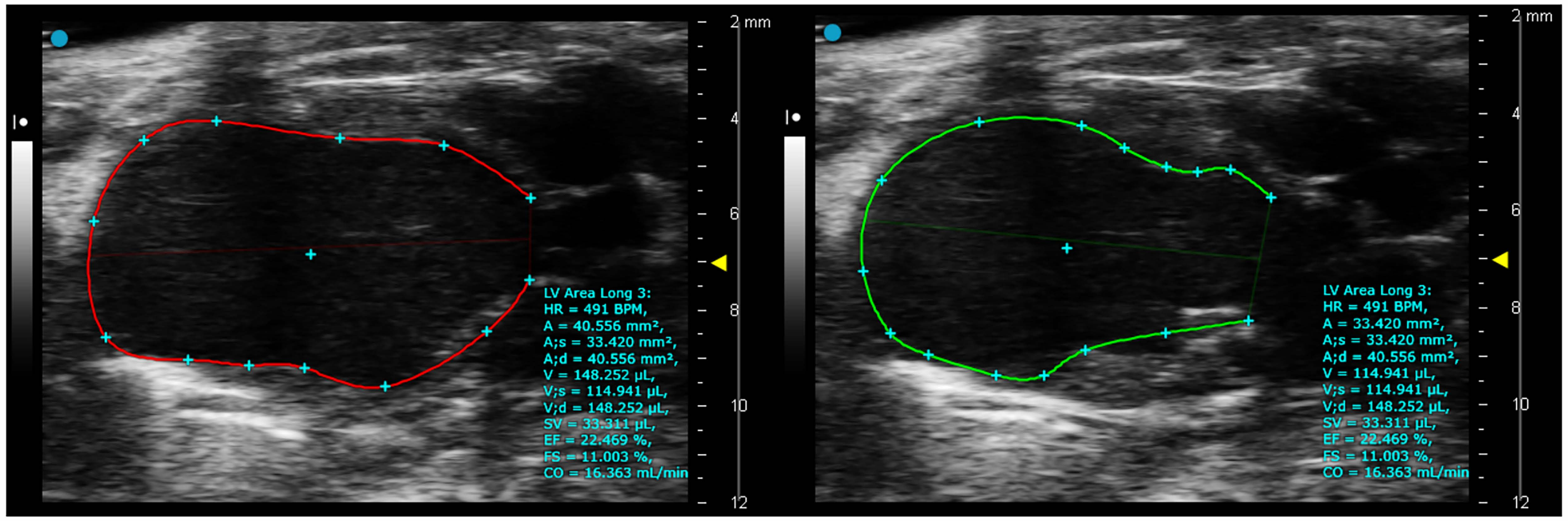

4 w MI

P852 CONTROL

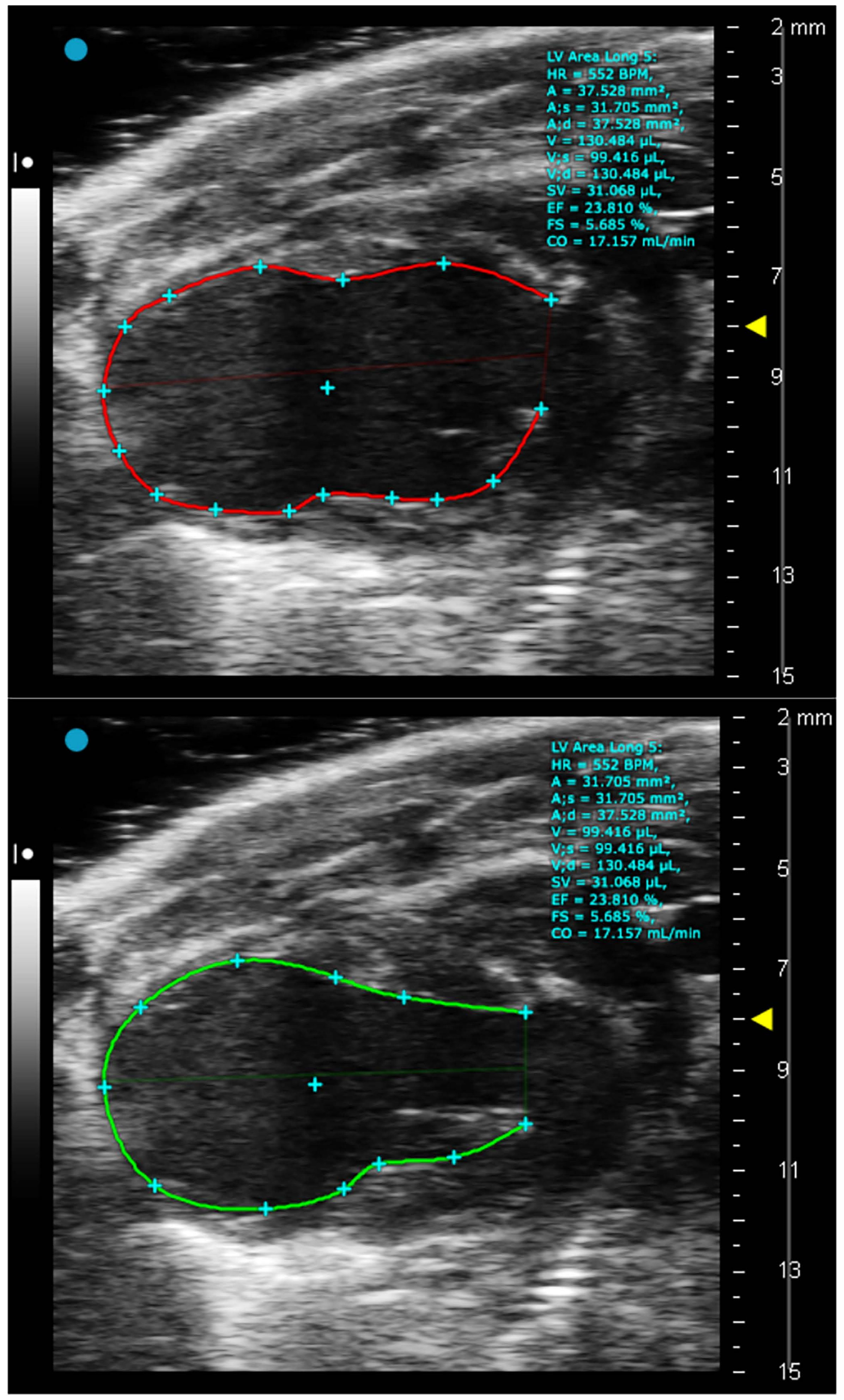

Ejection Fraction	%	23.809781
Fractional Shortening	%	5.684858
Cardiac Output	mL/min	17.157348

1w MI
P855 CONTROL

Ejection Fraction	%	38.680318
Fractional Shortening	%	10.142476
Cardiac Output	mL/min	31.877959

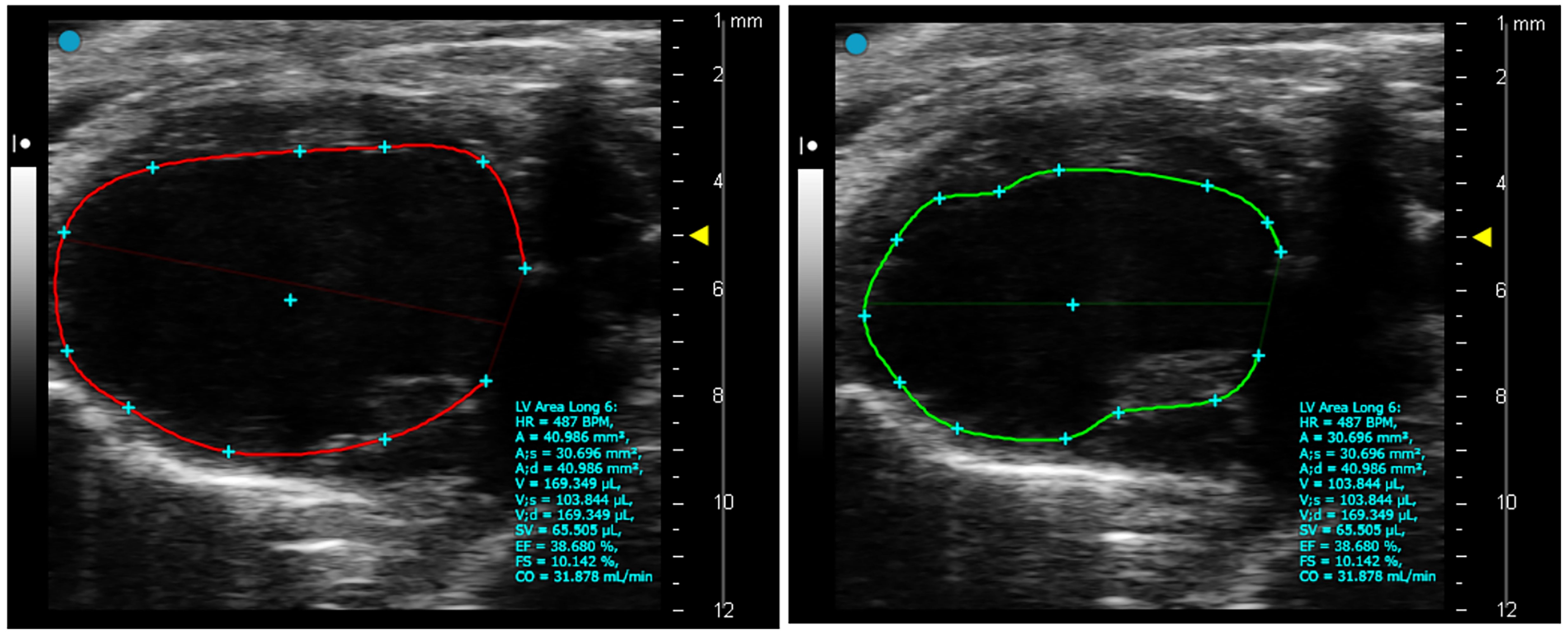

2w MI
P855 CONTROL

Ejection Fraction	%	29.660661
Fractional Shortening	%	3.703728
Cardiac Output	mL/min	18.057436

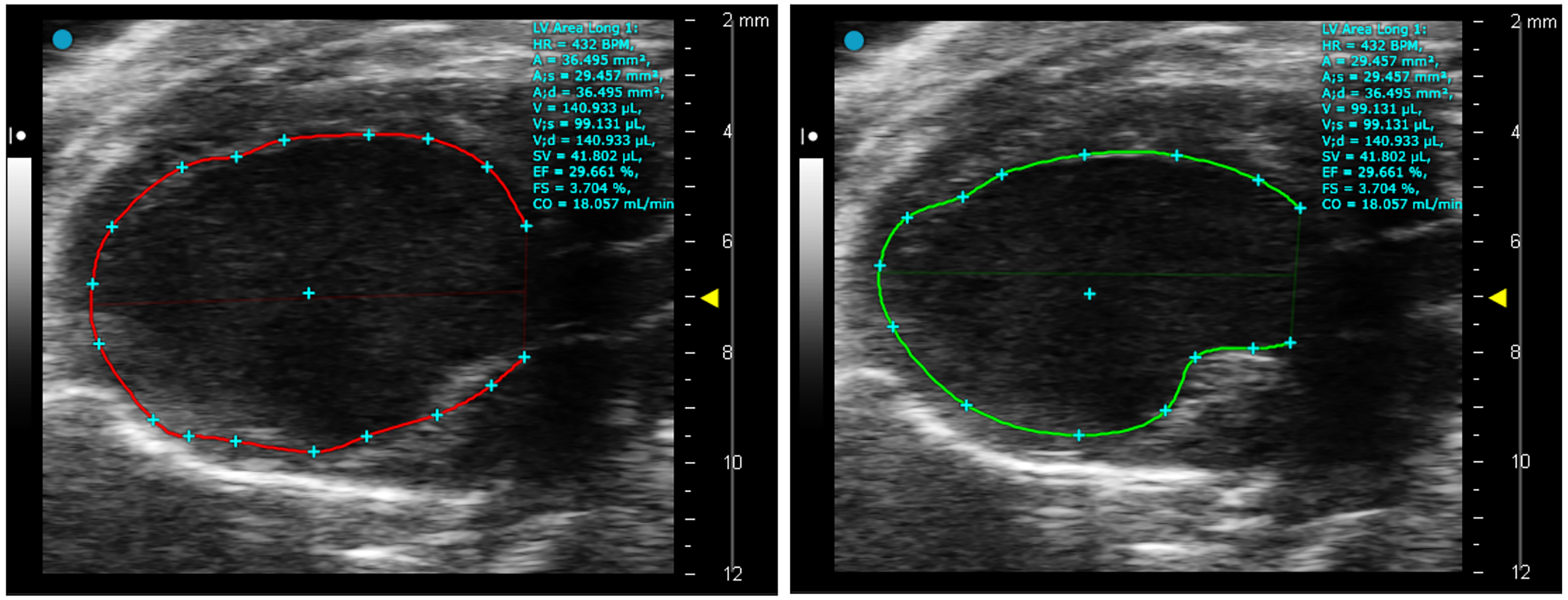

4w MI
P855 CONTROL

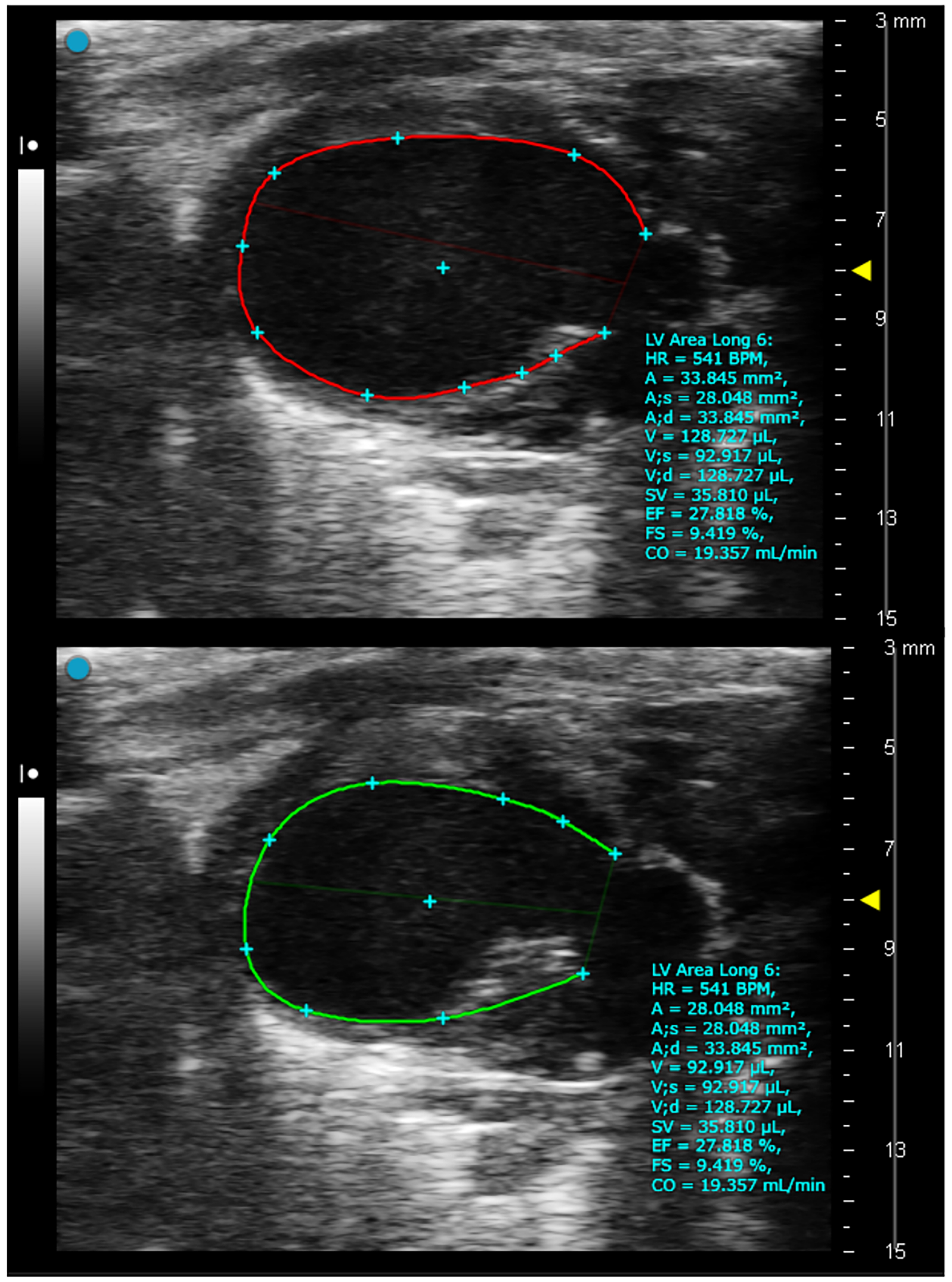

Ejection Fraction	%	27.818233
Fractional Shortening	%	9.419051
Cardiac Output	mL/min	19.3565

Before MI
Q745 CONTROL

Ejection Fraction	%	45.885295
Fractional Shortening	%	13.467596
Cardiac Output	mL/min	19.740986

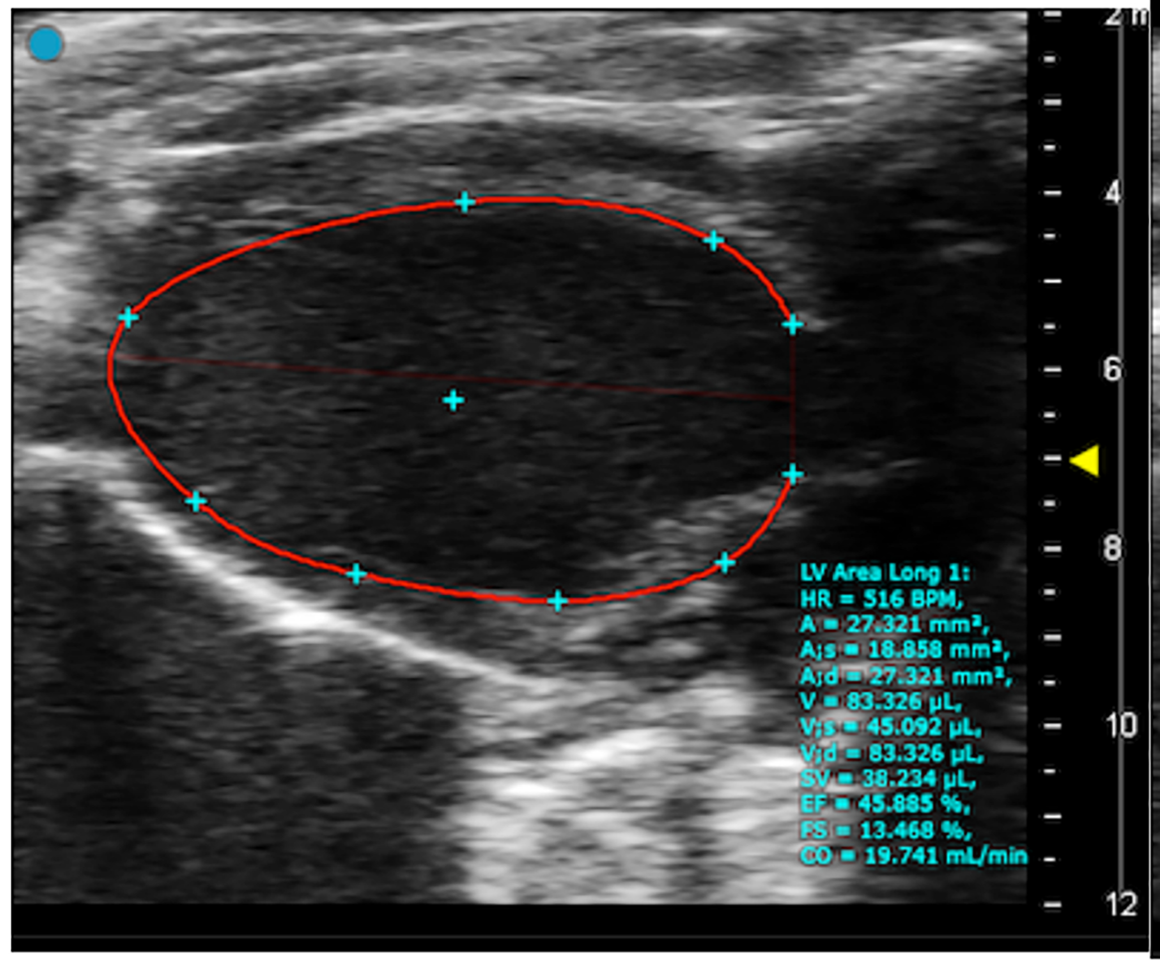

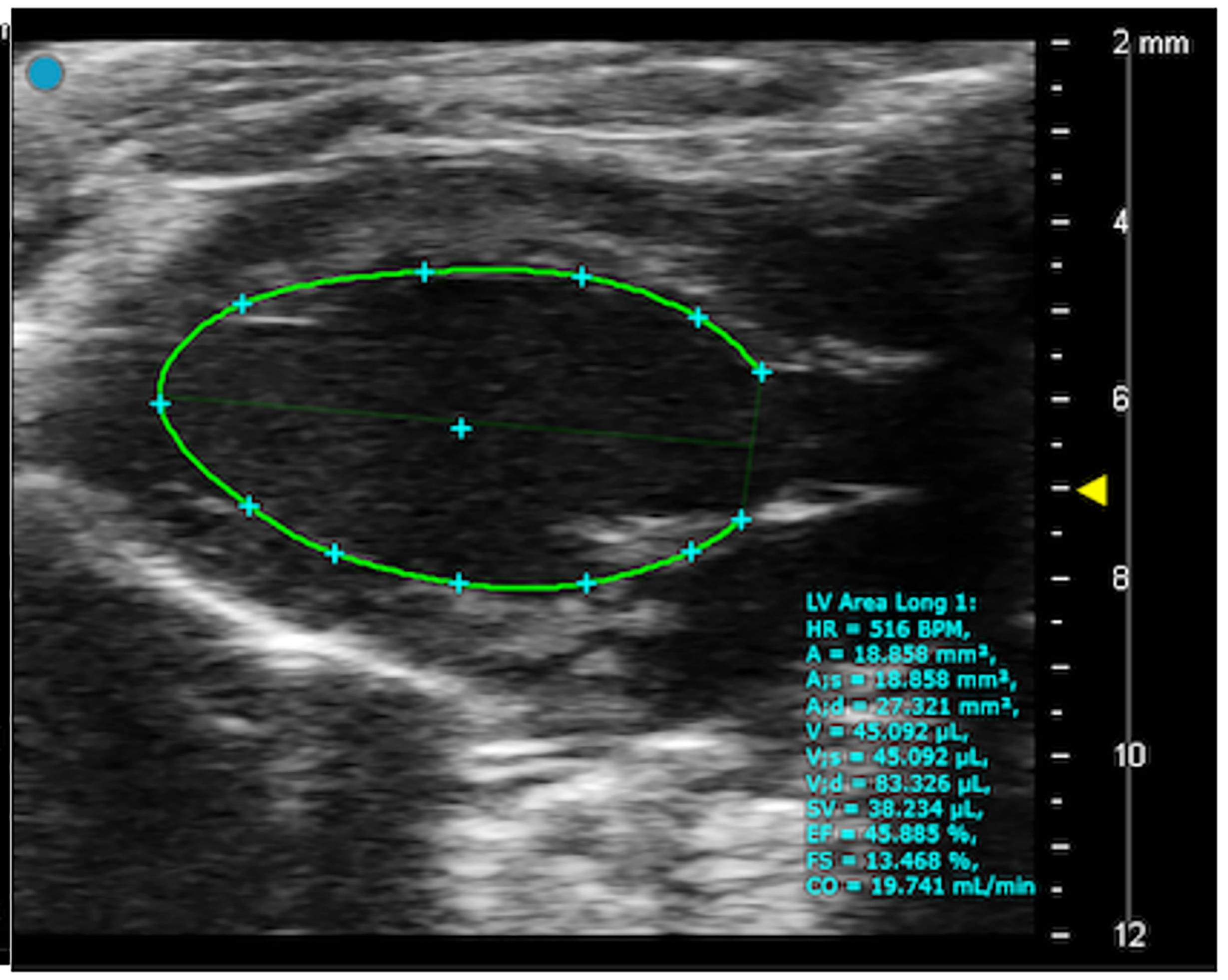

2 week after MI
Q745 CONTROL

Ejection Fraction	%	22.205413
Fractional Shortening	%	8.881372
Cardiac Output	mL/min	18.149669

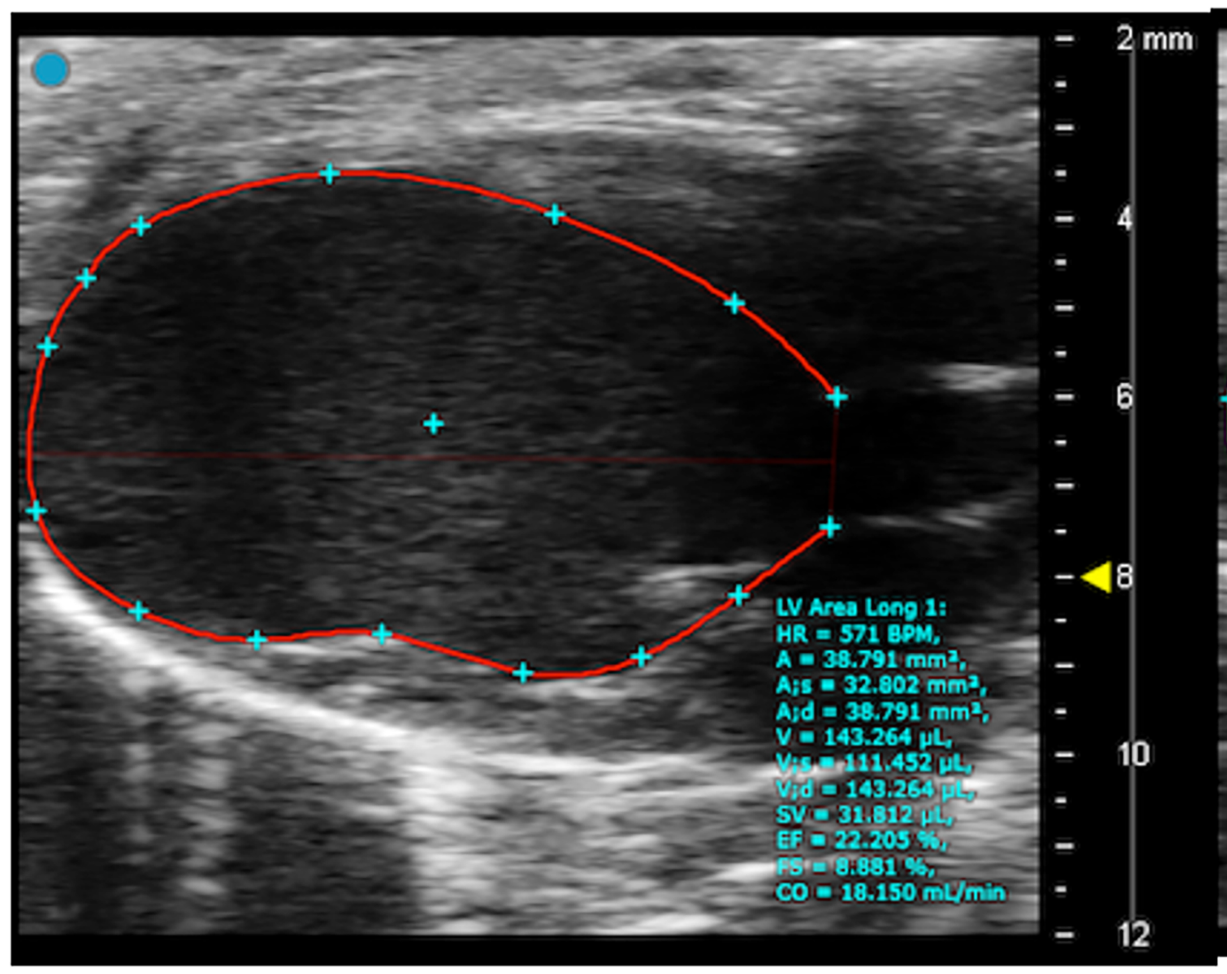

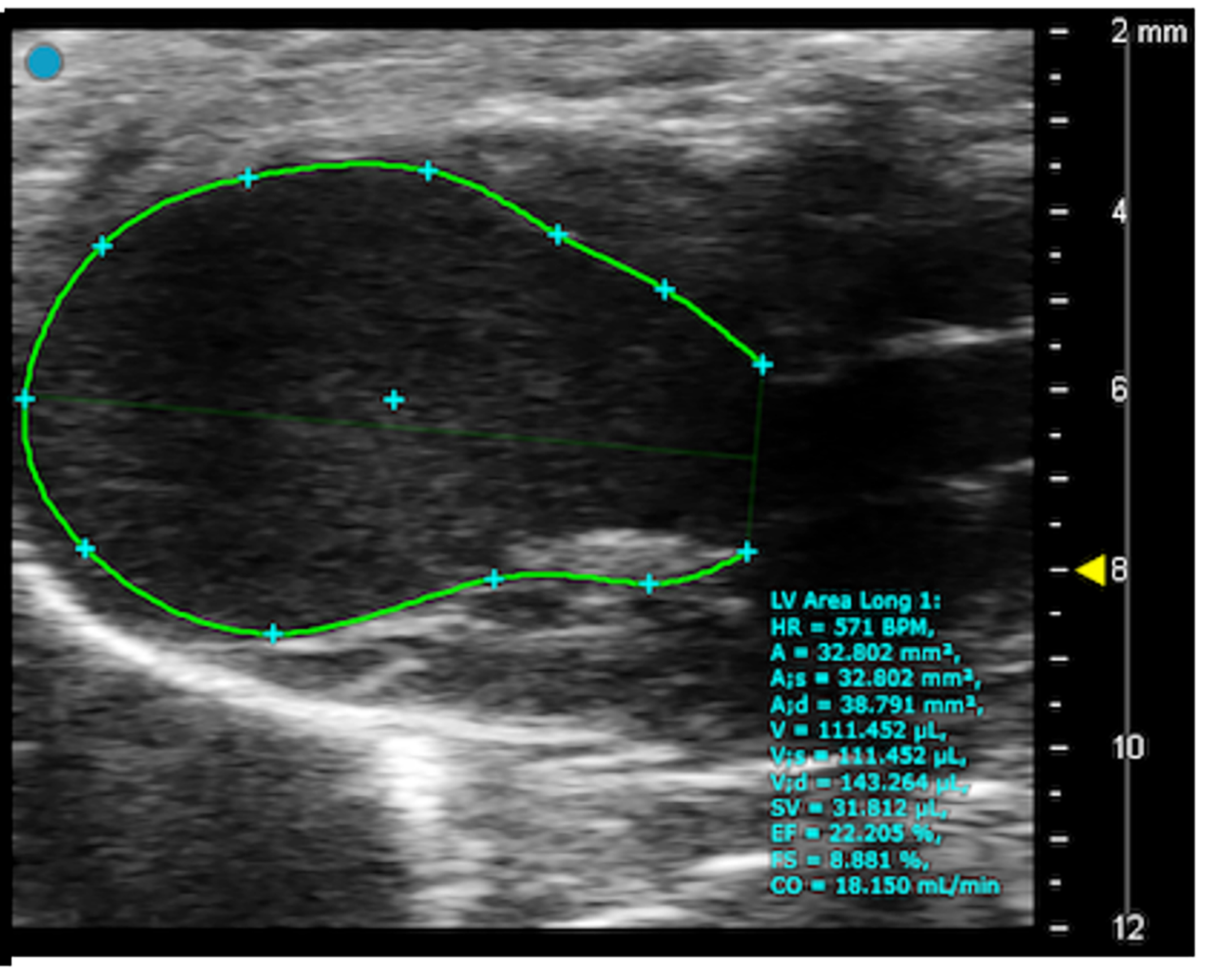

4 week after MI
Q745 CONTROL

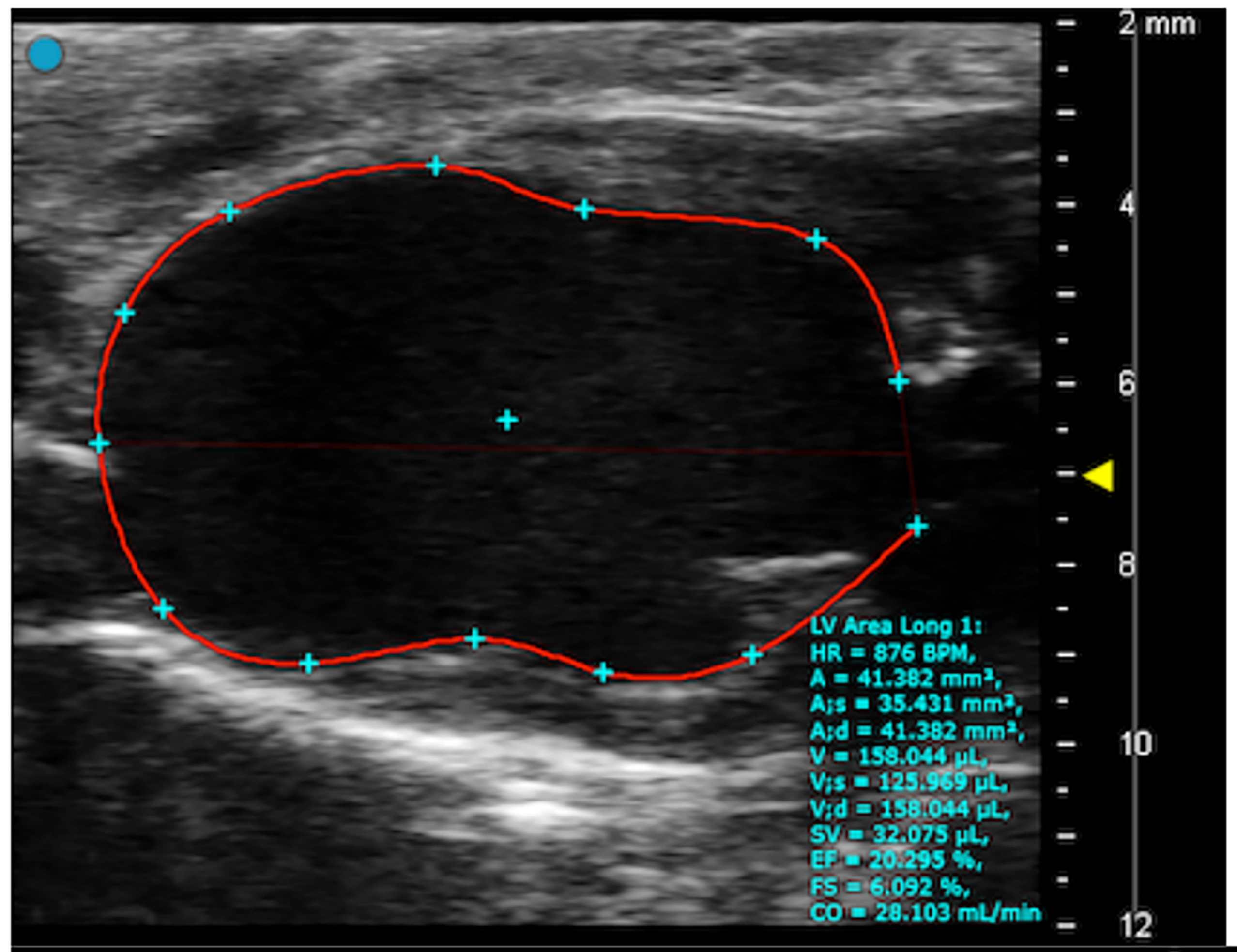

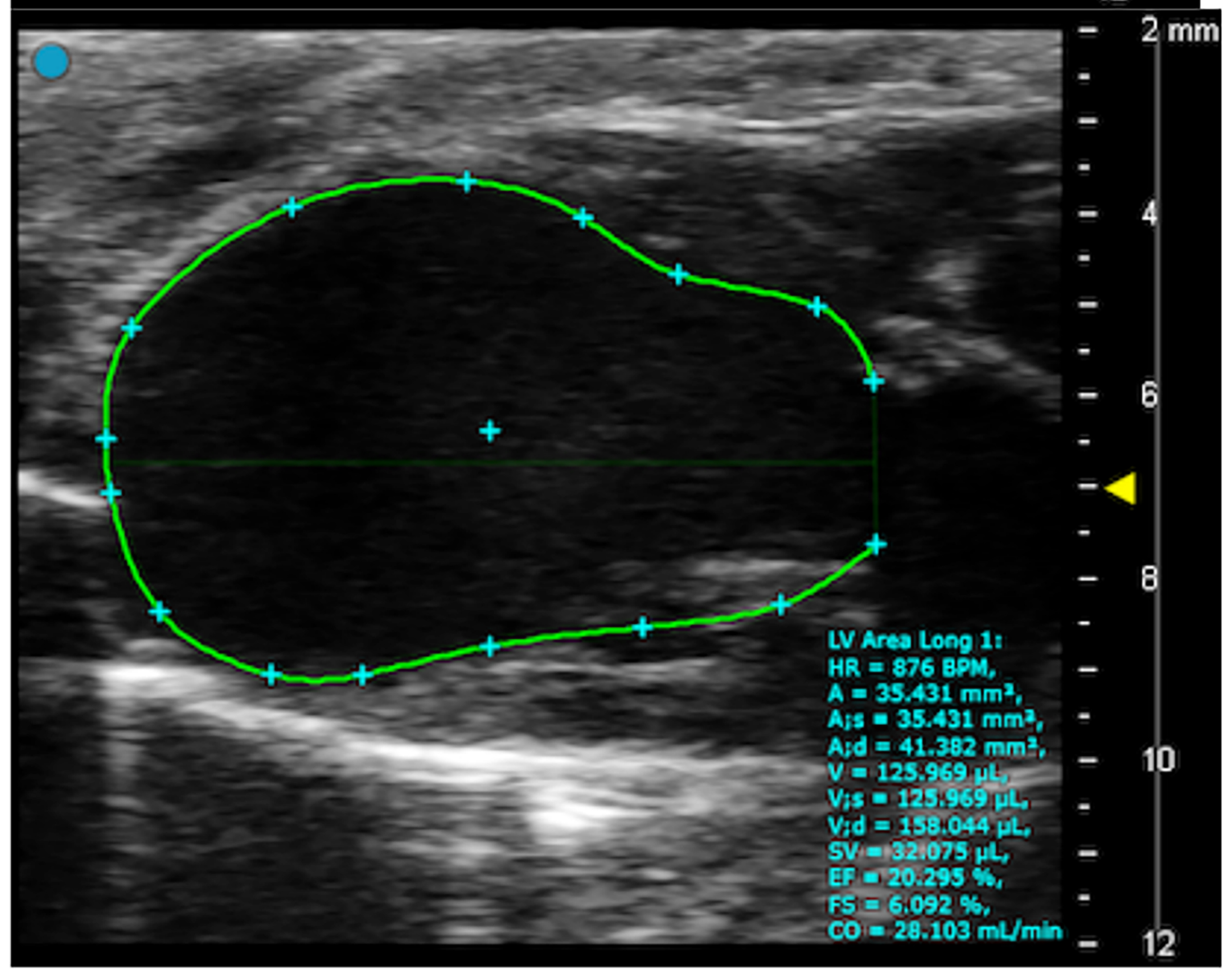

Ejection Fraction	%	20.29497
Fractional Shortening	%	6.092344
Cardiac Output	mL/min	28.103385

Before MI
Q749 CONTROL

Ejection Fraction	%	51.308966
Fractional Shortening	%	10.685558
Cardiac Output	mL/min	21.575102

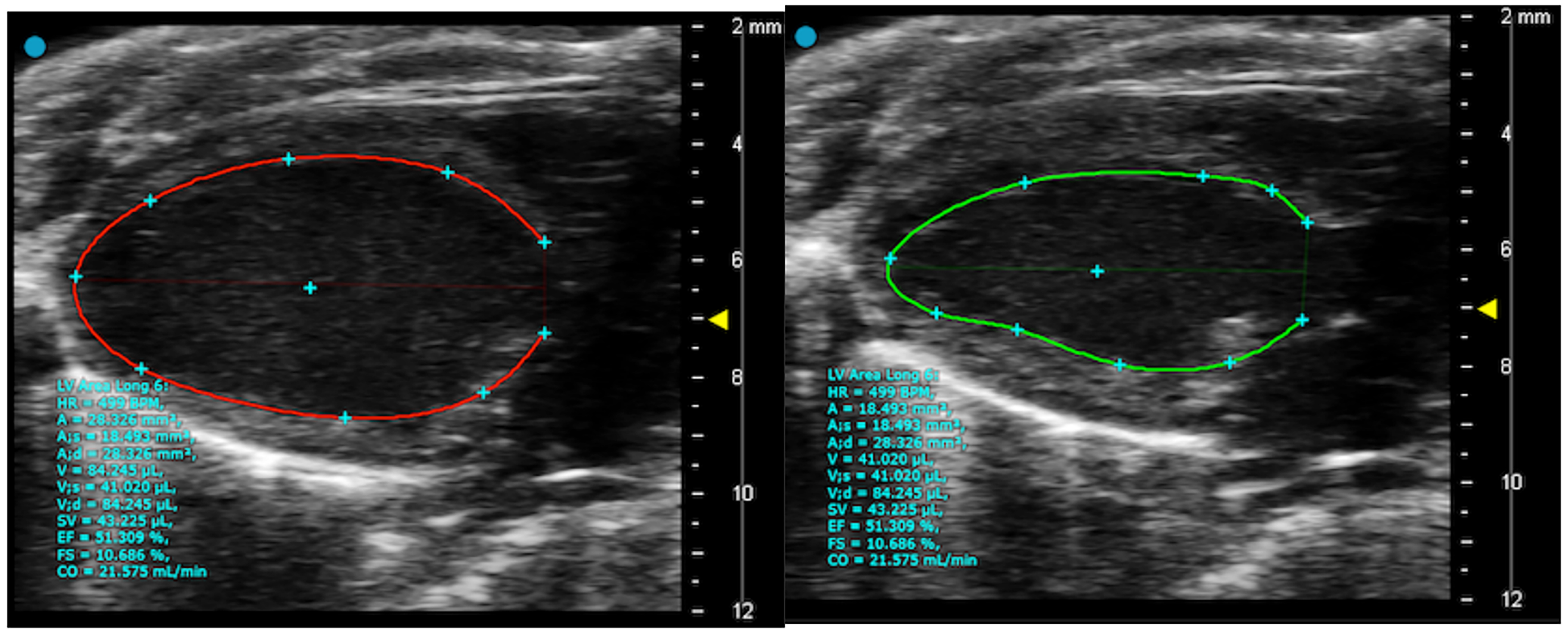

2 week after MI
Q749 CONTROL

Ejection Fraction	%	22.873525
Fractional Shortening	%	7.889406
Cardiac Output	mL/min	18.881535

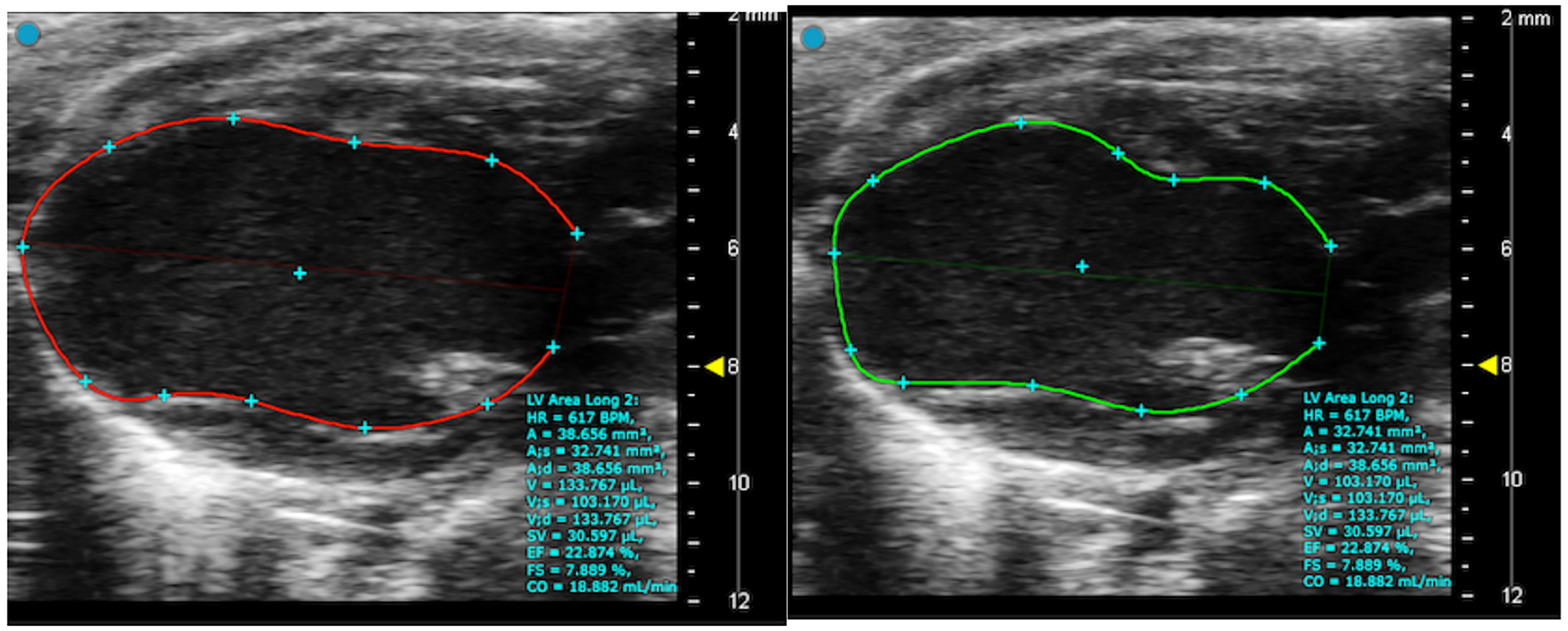

4 week after MI
Q749 CONTROL

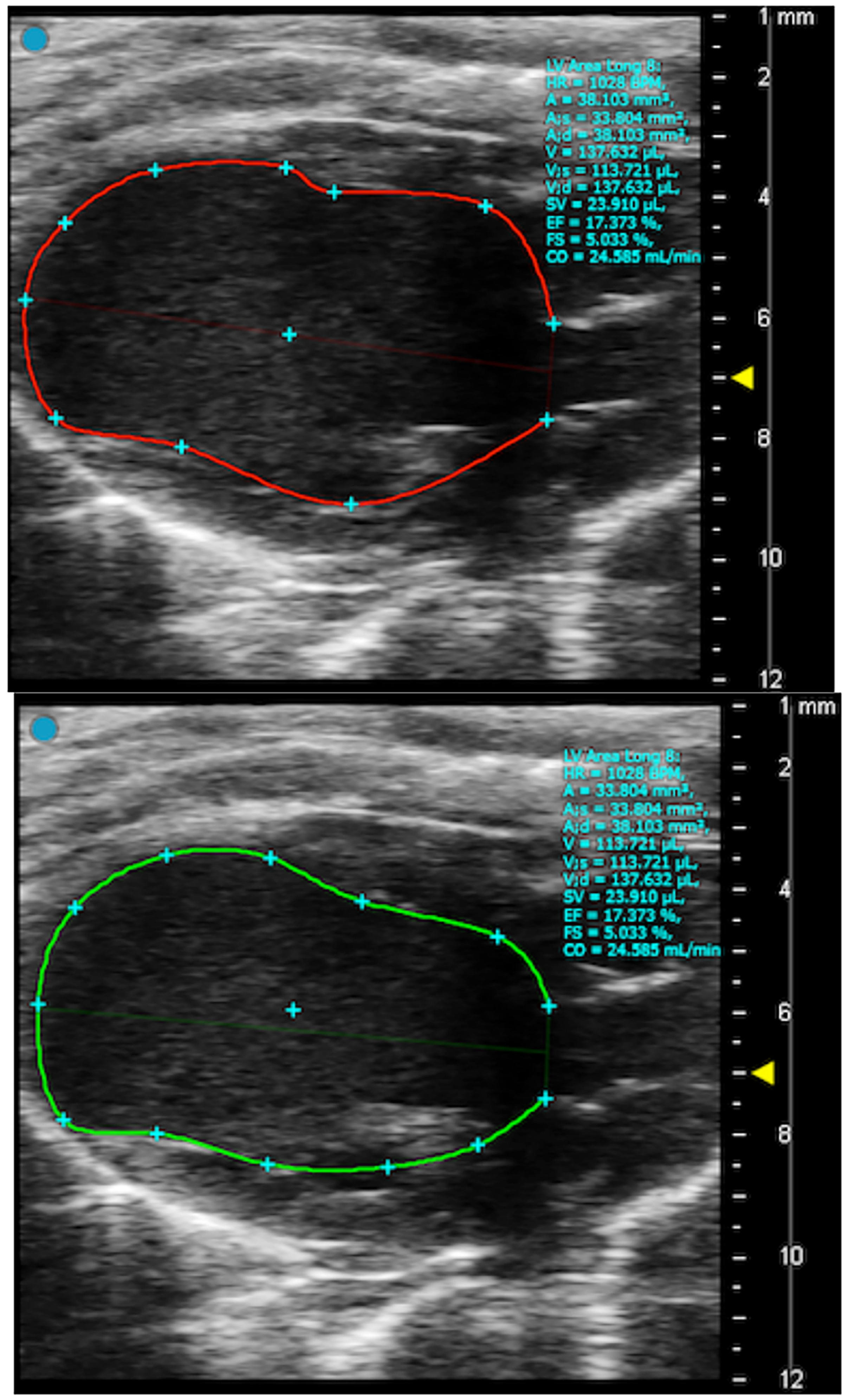

Ejection Fraction	%	17.372772
Fractional Shortening	%	5.032949
Cardiac Output	mL/min	24.584788

Before MI
O316 CONTROL

Ejection Fraction	%	60.82039
Fractional Shortening	%	16.17004
Cardiac Output	mL/min	18.085

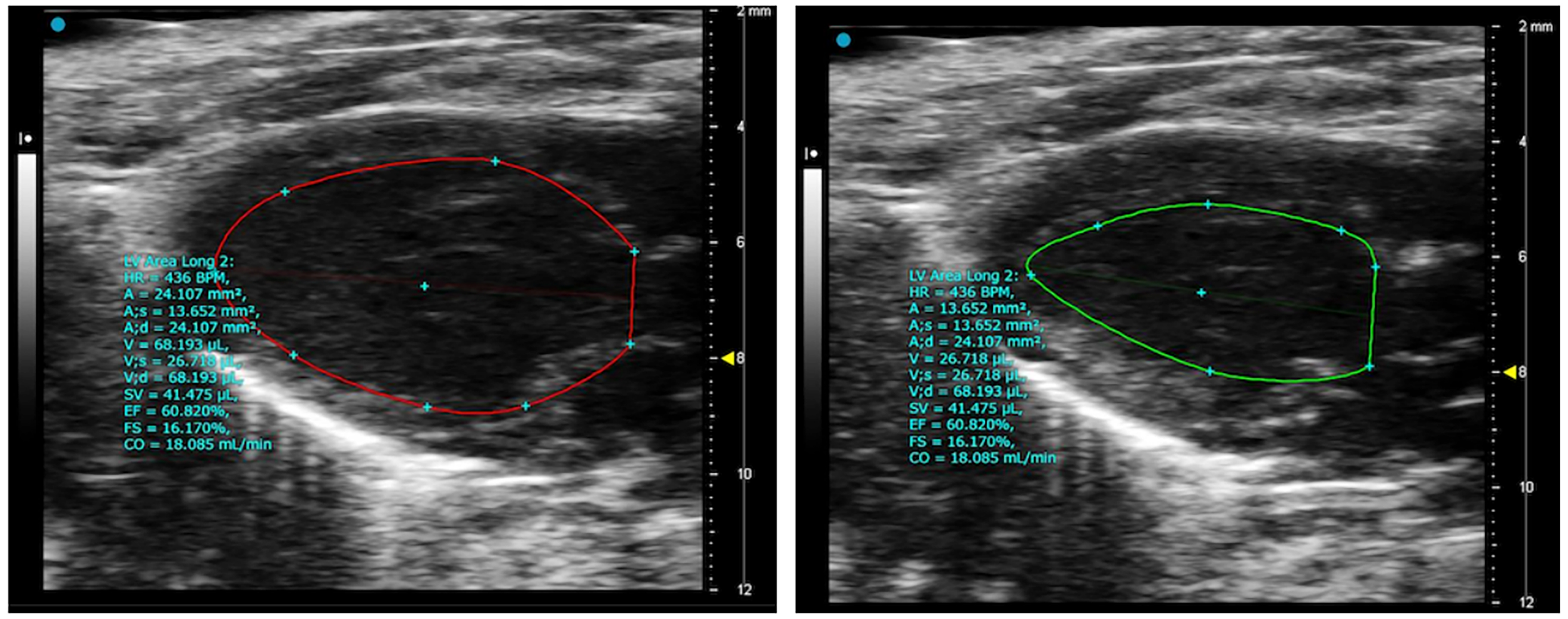

1w MI
O316 CONTROL

Ejection Fraction	%	56.56167
Fractional Shortening	%	15.47886
Cardiac Output	mL/min	22.159

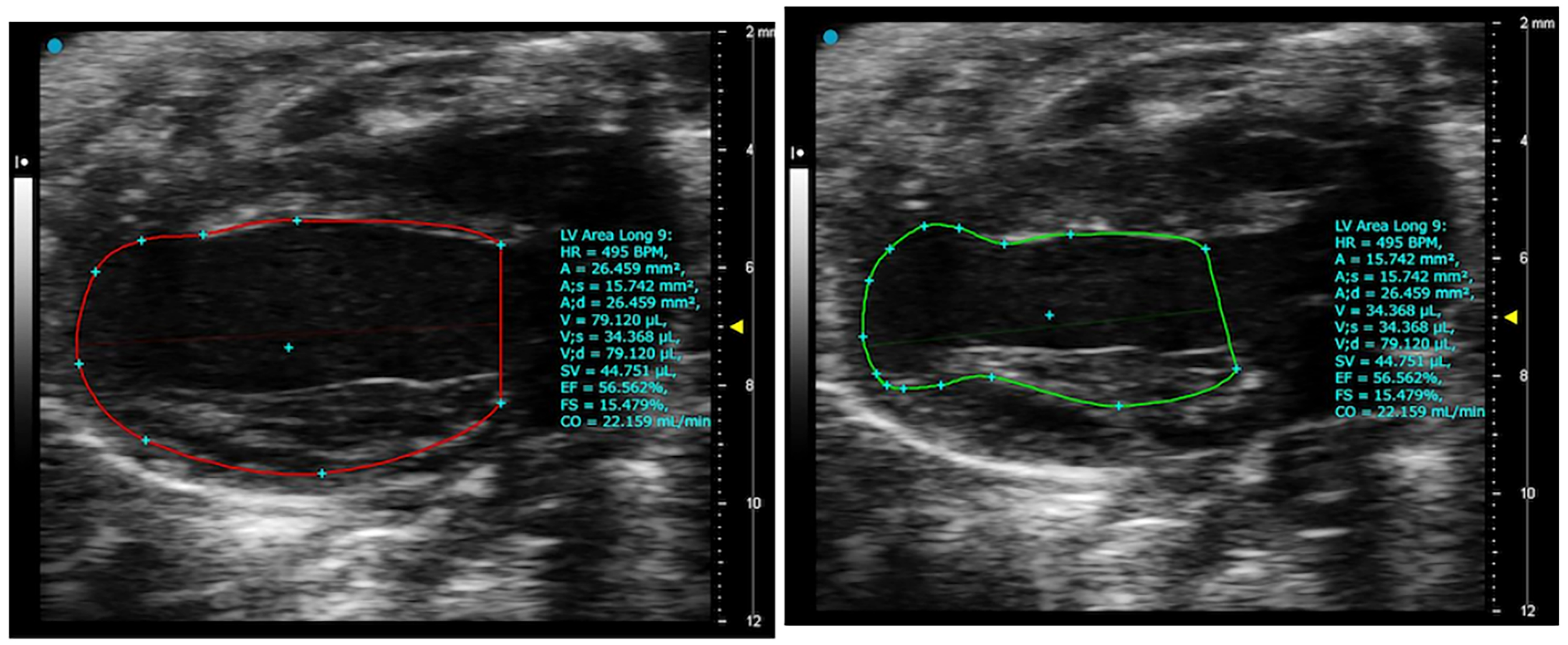

2w MI
O316 CONTROL

Ejection Fraction	%	34.50933
Fractional Shortening	%	0.270355
Cardiac Output	mL/min	9.390

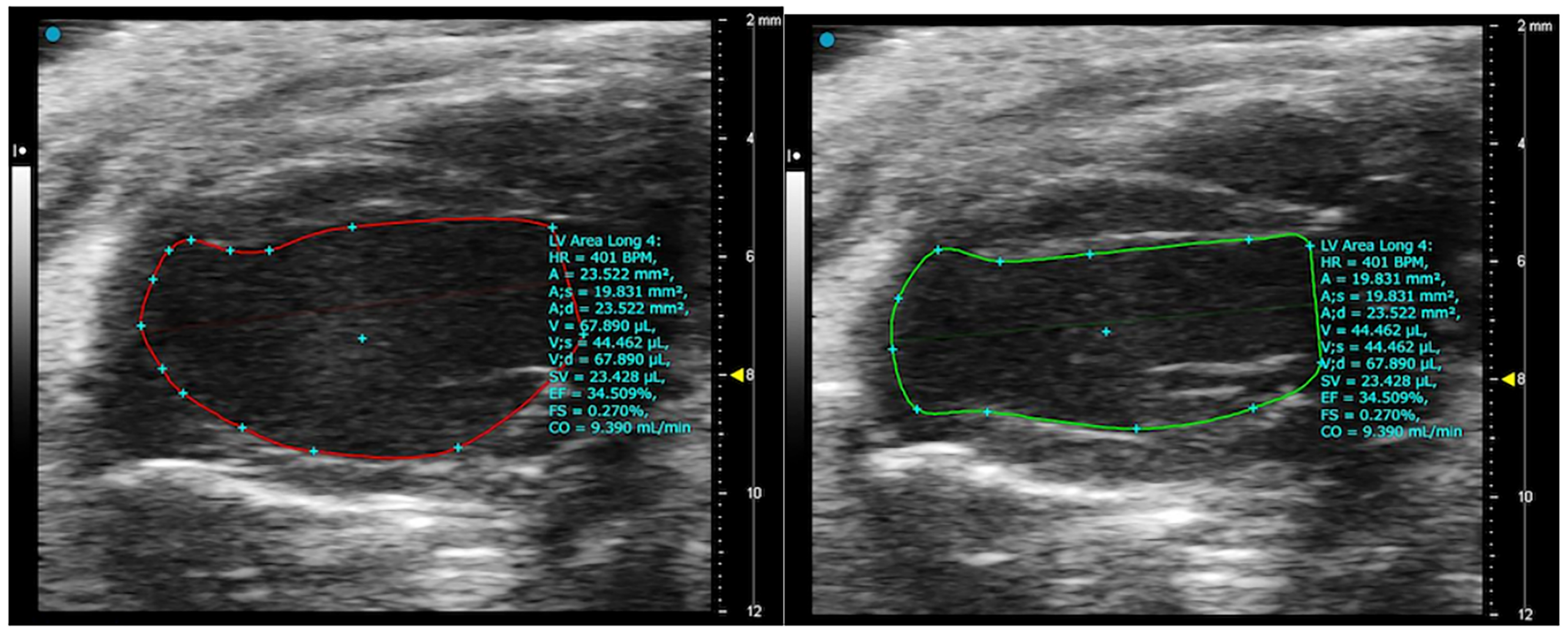

4w MI
O316 CONTROL

Ejection Fraction	%	38.93352
Fractional Shortening	%	9.631073
Cardiac Output	mL/min	12.721

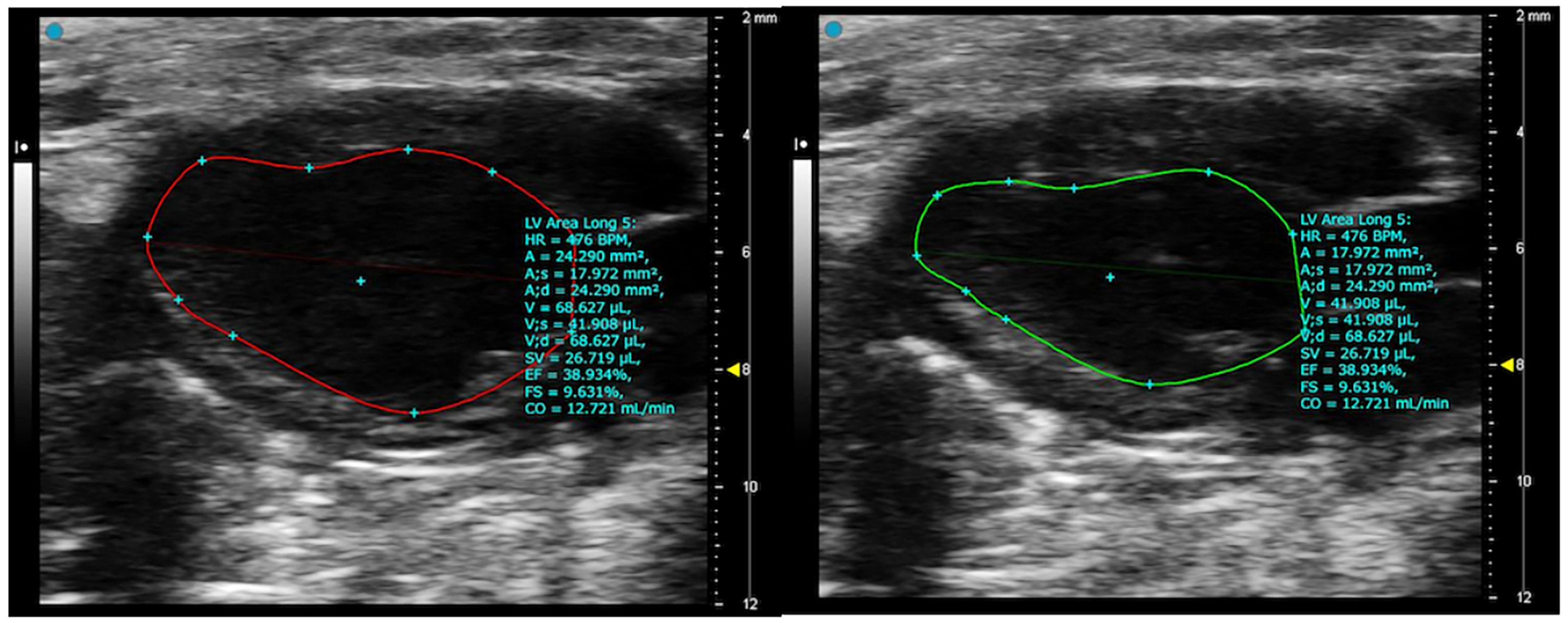

Before MI
O319 CONTROL

Ejection Fraction	%	64.73738
Fractional Shortening	%	26.53226
Cardiac Output	mL/min	23.613

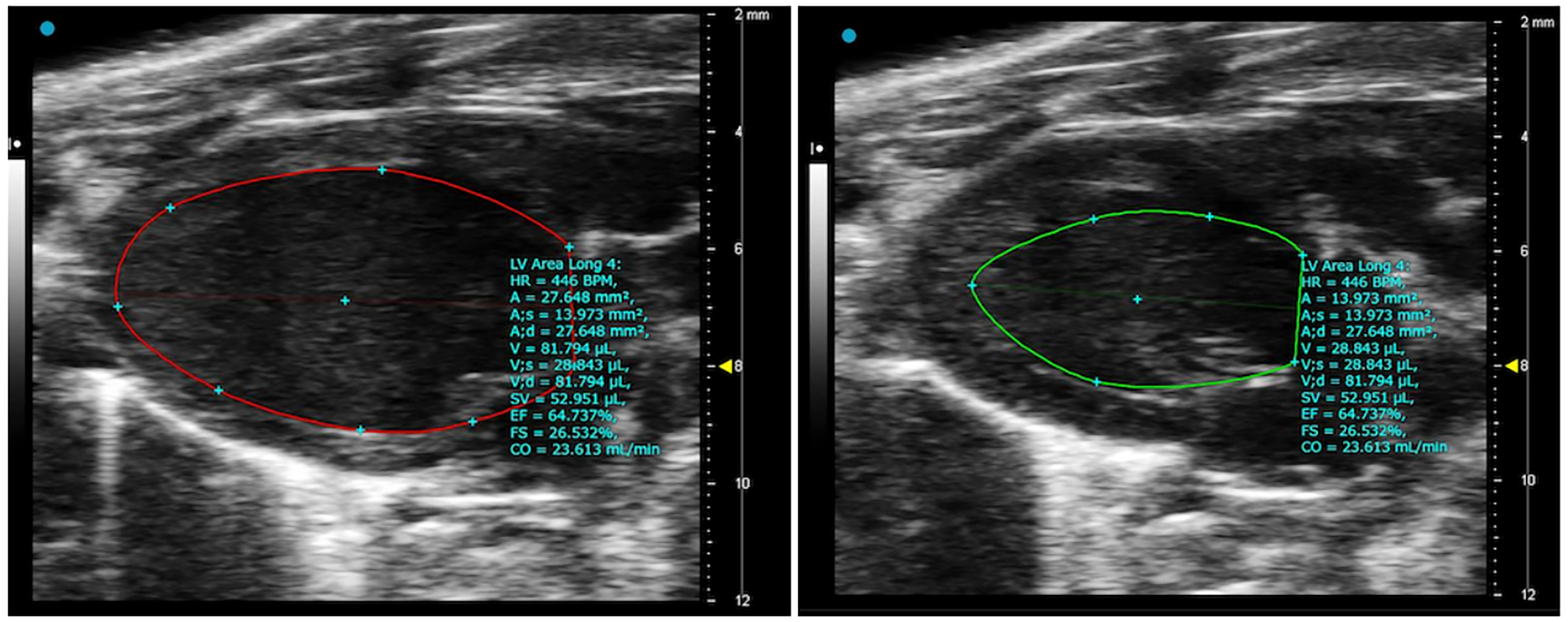

1w MI

Ejection Fraction	%	44.26457
Fractional Shortening	%	16.67113
Cardiac Output	mL/min	14.388

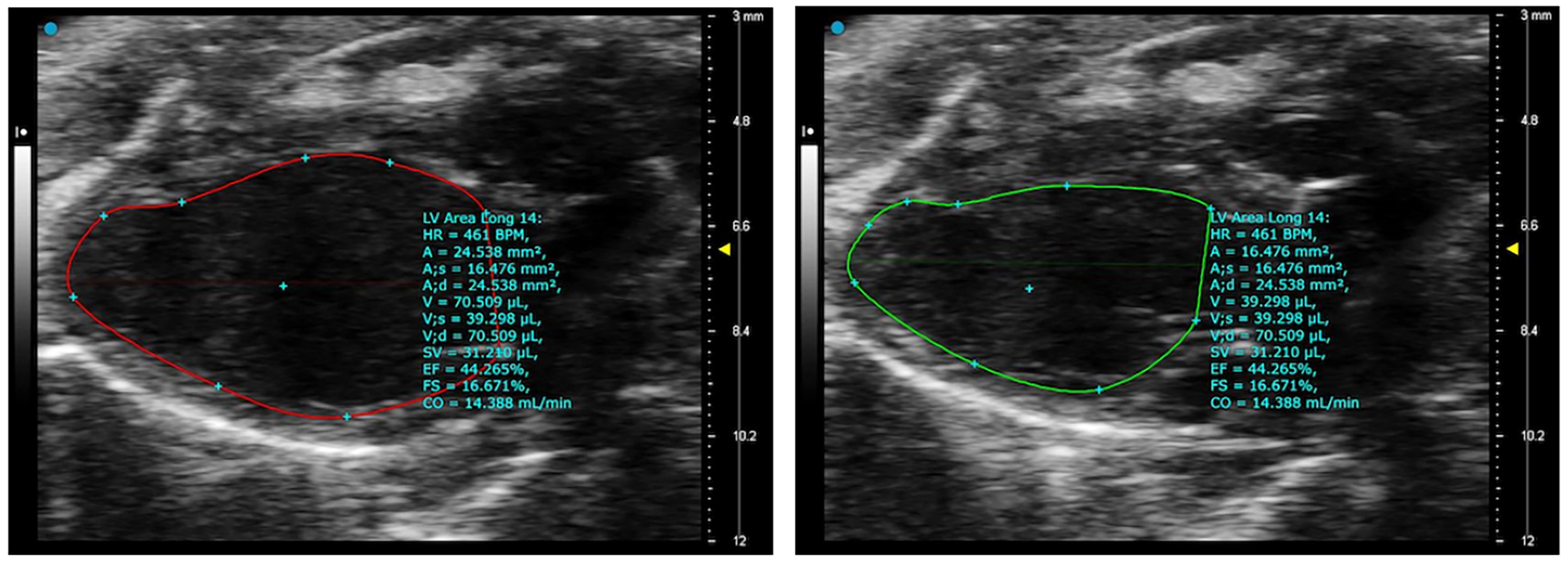

2w MI
O319 CONTROL

Ejection Fraction	%	39.83842
Fractional Shortening	%	13.4644
Cardiac Output	mL/min	14.391

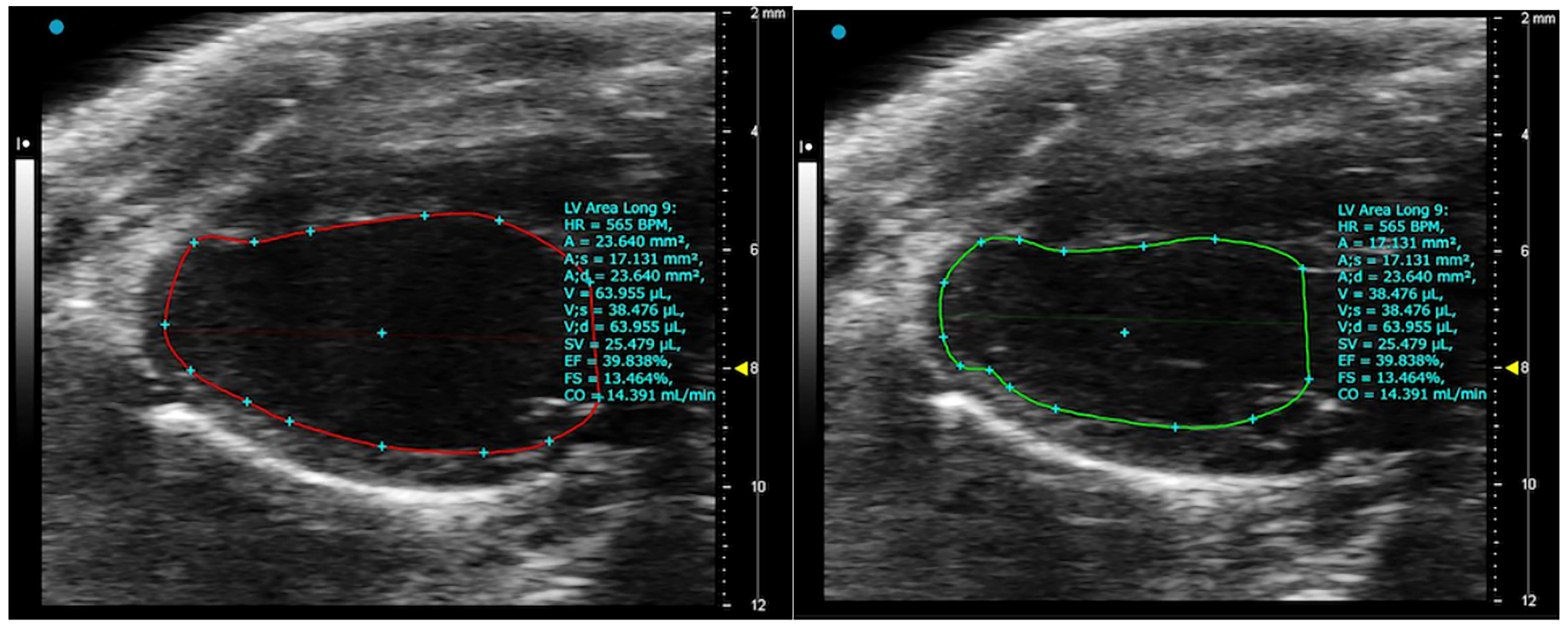

4w MI
O319 CONTROL

Ejection Fraction	%	40.4238
Fractional Shortening	%	11.38564
Cardiac Output	mL/min	14.883

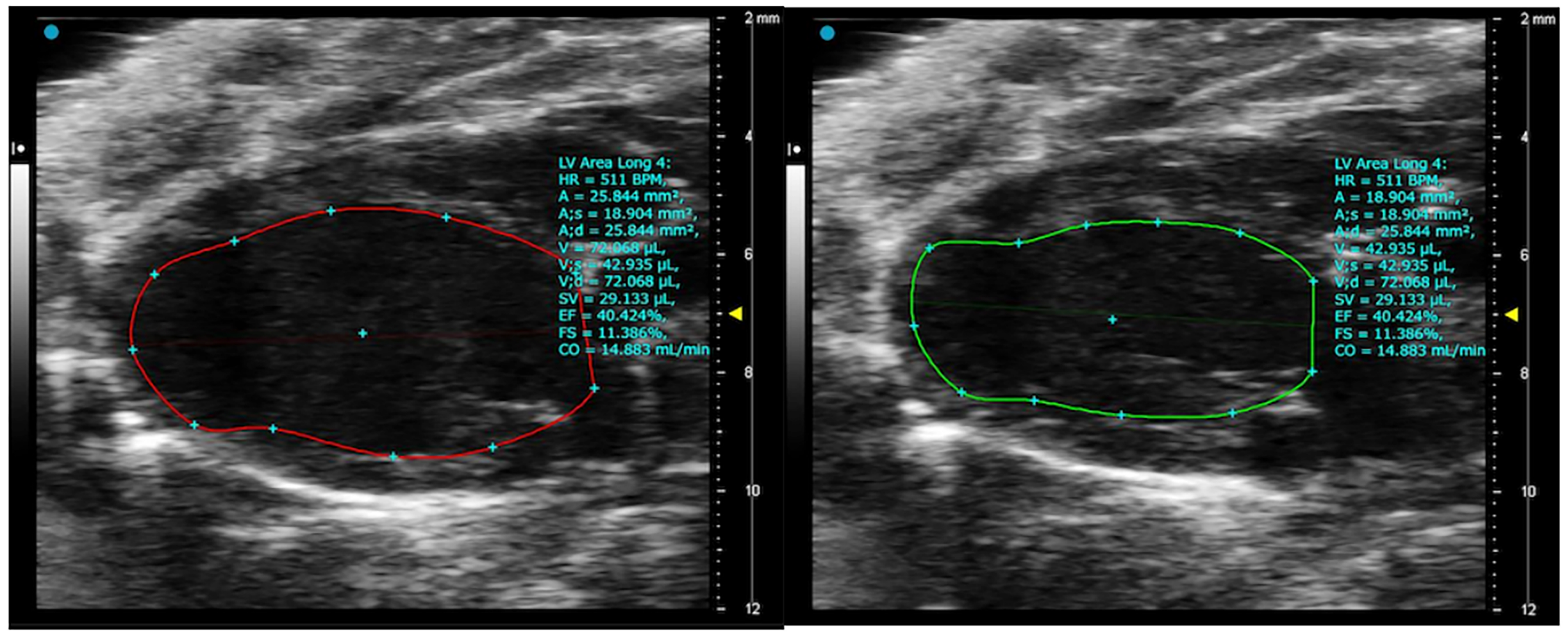

1w MI O314 CONTROL

Ejection Fraction	%	48.43932
Fractional Shortening	%	-2.65122
Cardiac Output	mL/min	15.844

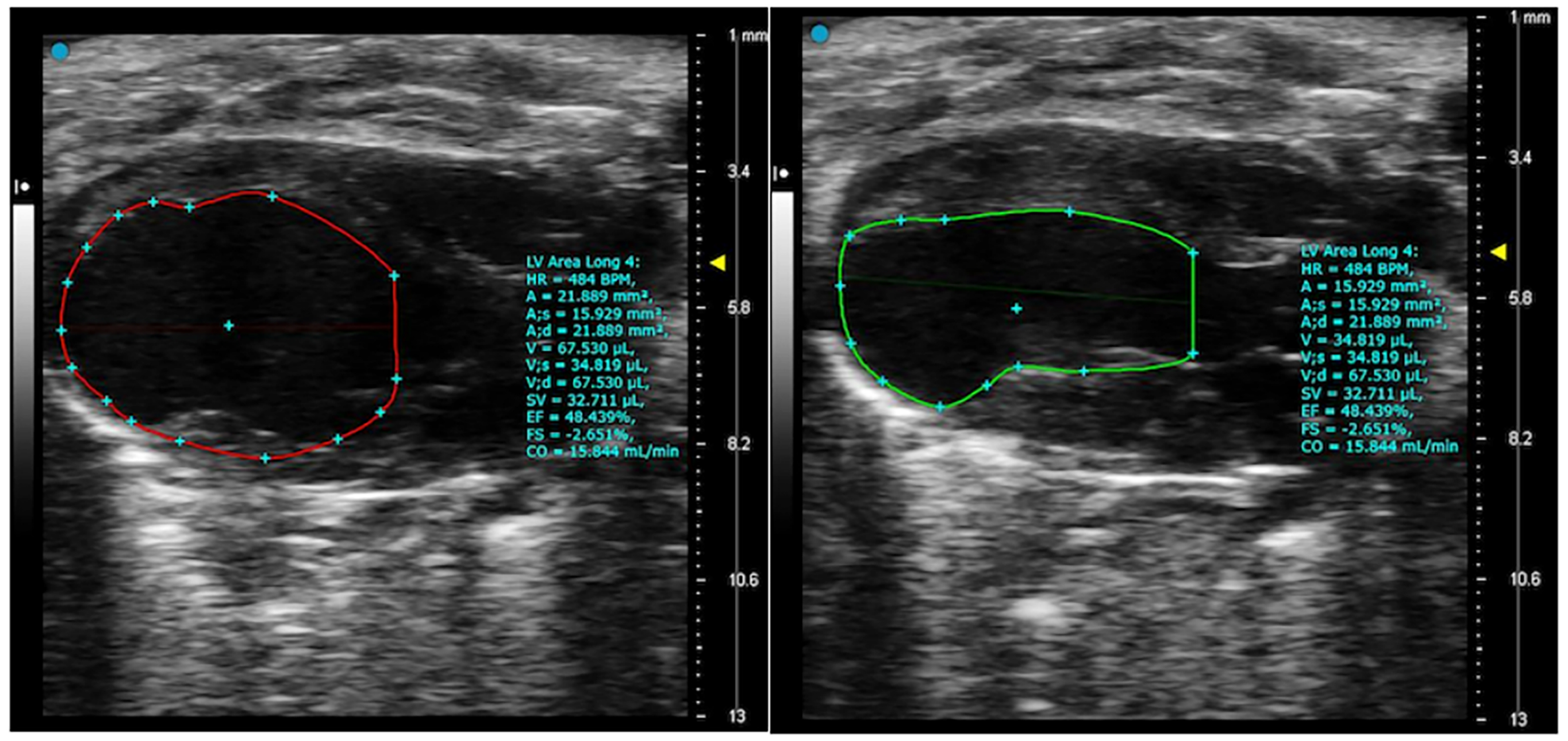

4w MI O314 CONTROL

Ejection Fraction	%	33.08802
Fractional Shortening	%	1.511358
Cardiac Output	mL/min	11.639

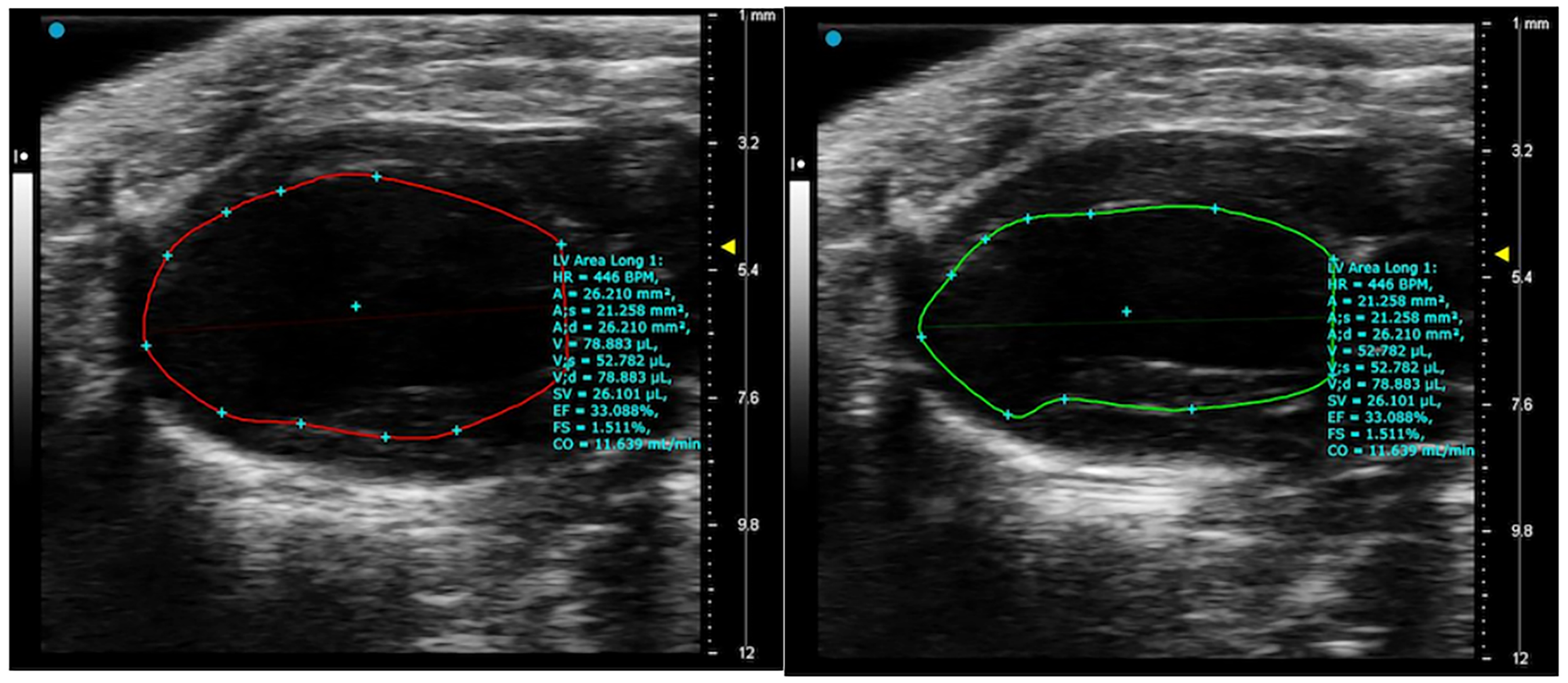

2w MI O314 CONTROL

Ejection Fraction	%	32.49801
Fractional Shortening	%	3.476976
Cardiac Output	mL/min	11.008

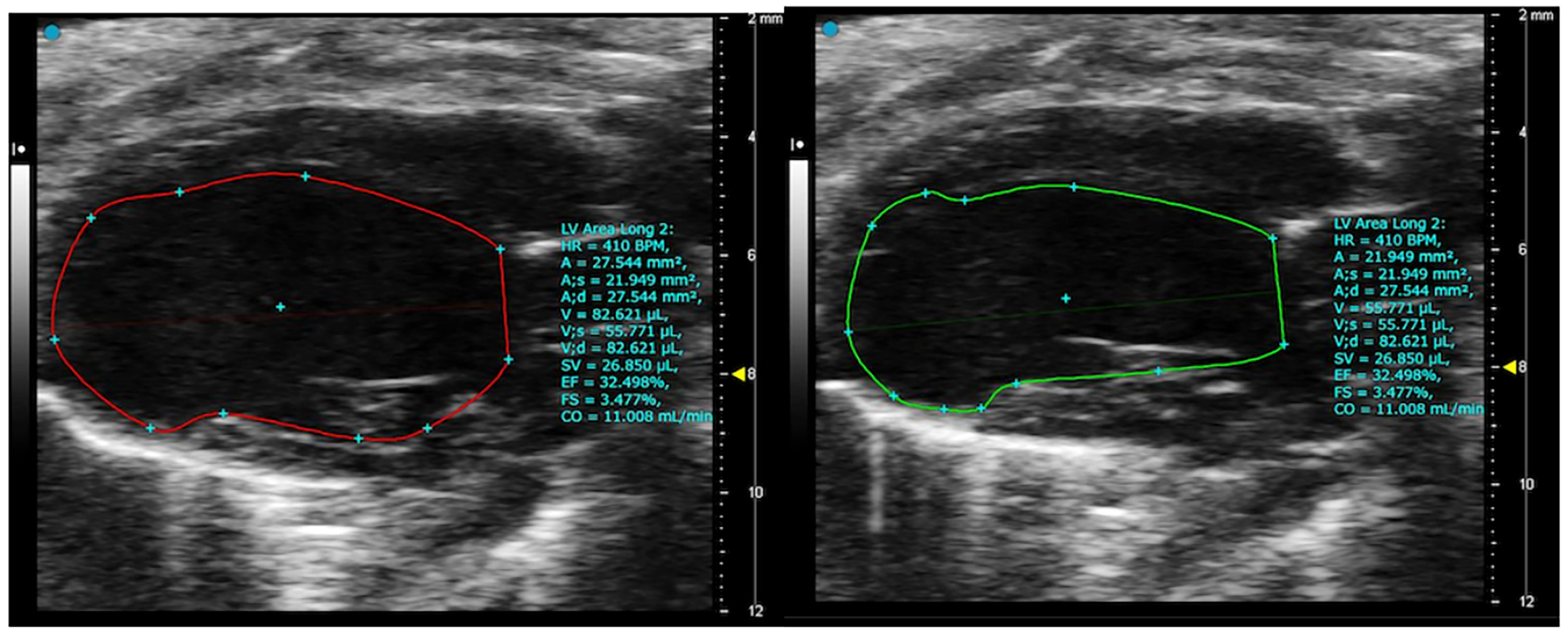

Before MI

P576 STEMIN + YAP5SA

B-Mode	Ejection Fraction	%	52.488691
B-Mode	Fractional Shortening	%	12.411797
B-Mode	Cardiac Output	mL/min	19.116275

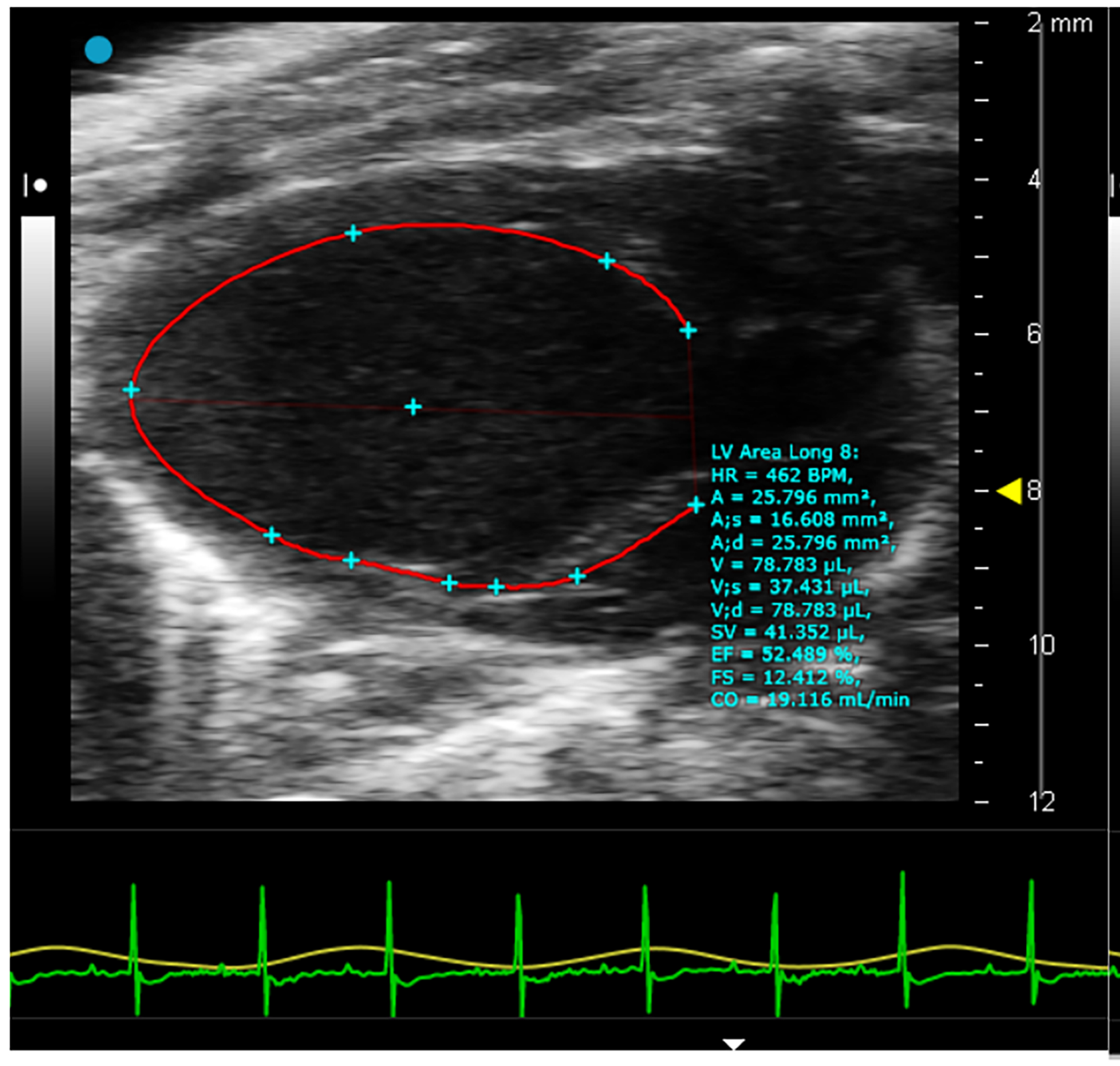

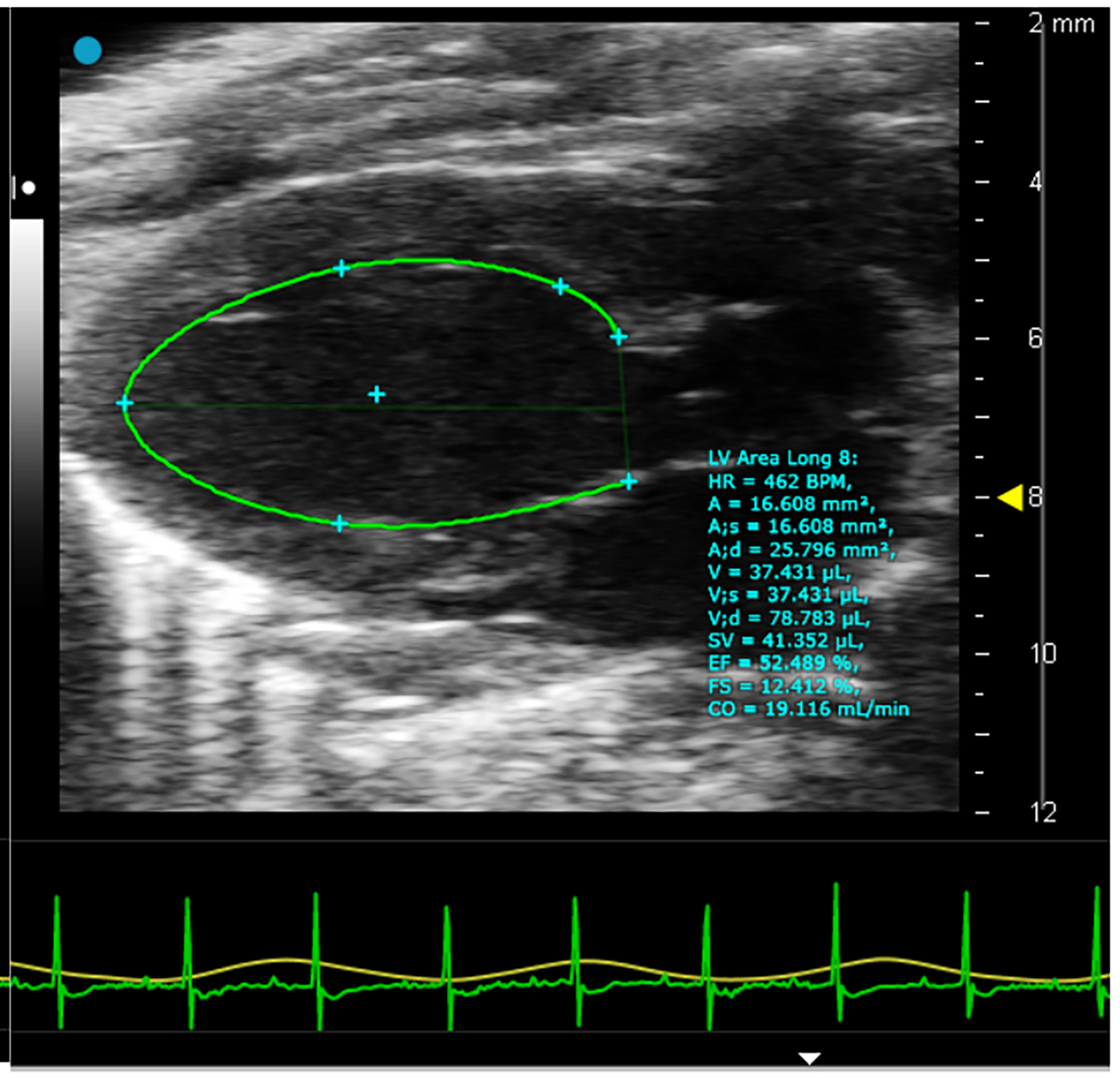

2 week MI

P576 STEMIN + YAP5SA

B-Mode	Ejection Fraction	%	55.142924
B-Mode	Fractional Shortening	%	22.436617
B-Mode	Cardiac Output	mL/min	16.346074

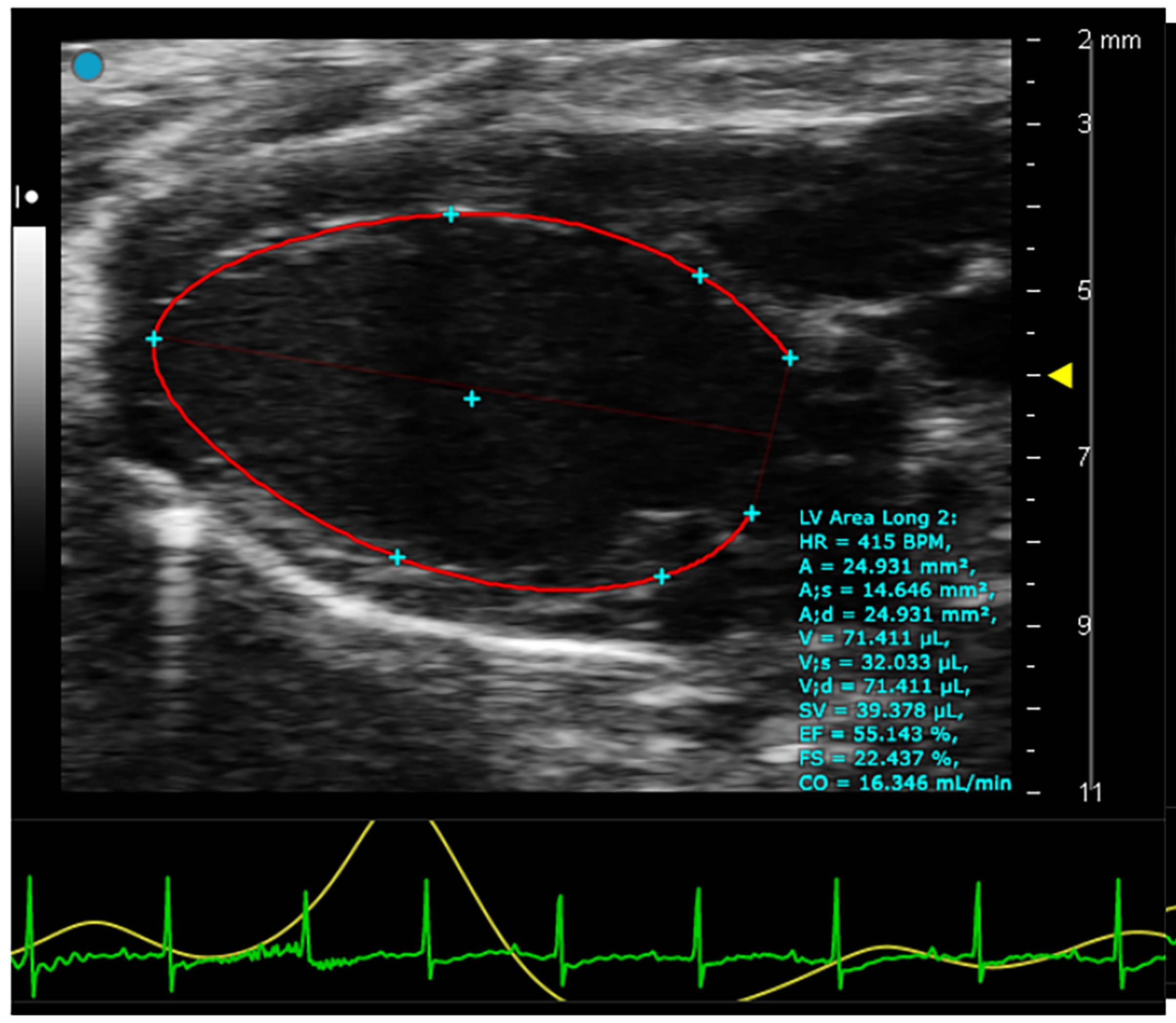

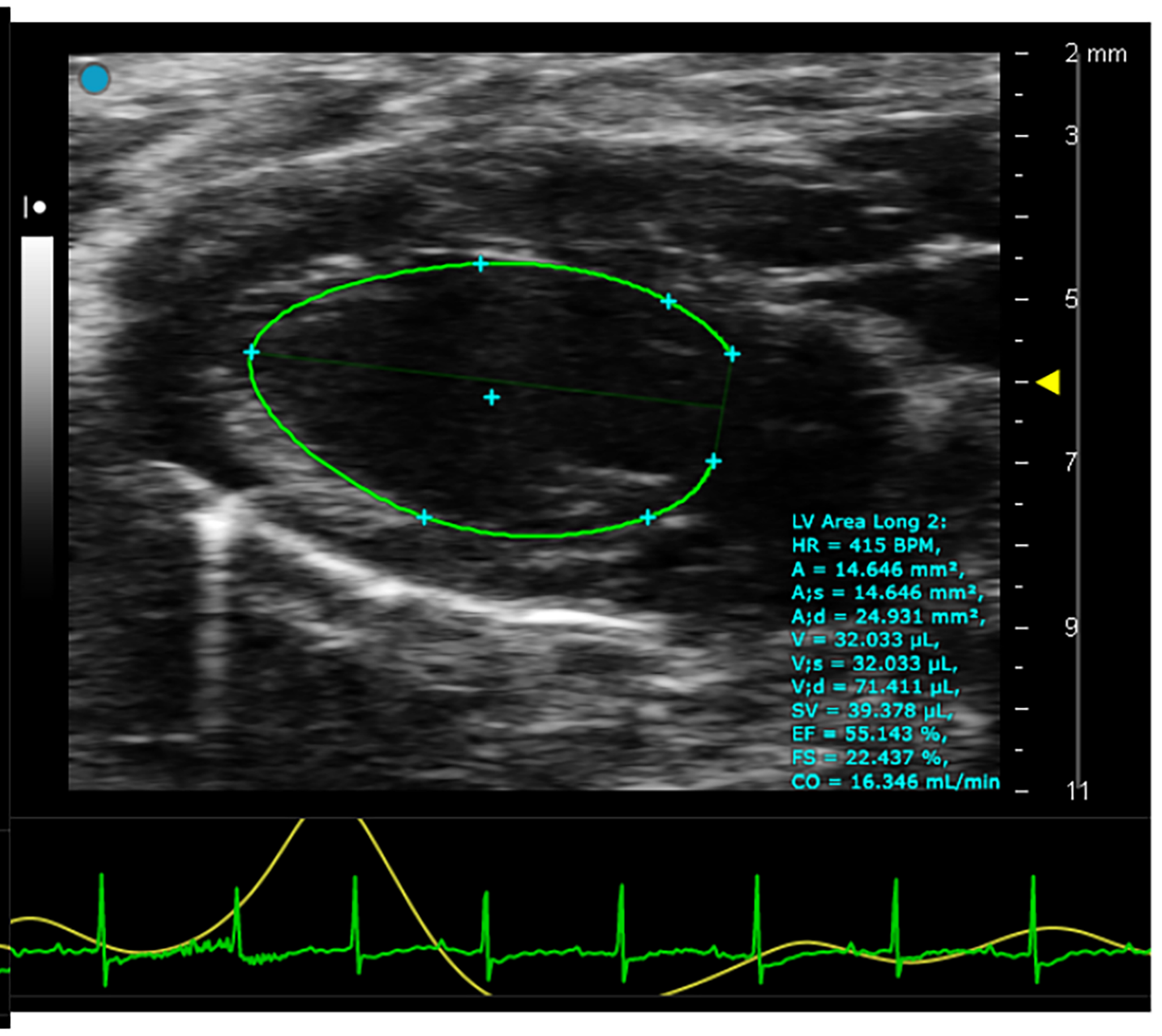

4 week MI

P576 STEMIN + YAP5SA

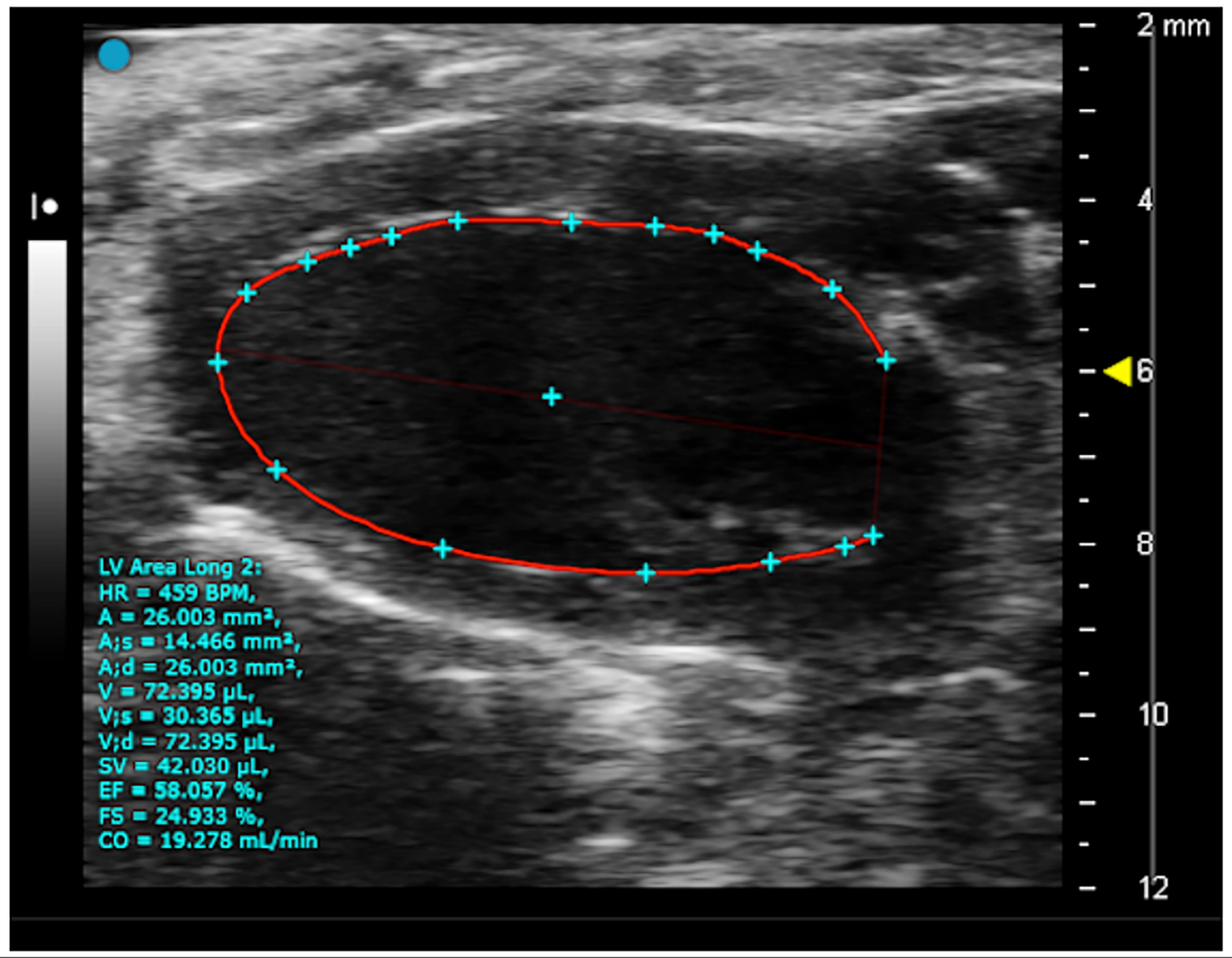

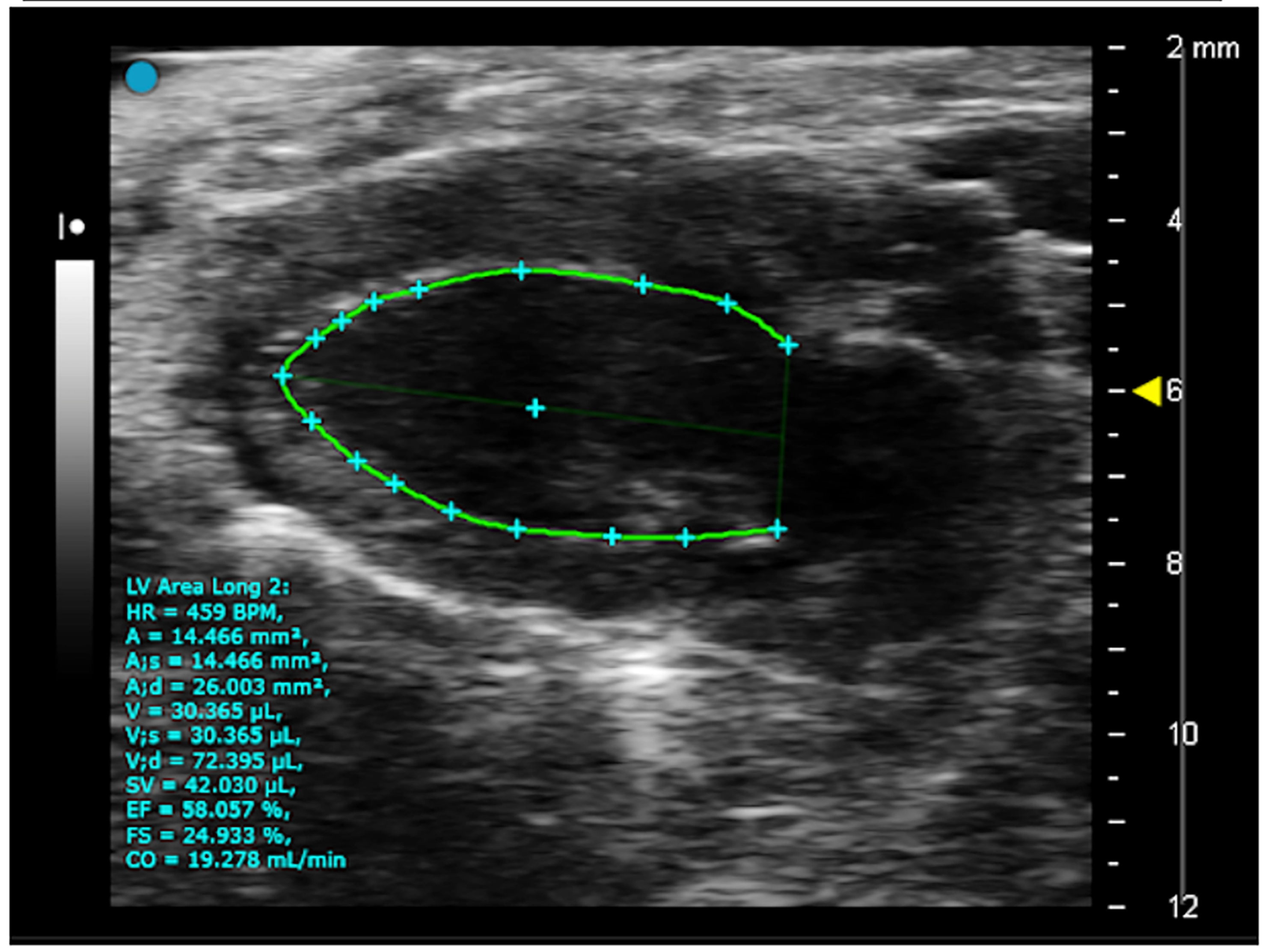

B-Mode	Ejection Fraction	%	58.057
B-Mode	Fractional Shortening	%	24.933
B-Mode	Cardiac Output	mL/min	19.278

Before MI
O317 Stemin + YAP5SA

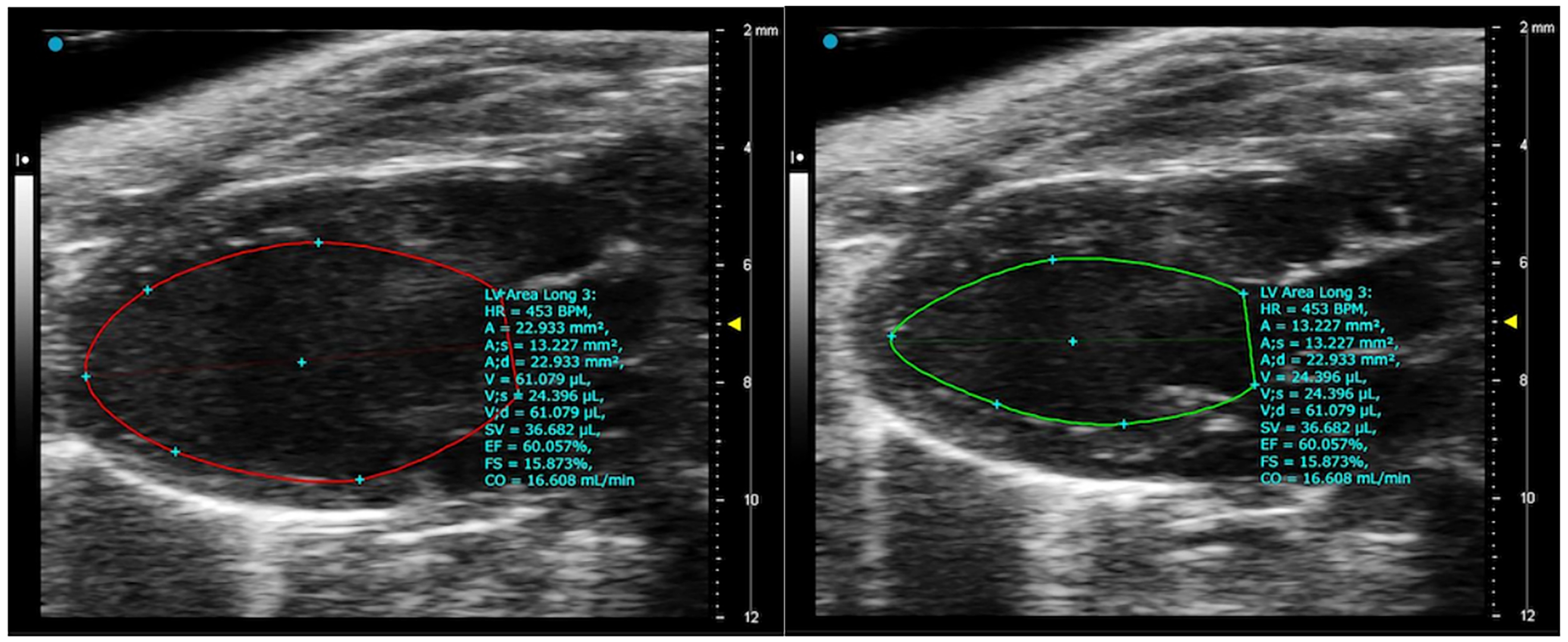

Ejection Fraction	%	60.0575
Fractional Shortening	%	15.87285
Cardiac Output	mL/min	16.608

1w MI
O317 Stemin + YAP5SA

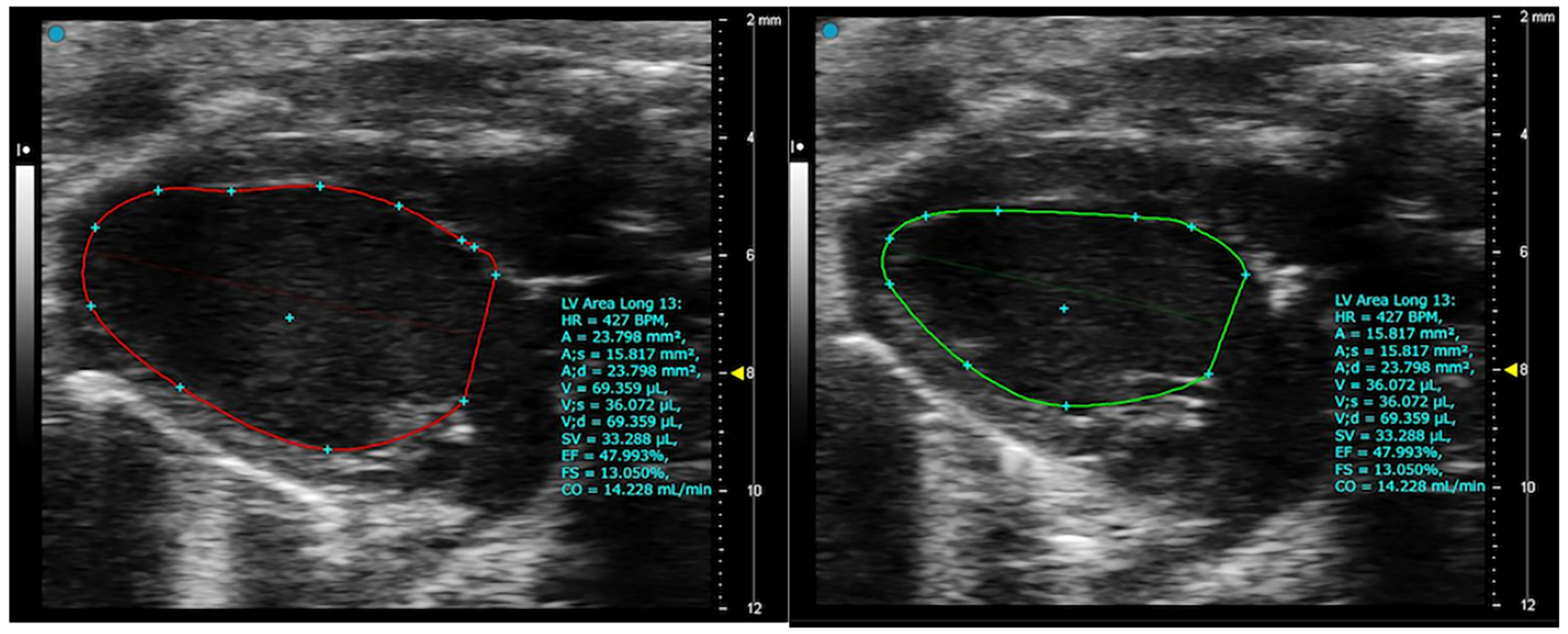

Ejection Fraction	%	47.99297
Fractional Shortening	%	13.05014
Cardiac Output	mL/min	14.228

2w MI
O317 Stemin + YAP5SA

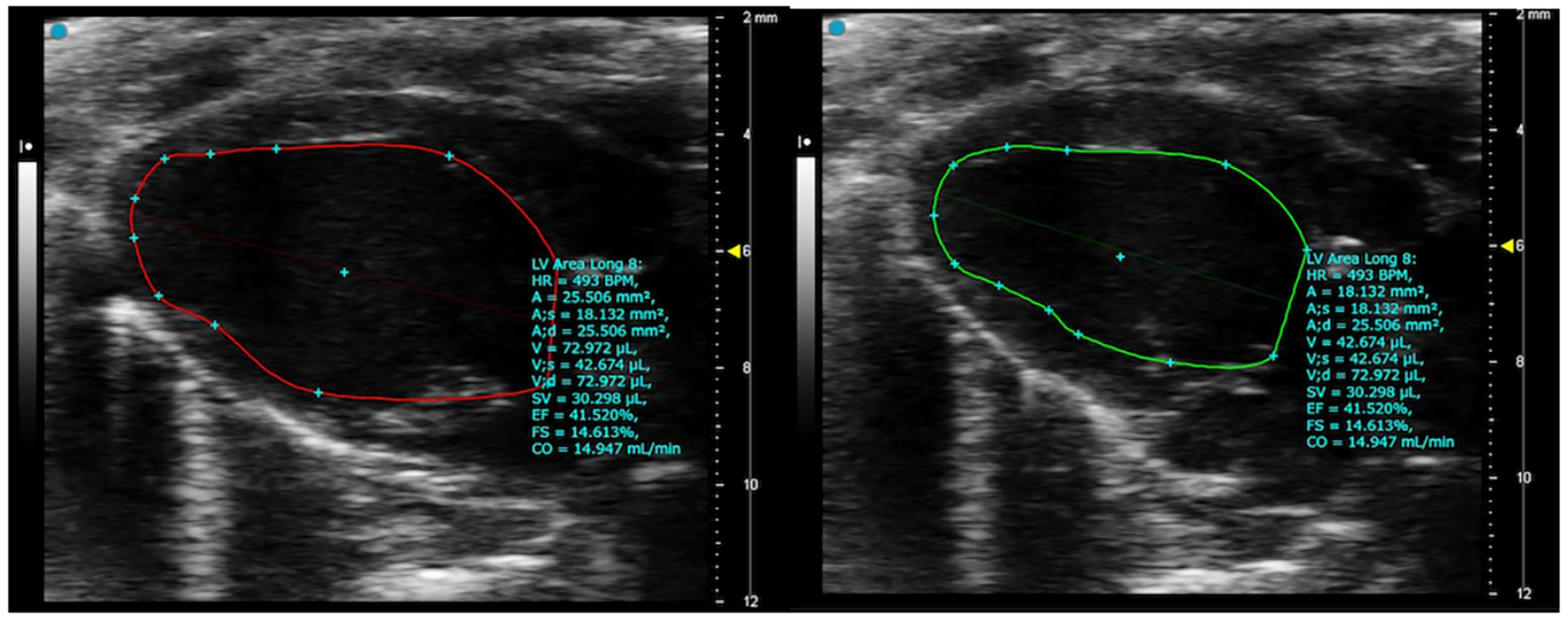

Ejection Fraction	%	41.51972
Fractional Shortening	%	14.61276
Cardiac Output	mL/min	14.947

4w MI
O317 Stemin + YAP5SA

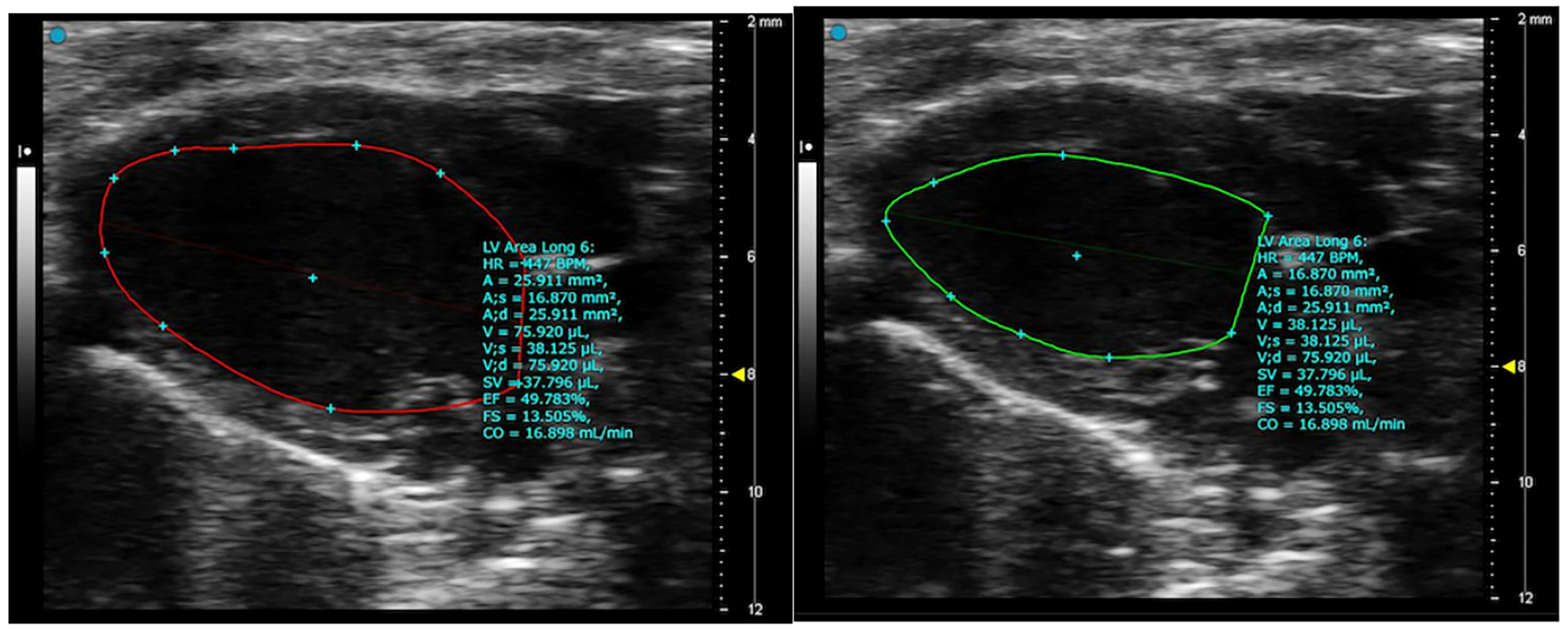

Ejection Fraction	%	49.78327
Fractional Shortening	%	13.50544
Cardiac Output	mL/min	16.898

Before MI

O318 Stemin + YAP5SA

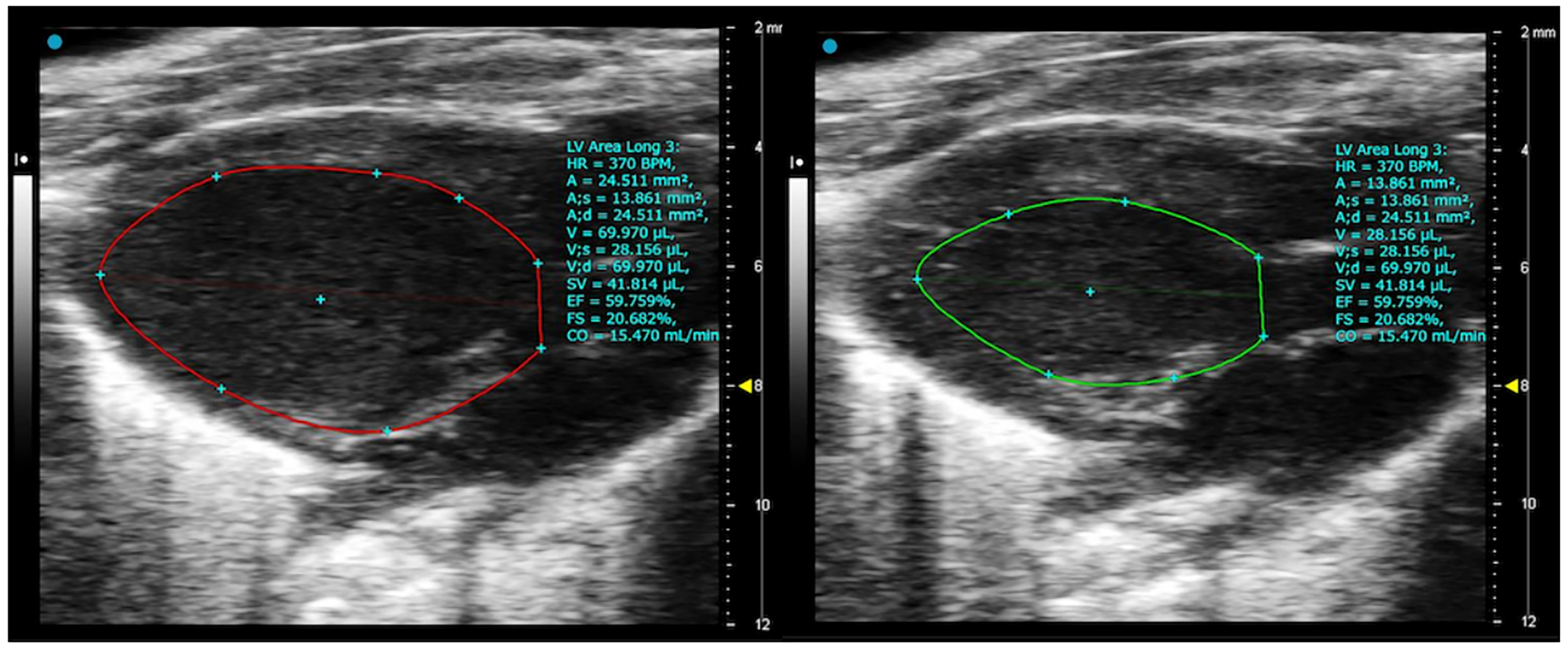

Ejection Fraction	%	59.75938
Fractional Shortening	%	20.68155
Cardiac Output	mL/min	15.470

1w MI

O318 Stemin + YAP5SA

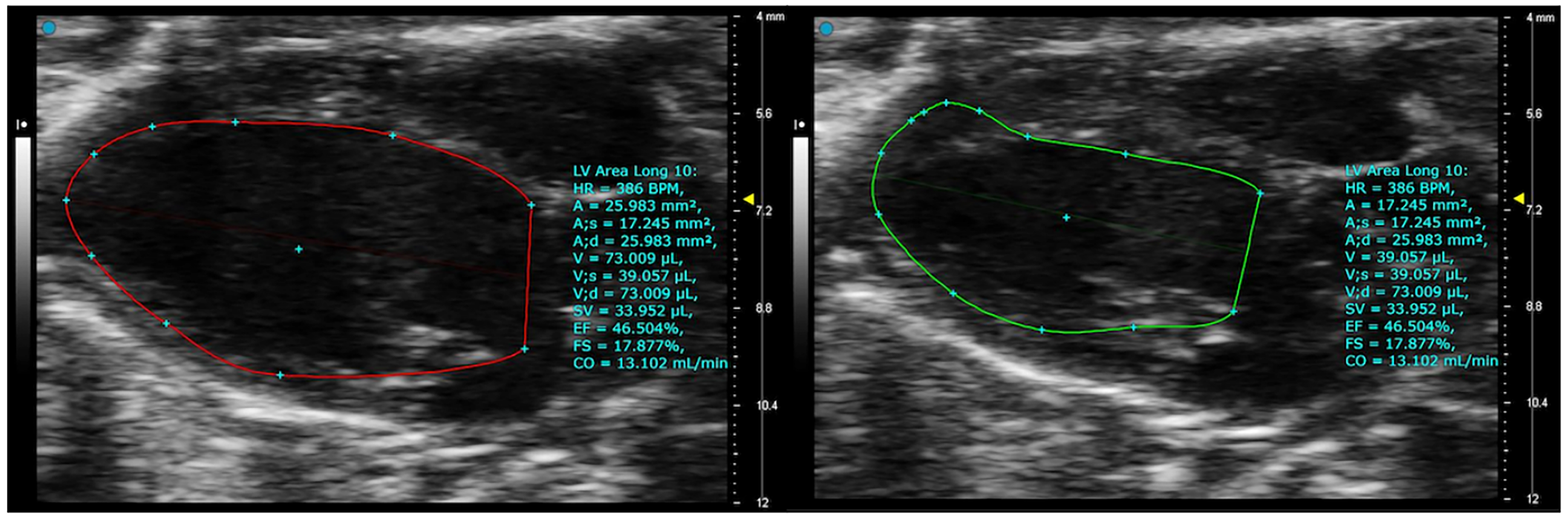

Ejection Fraction	%	46.50387
Fractional Shortening	%	17.87729
Cardiac Output	mL/min	13.102

2w MI

O318 Stemin + YAP5SA

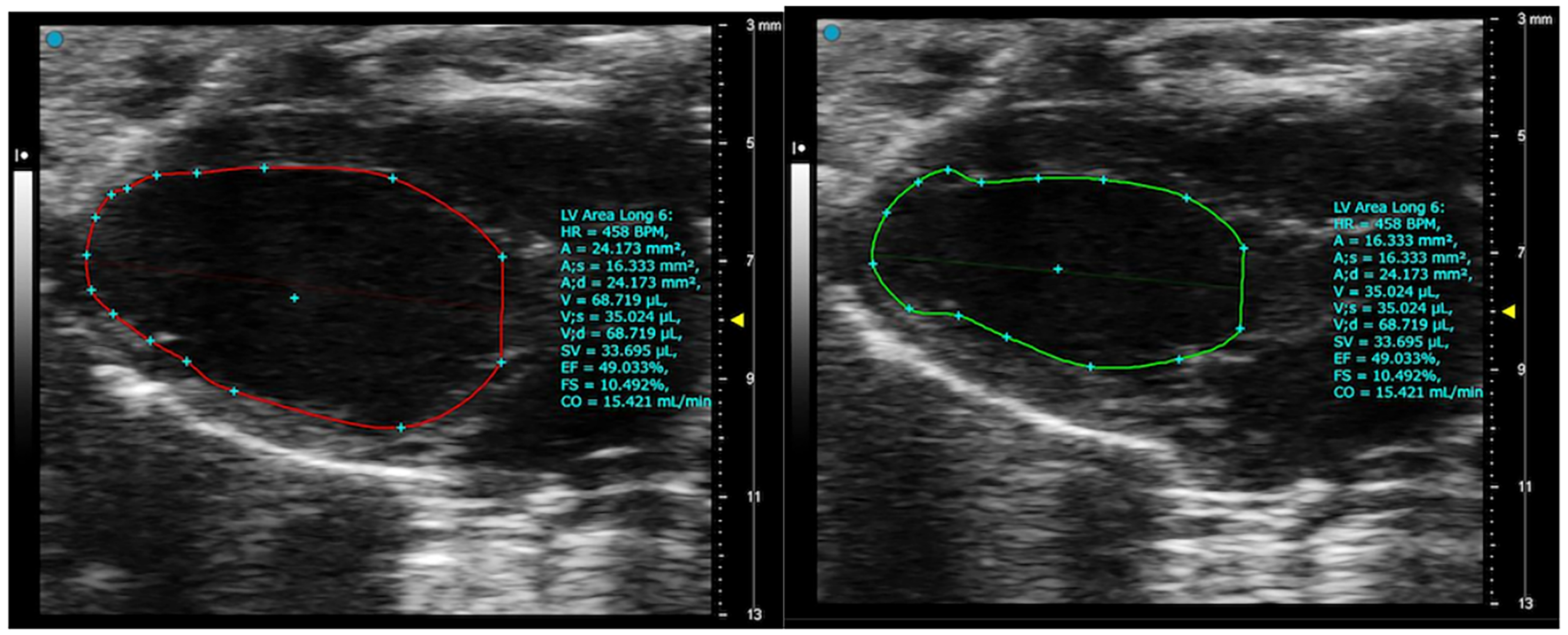

Ejection Fraction	%	49.03272
Fractional Shortening	%	10.49169
Cardiac Output	mL/min	15.421

4w MI

O318 Stemin + YAP5SA

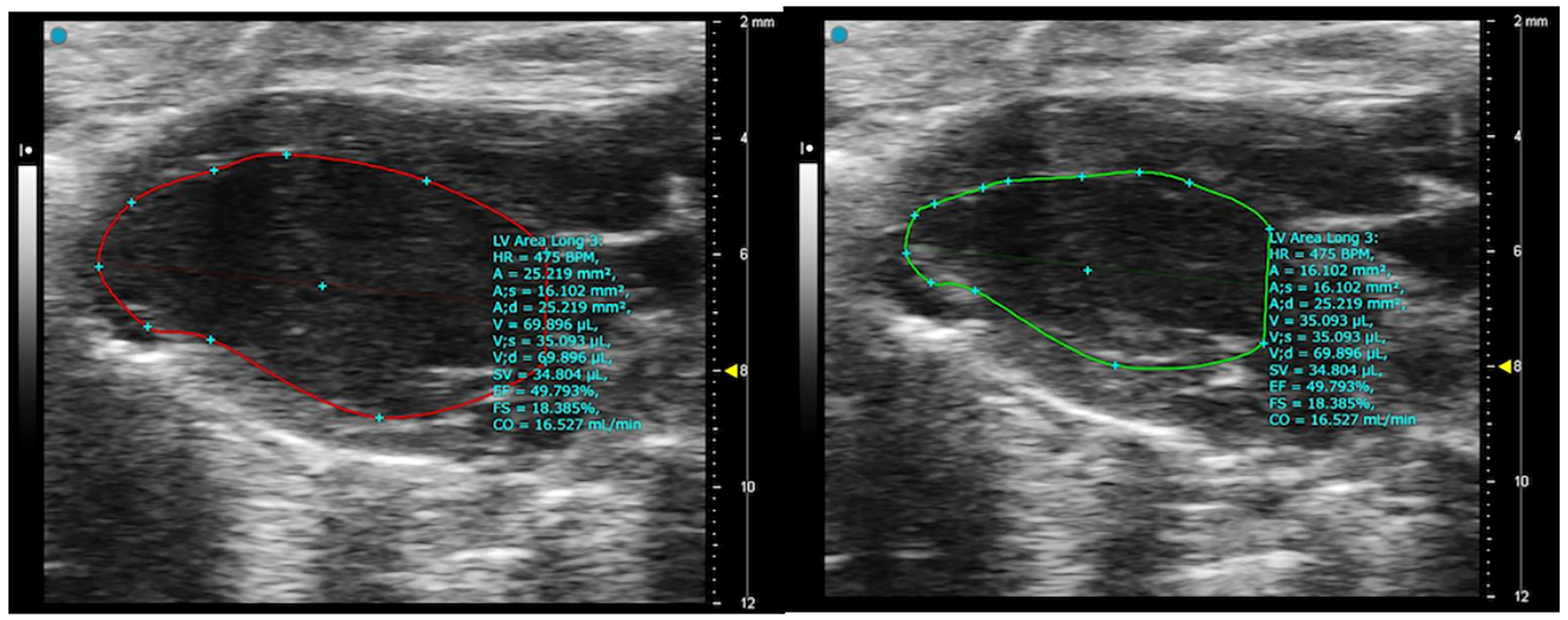

Ejection Fraction	%	49.79332
Fractional Shortening	%	18.38548
Cardiac Output	mL/min	16.527

Before MI

O315 Stemin + YAP5SA

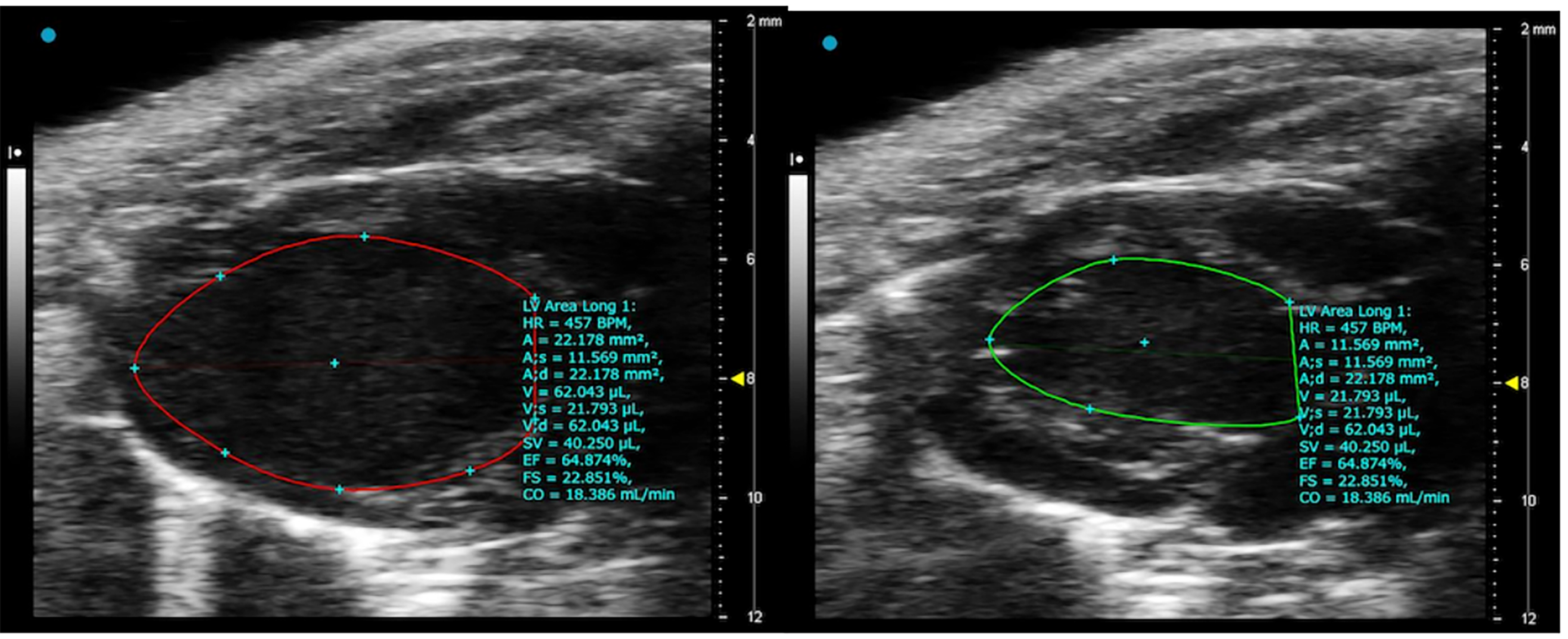

Ejection Fraction	%	64.87406
Fractional Shortening	%	22.85075
Cardiac Output	mL/min	18.386

2w MI

O315 Stemin + YAP5SA

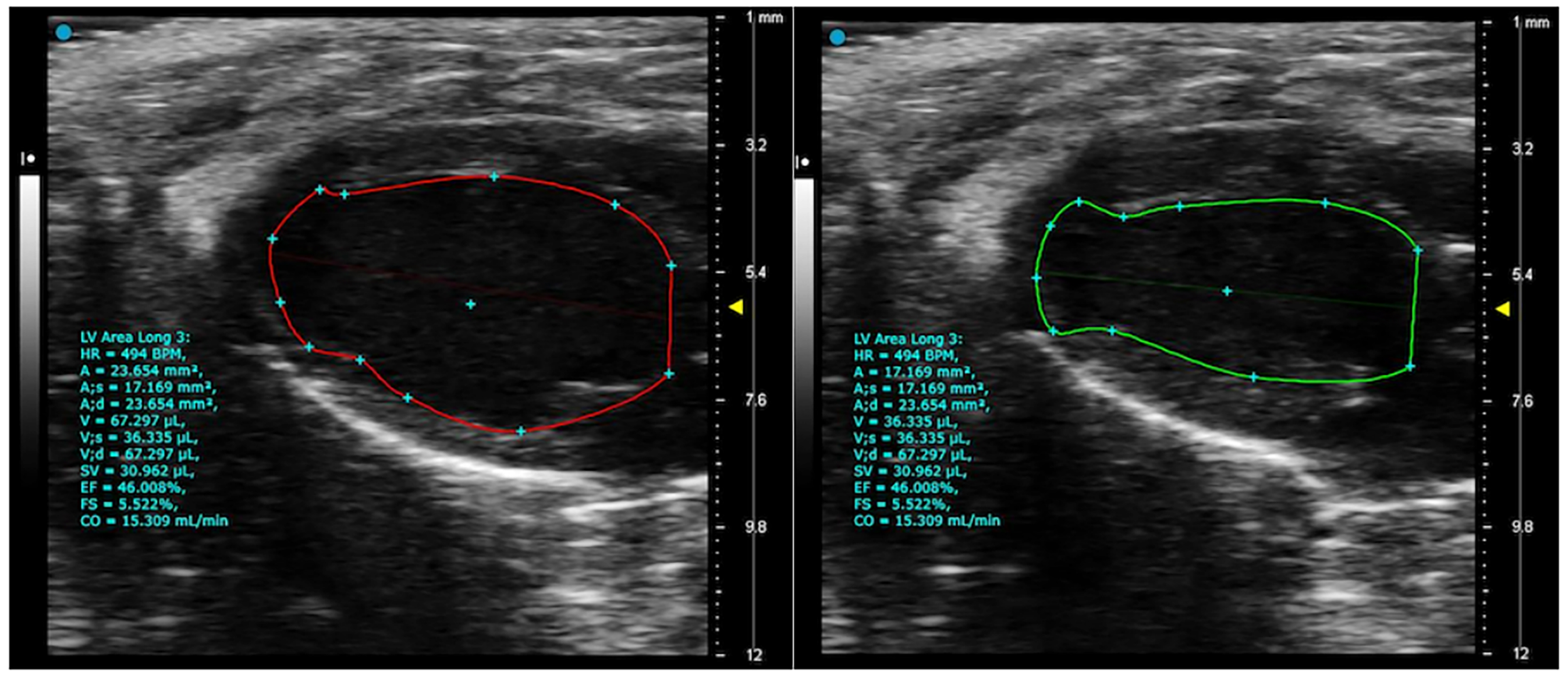

Ejection Fraction	%	46.00824
Fractional Shortening	%	5.521986
Cardiac Output	mL/min	15.309

1w MI

O315 Stemin + YAP5SA

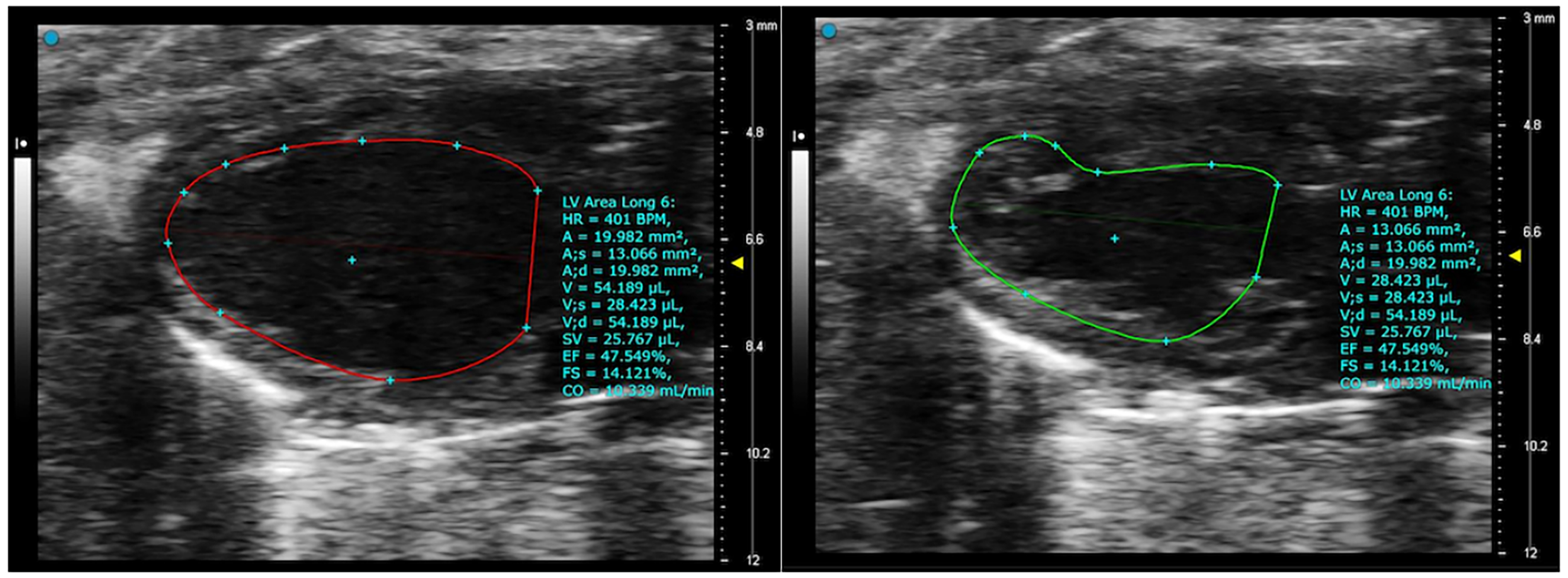

Ejection Fraction	%	47.54934
Fractional Shortening	%	14.12106
Cardiac Output	mL/min	10.339

4w MI

O315 Stemin + YAP5SA

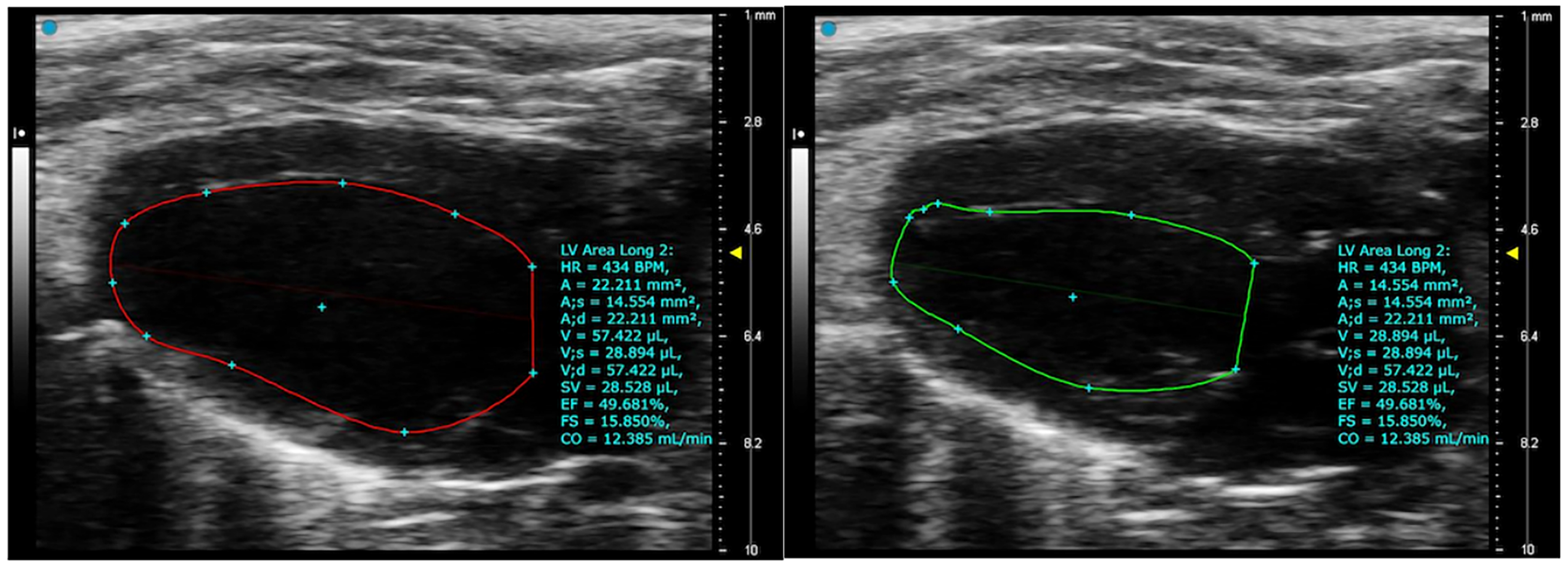

Ejection Fraction	%	49.68097
Fractional Shortening	%	15.84982
Cardiac Output	mL/min	12.385

Before MI
STEMIN alone

B-Mode	Ejection Fraction	%	57.807451
B-Mode	Fractional Shortening	%	16.760429
B-Mode	Cardiac Output	mL/min	24.694003

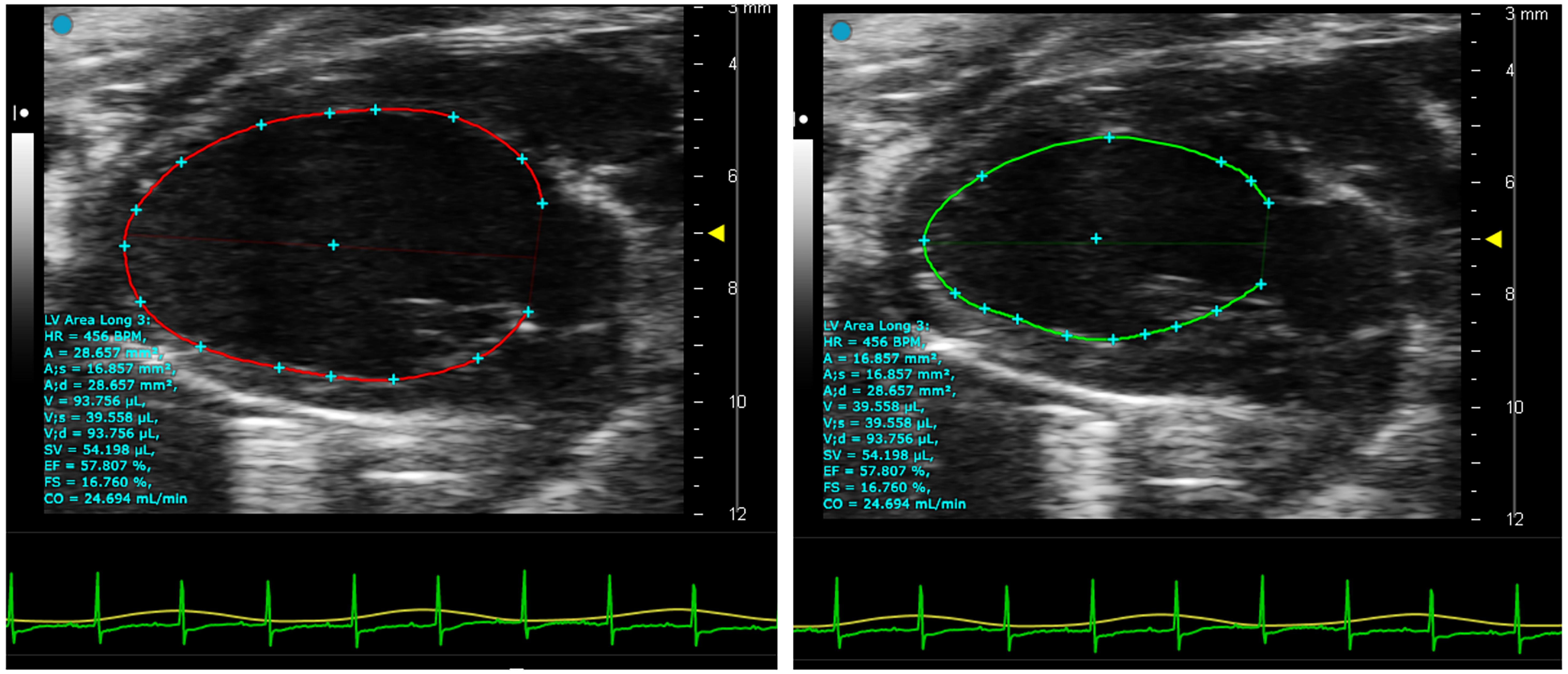

4 week M
STEMIN alone

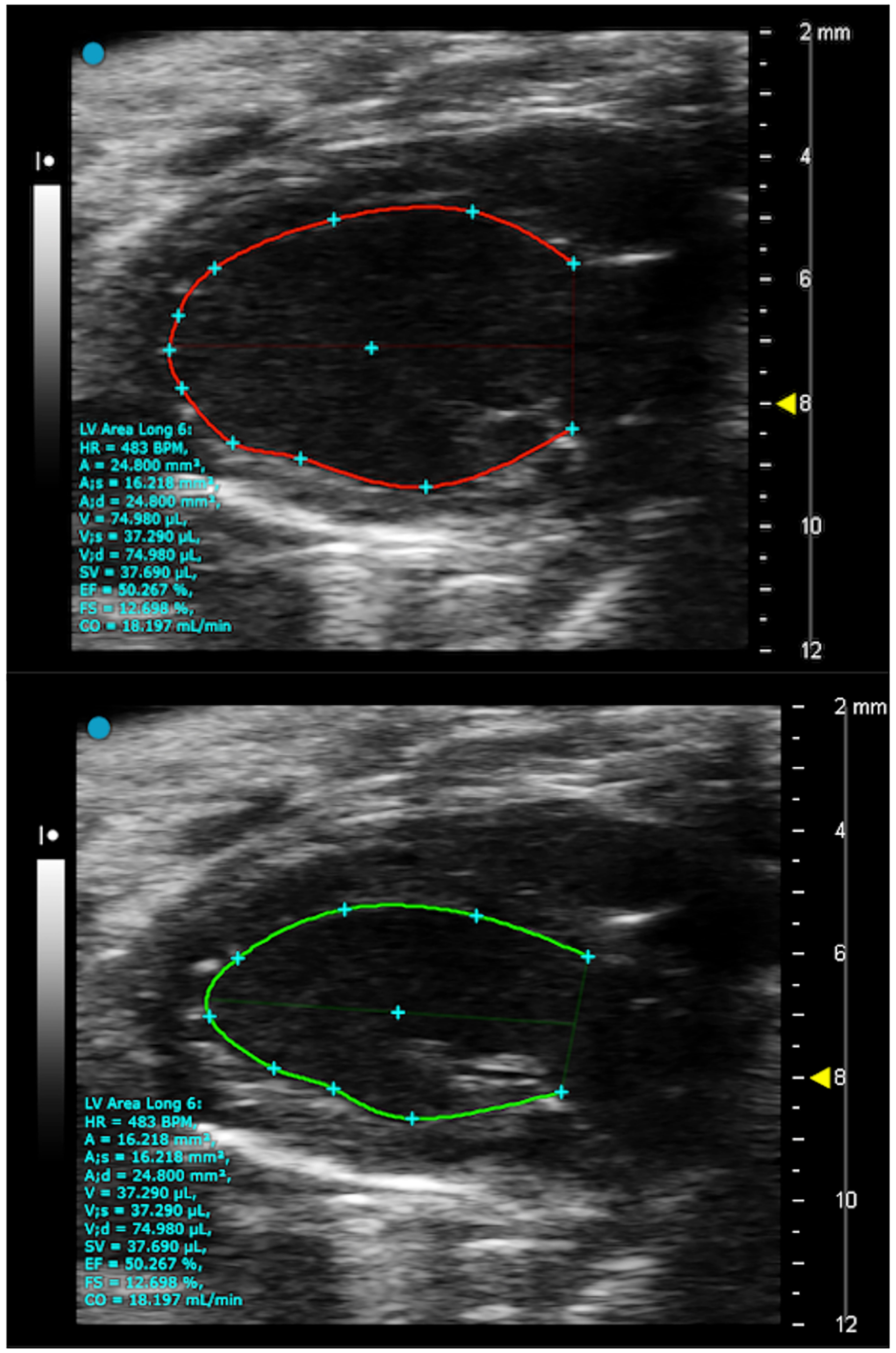

B-Mode	Ejection Fraction	%	50.267
B-Mode	Fractional Shortening	%	12.698
B-Mode	Cardiac Output	mL/min	18.197

2 week MI
STEMIN alone

B-Mode	Ejection Fraction	%	44.023944
B-Mode	Fractional Shortening	%	12.463102
B-Mode	Cardiac Output	mL/min	14.763156

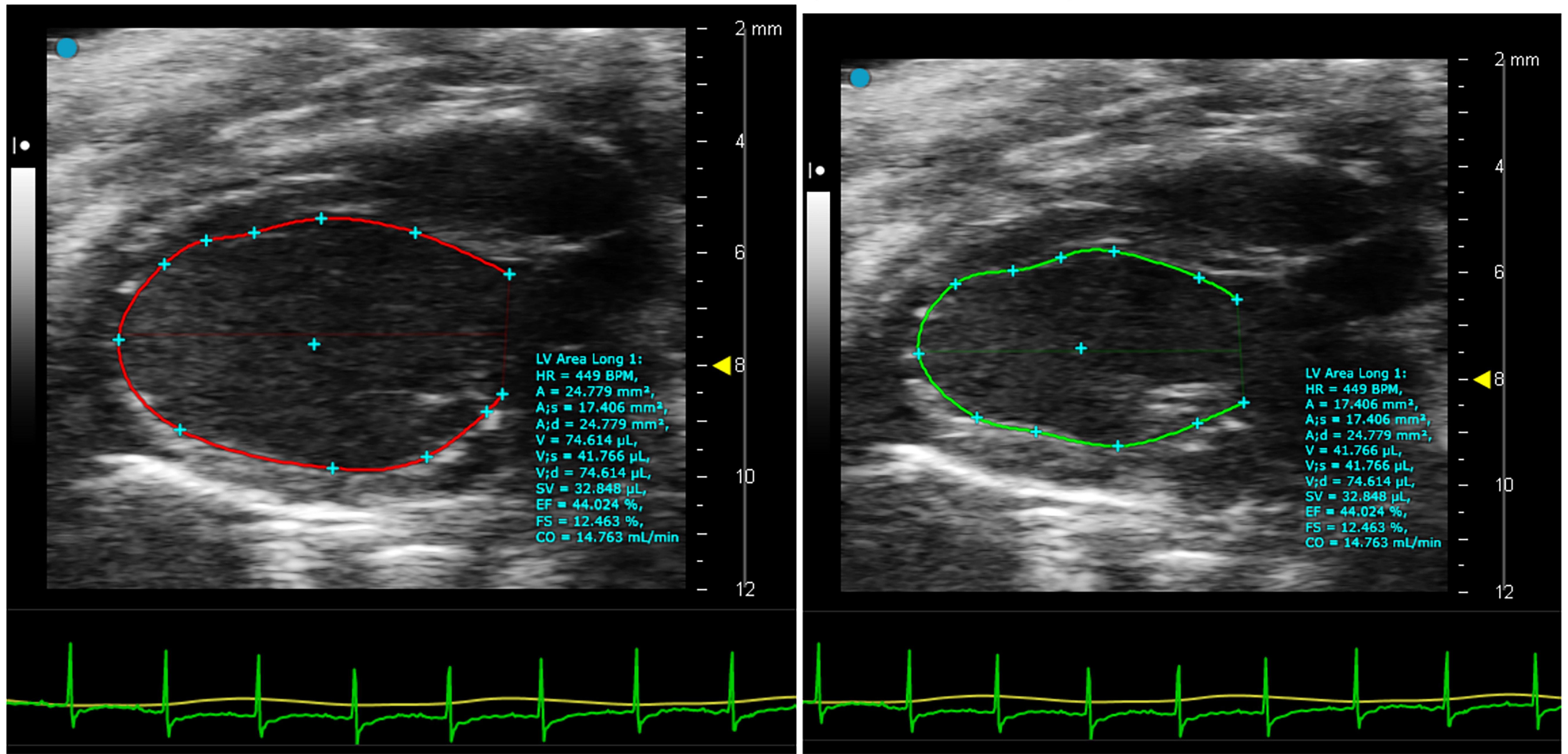

Before MI
P853 Stemin alone

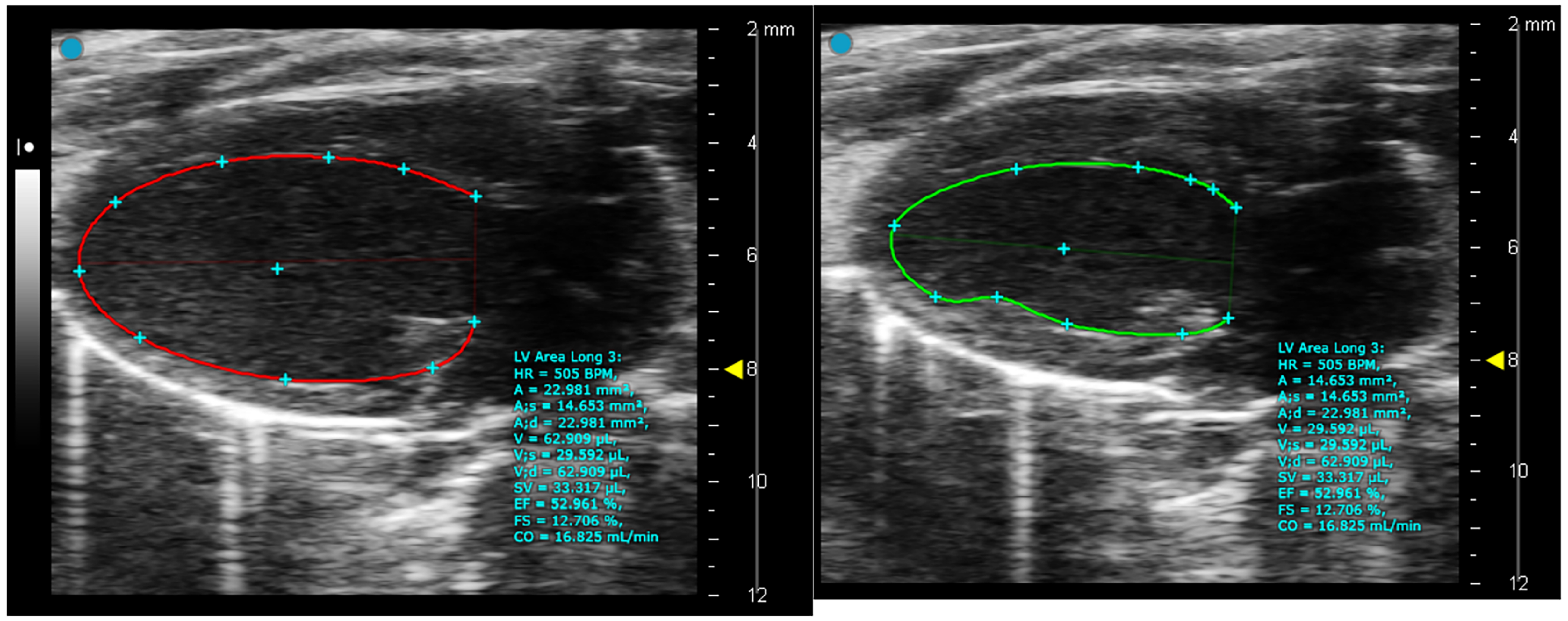

Ejection Fraction	%	52.960533
Fractional Shortening	%	12.706258
Cardiac Output	mL/min	16.824865

4w MI
P853 Stemin alone

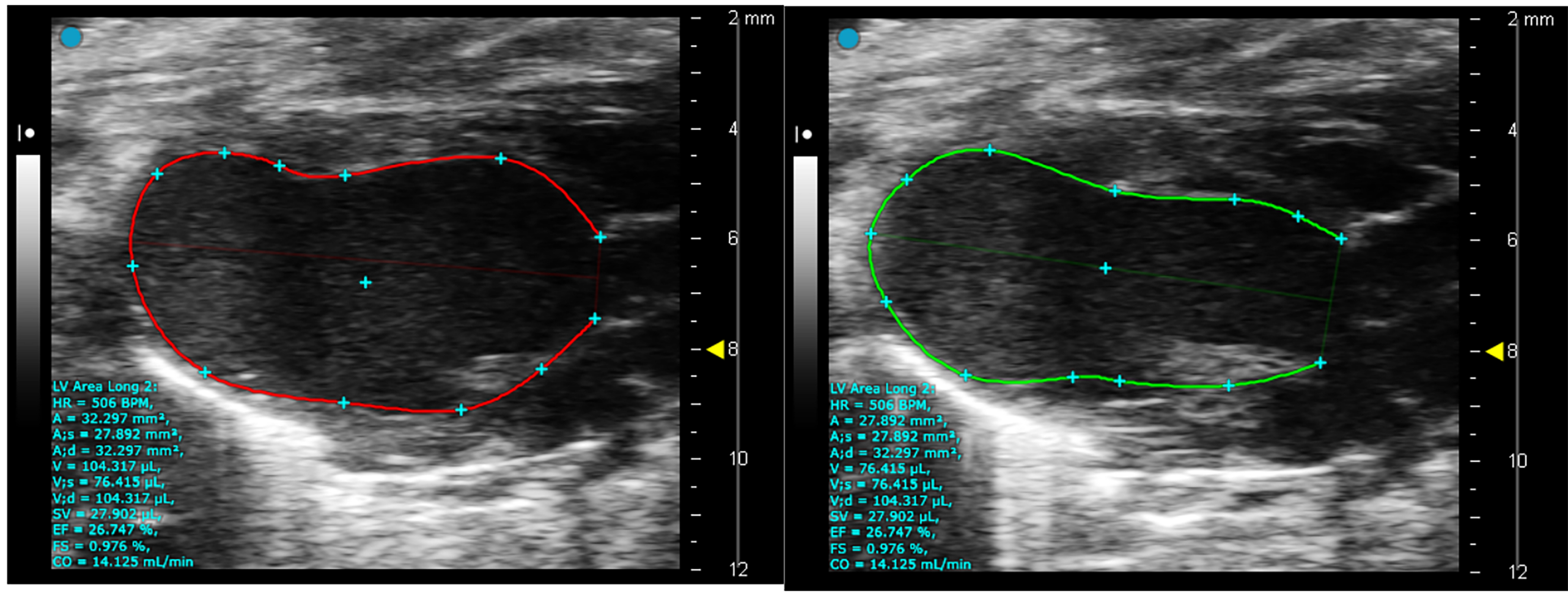

Ejection Fraction	%	26.747372
Fractional Shortening	%	0.976116
Cardiac Output	mL/min	14.125081

1w MI
P853 Stemin alone

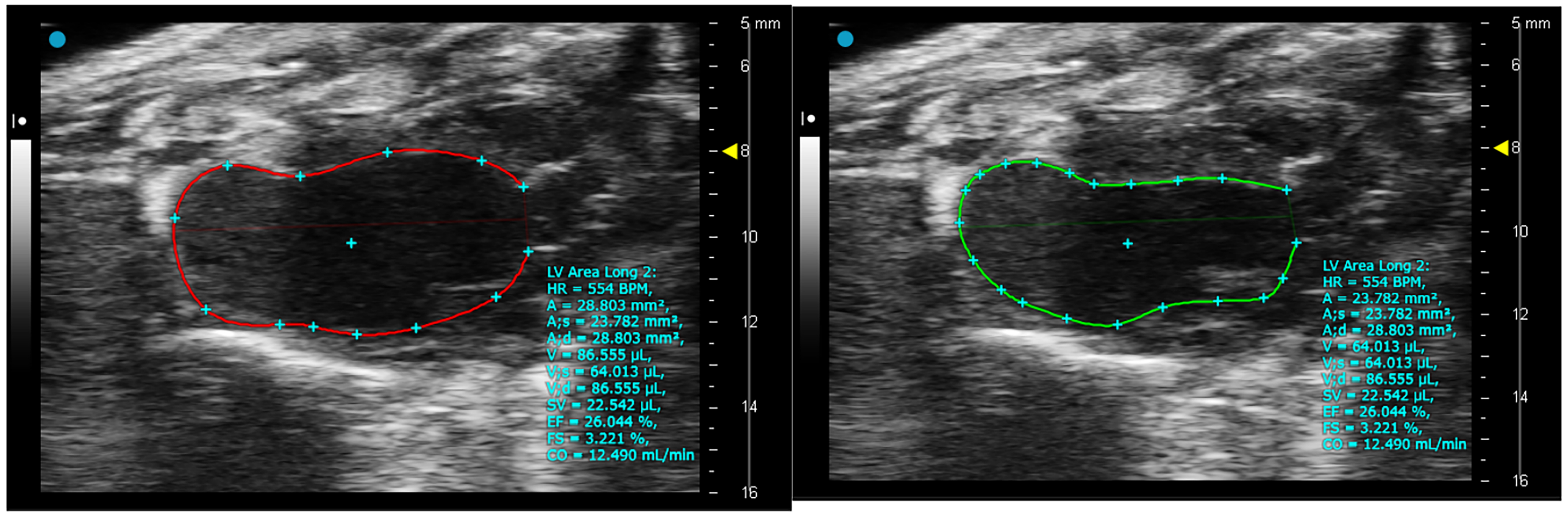

Ejection Fraction	%	26.043978
Fractional Shortening	%	3.221046
Cardiac Output	mL/min	12.48985

Before MI

P854 Stemin alone

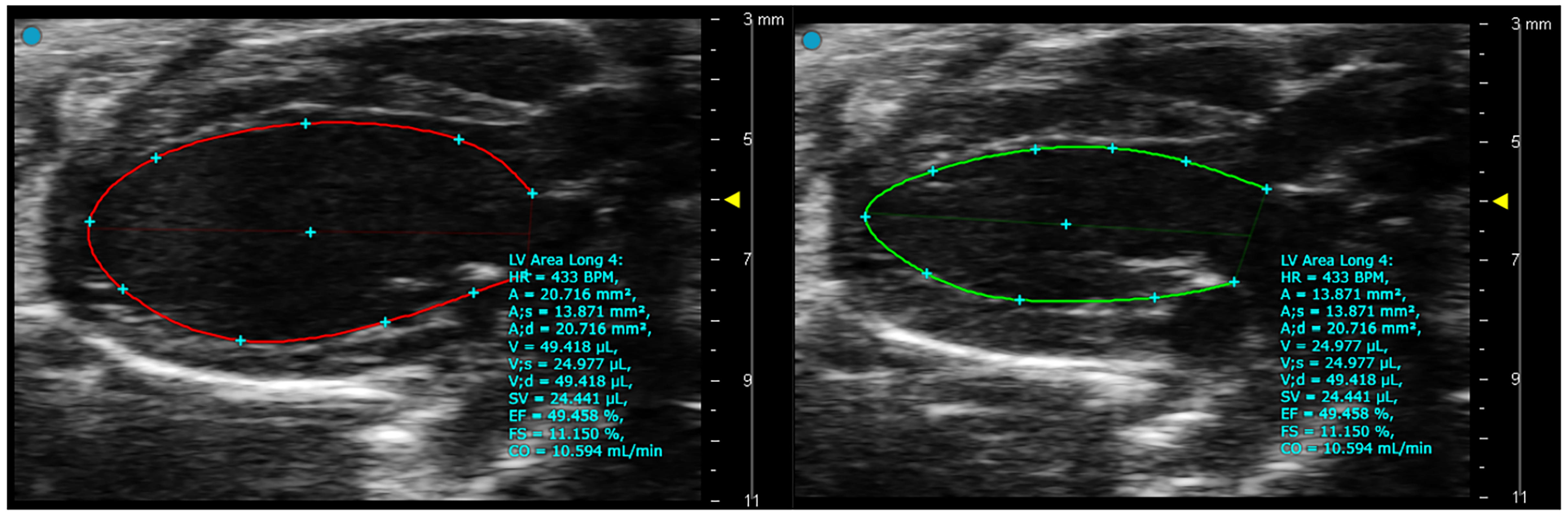

Ejection Fraction	%	49.457615
Fractional Shortening	%	11.149646
Cardiac Output	mL/min	10.594438

1w MI

P854 Stemin alone

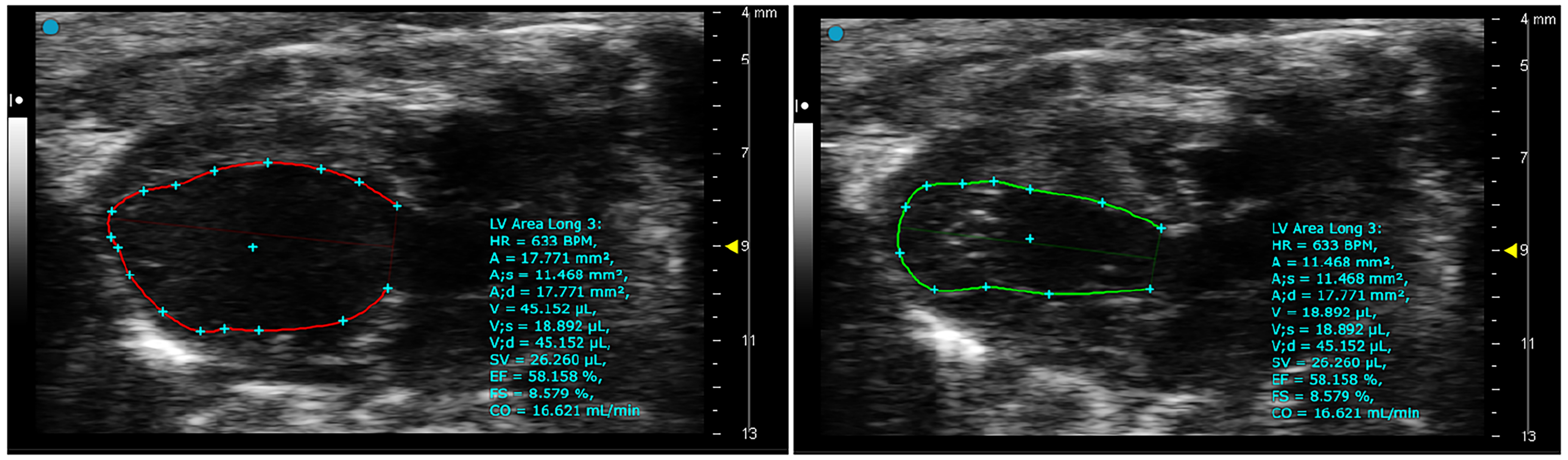

Ejection Fraction	%	58.158149
Fractional Shortening	%	8.579175
Cardiac Output	mL/min	16.621419

4w MI

P854 Stemin alone

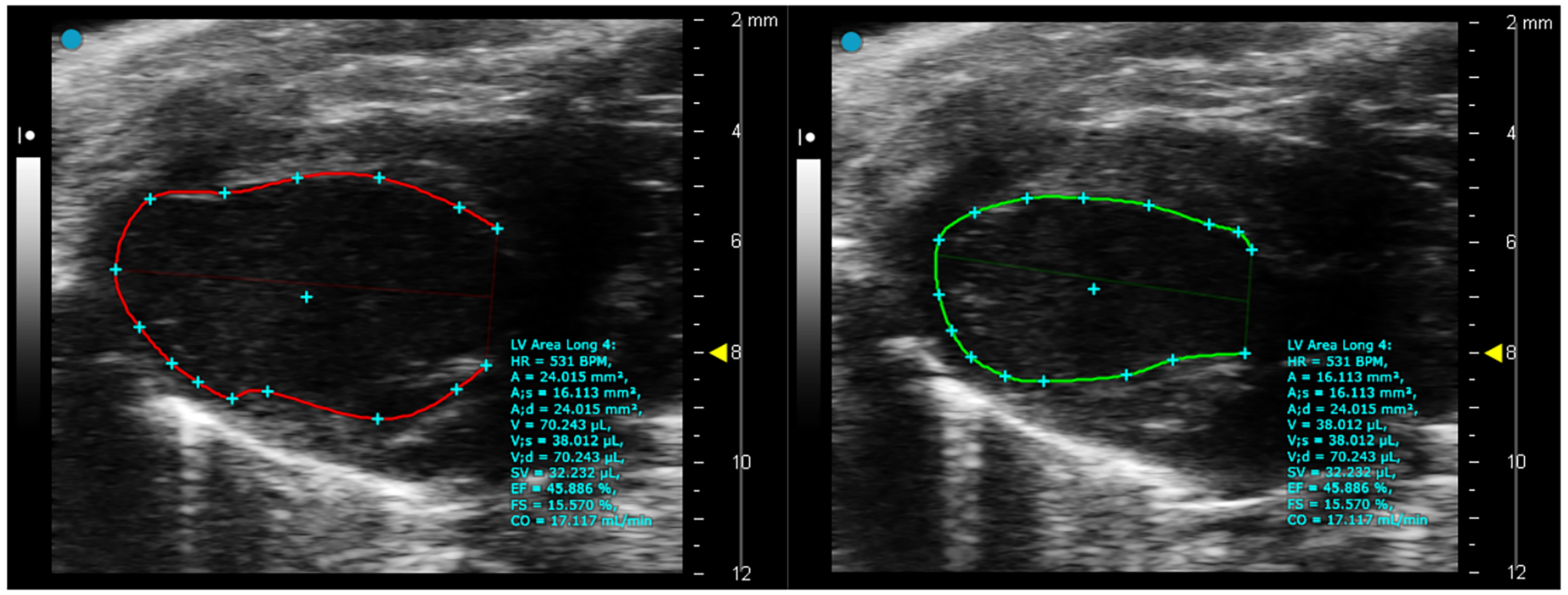

Ejection Fraction	%	45.885795
Fractional Shortening	%	15.569855
Cardiac Output	mL/min	17.117322

Before MI
Q746 YAP alone

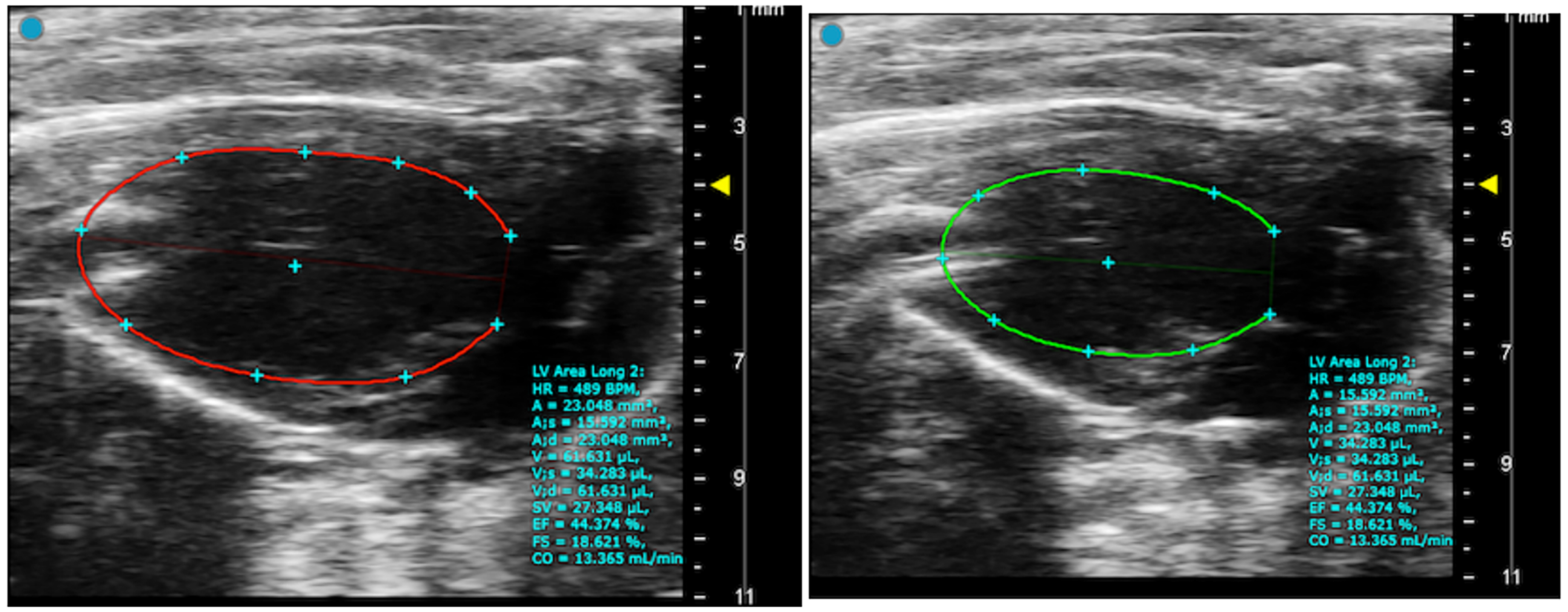

Ejection Fraction	%	44.373919
Fractional Shortening	%	18.620872
Cardiac Output	mL/min	13.365406

2 week after MI

Q746 YAP alone

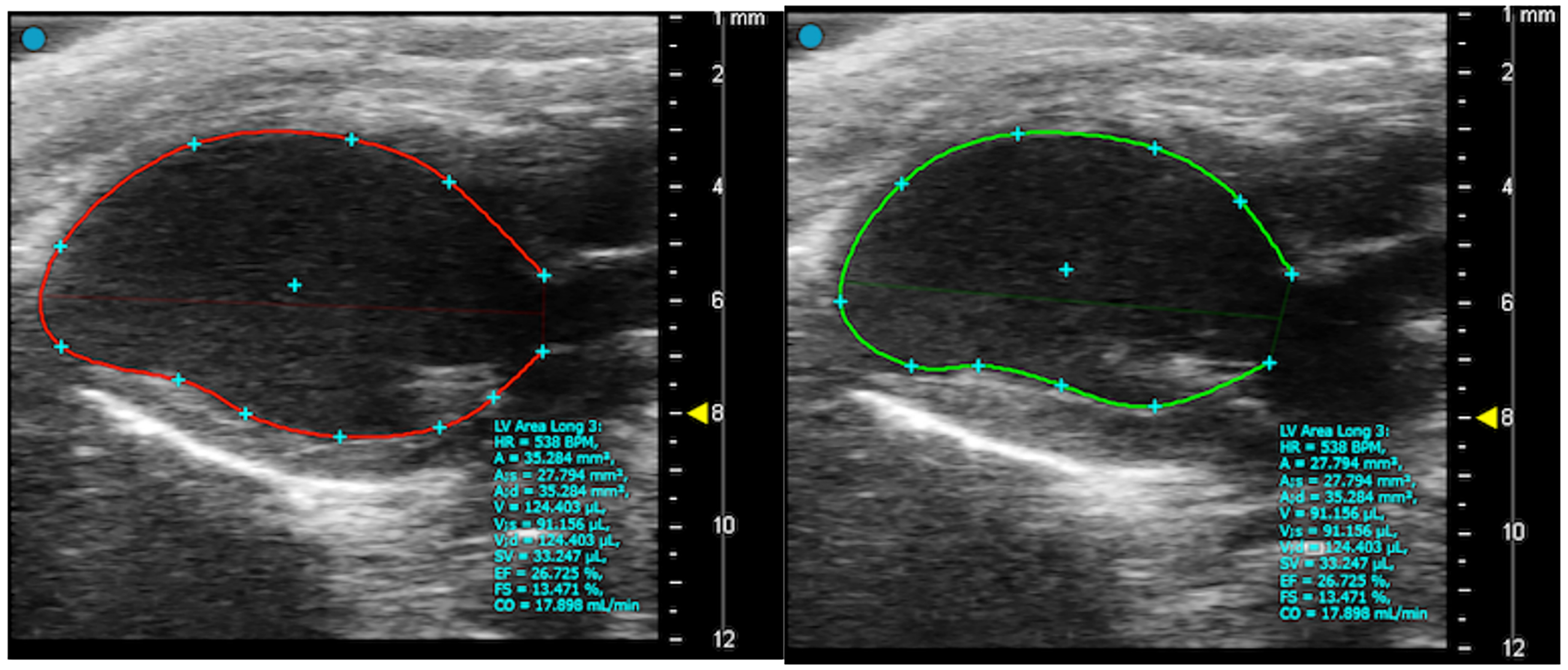

Ejection Fraction	%	26.72548
Fractional Shortening	%	13.471493
Cardiac Output	mL/min	17.897677

4 week after MI
Q746 YAP alone

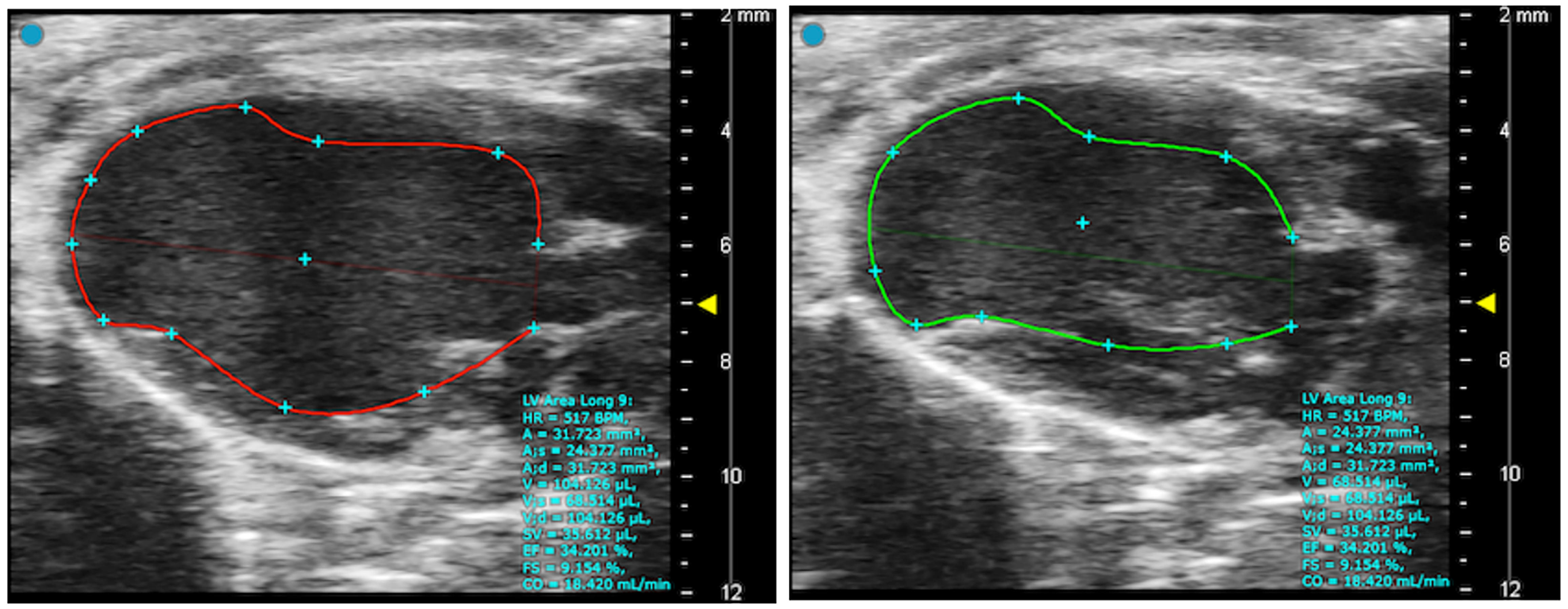

Ejection Fraction	%	34.201297
Fractional Shortening	%	9.15418
Cardiac Output	mL/min	18.420227

Before MI

Q747 YAP alone

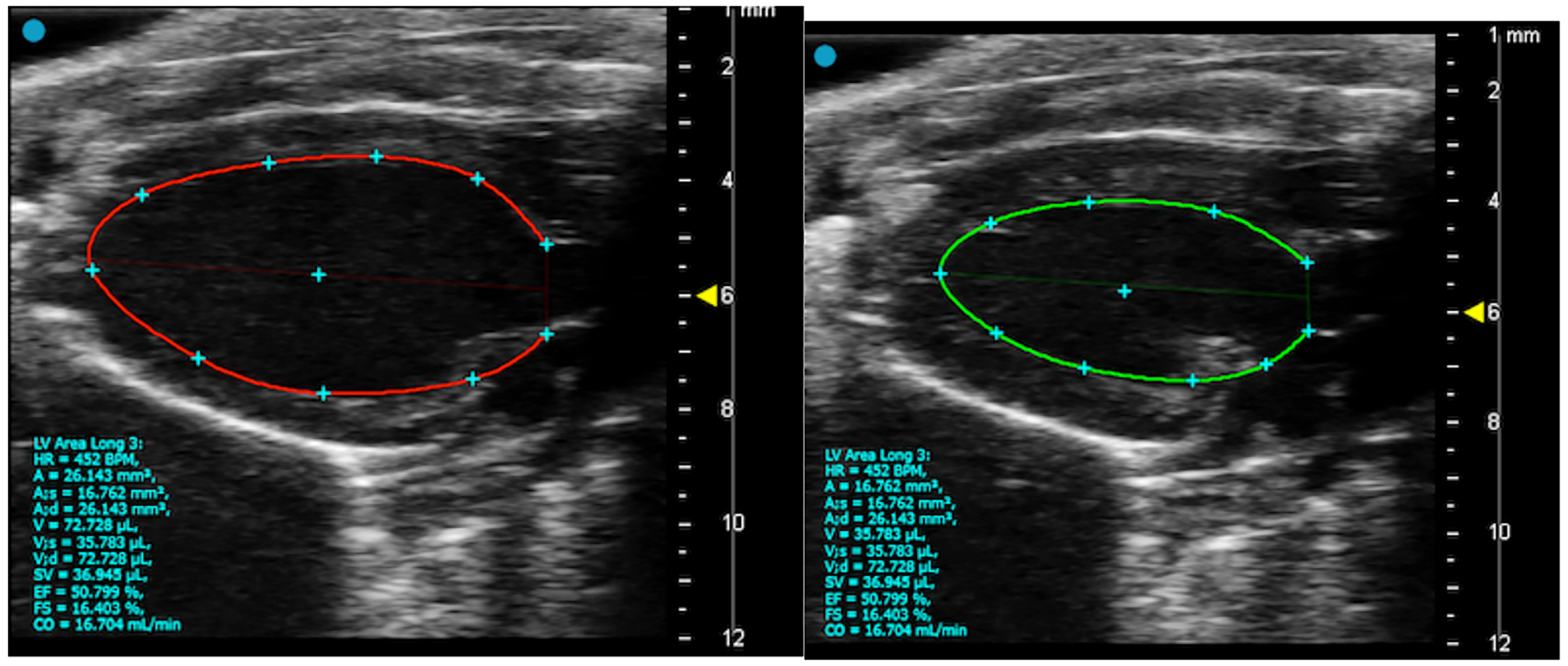

Ejection Fraction	%	50.799156
Fractional Shortening	%	16.402753
Cardiac Output	mL/min	16.703632

2 week after MI

Q747 YAP alone

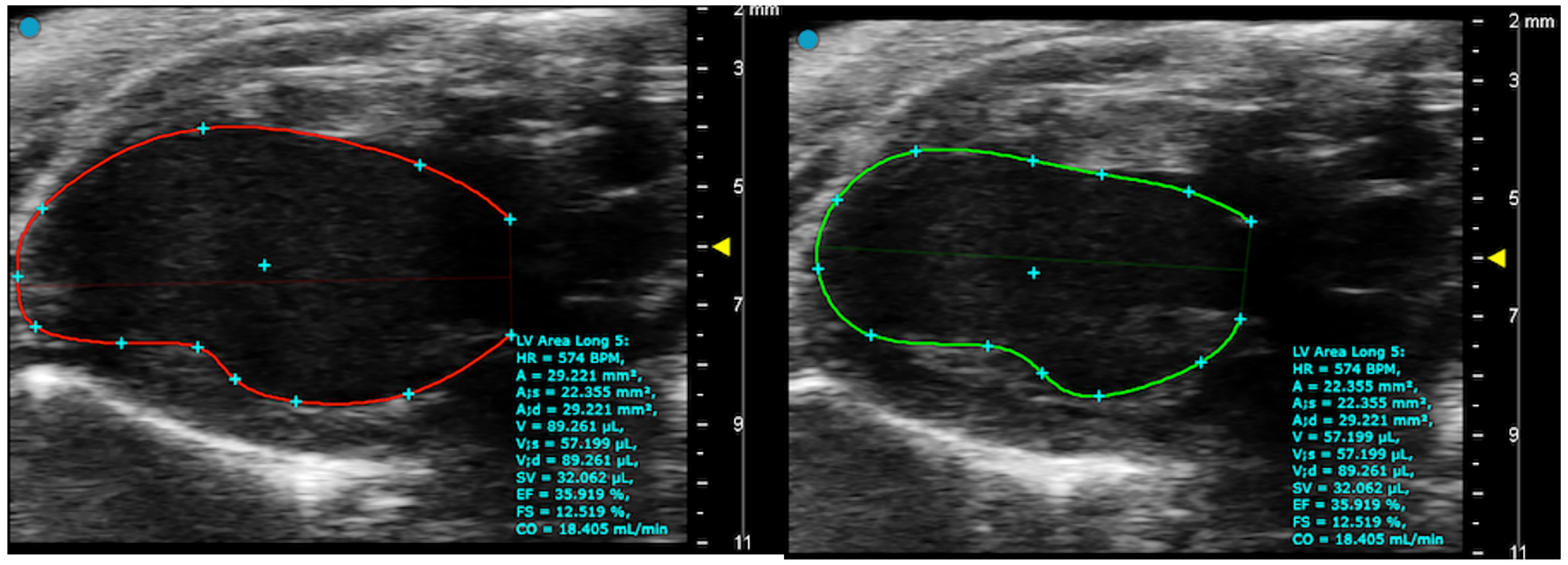

Ejection Fraction	%	35.919441
Fractional Shortening	%	12.519162
Cardiac Output	mL/min	18.40524

4 week after MI

Q747 YAP alone

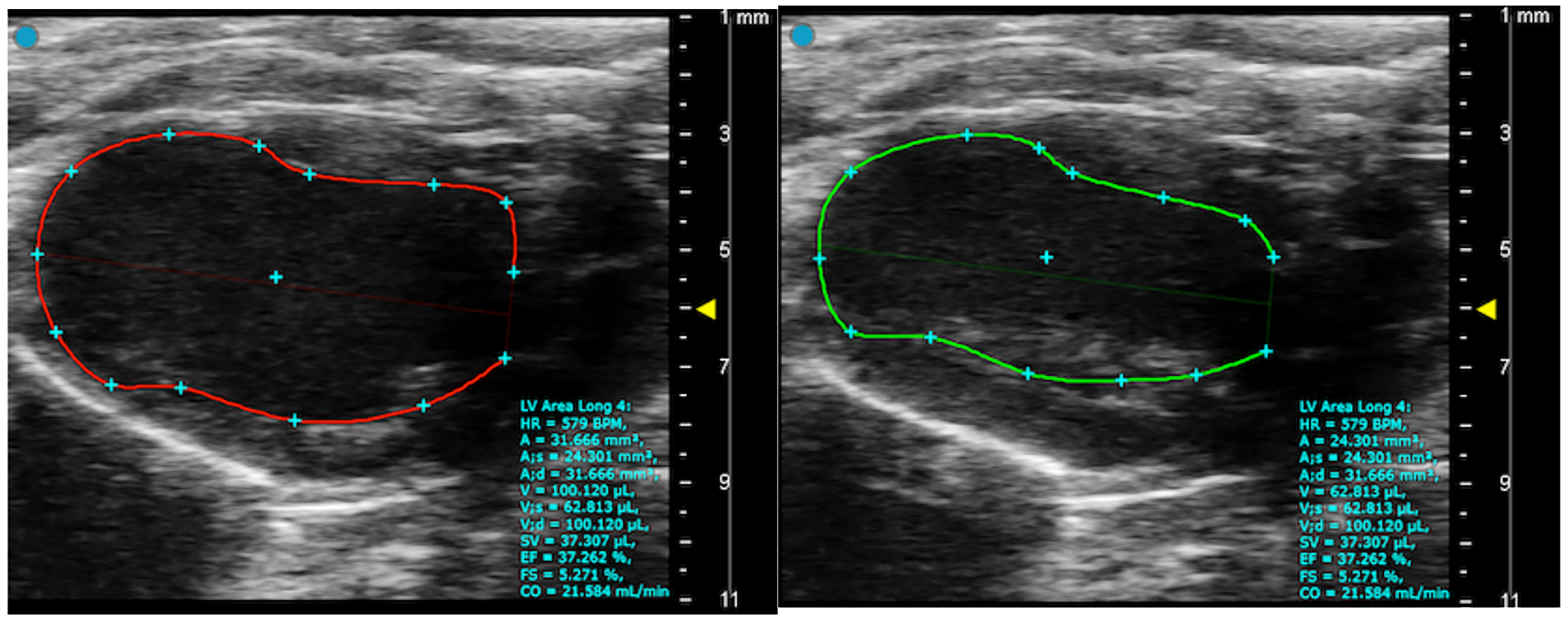

Ejection Fraction	%	37.262243
Fractional Shortening	%	5.270697
Cardiac Output	mL/min	21.583688

Before MI
Q748 YAP alone

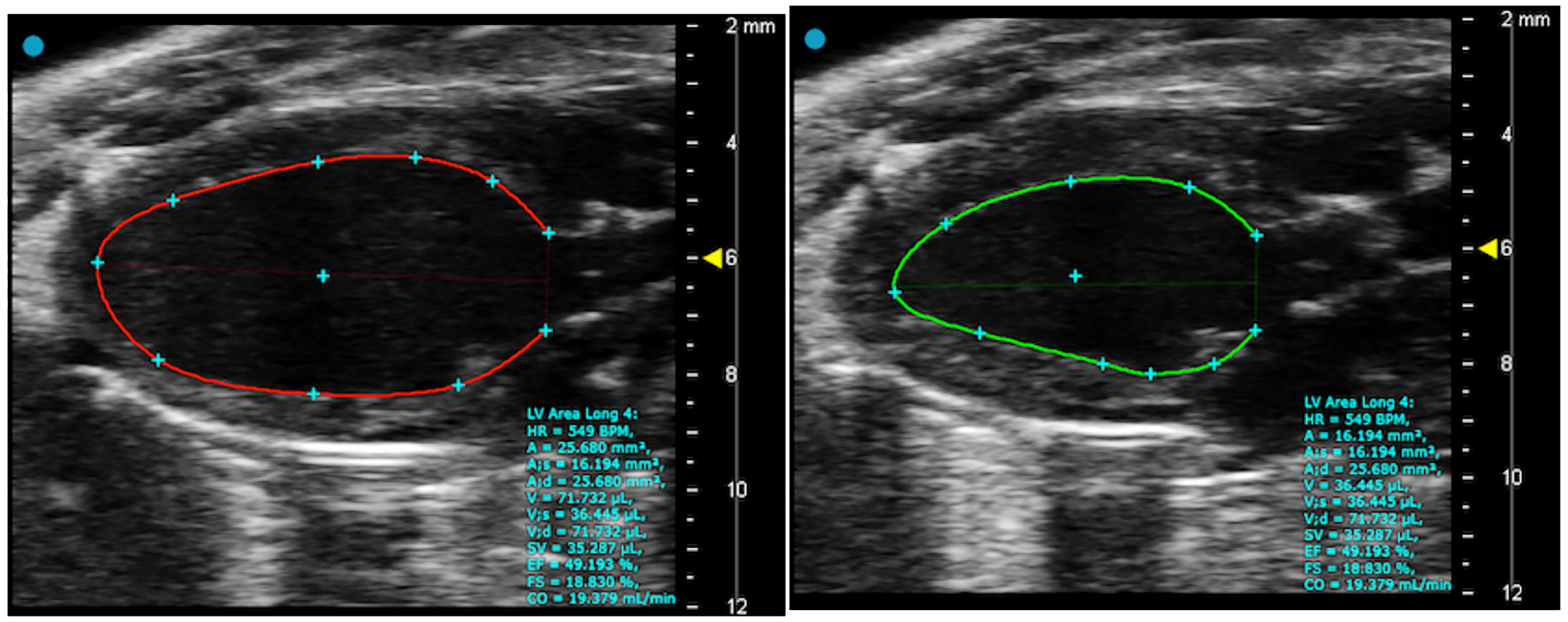

Ejection Fraction	%	49.192511
Fractional Shortening	%	18.829626
Cardiac Output	mL/min	19.379486

4 week after MI
Q748 YAP alone

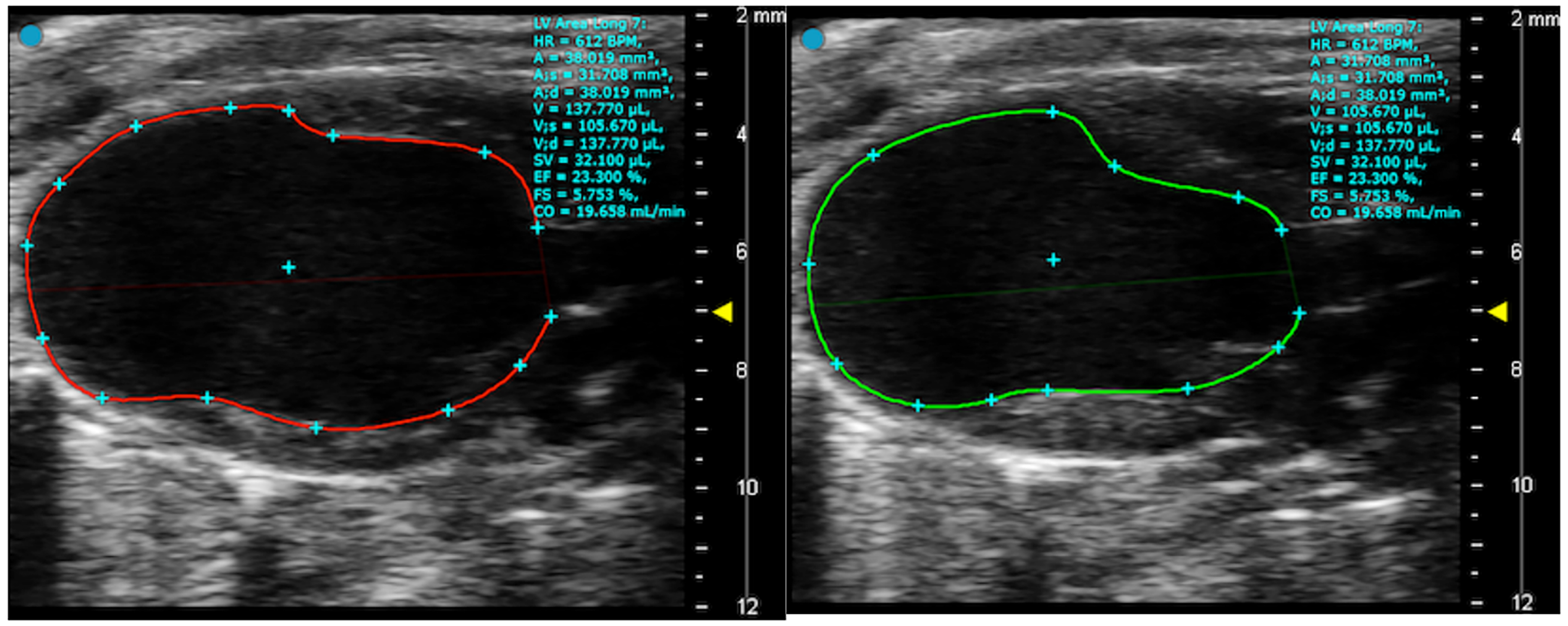

Ejection Fraction	%	23.30001
Fractional Shortening	%	5.753435
Cardiac Output	mL/min	19.657506

2 week after MI
Q748 YAP alone

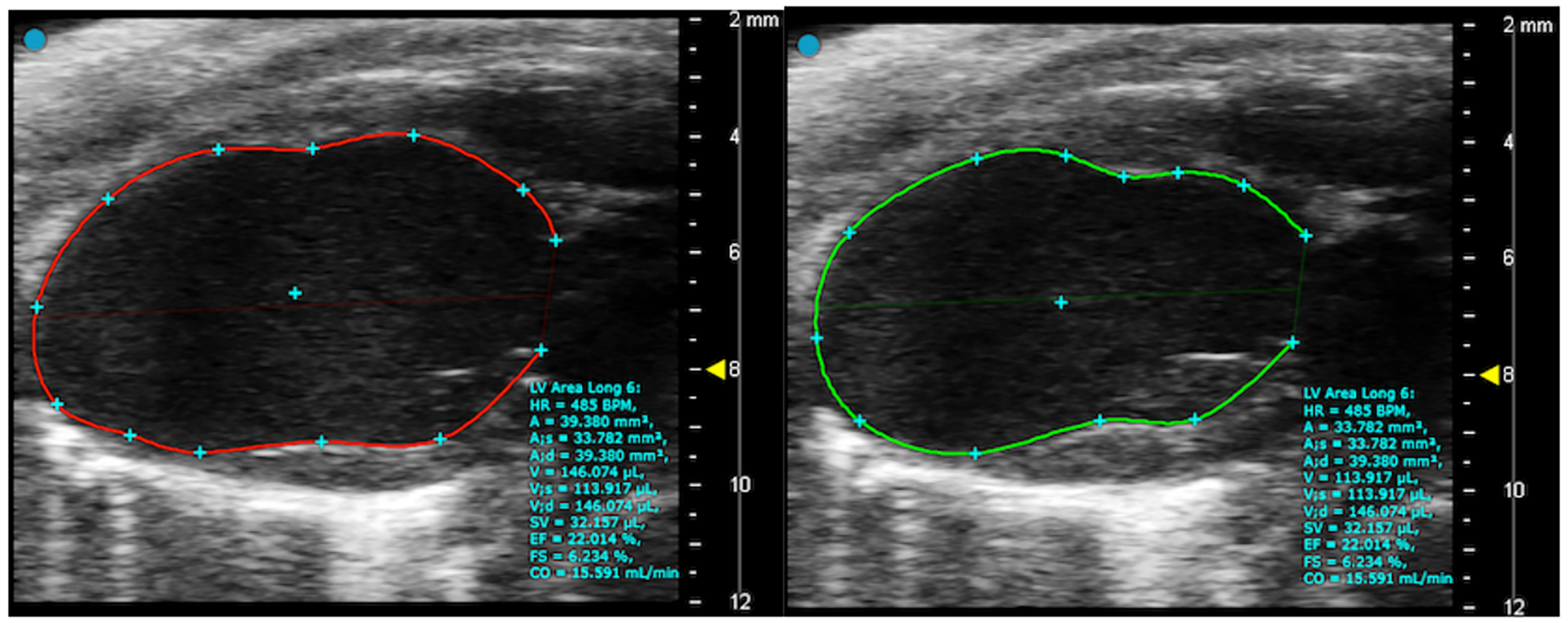

Ejection Fraction	%	22.014245
Fractional Shortening	%	6.233614
Cardiac Output	mL/min	15.591361
